# Metal Free Bi(hetero)aryl Synthesis: A Benzyne Truce–Smiles Rearrangement

**DOI:** 10.1002/anie.201510236

**Published:** 2016-01-13

**Authors:** Catherine M. Holden, Shariar M. A. Sohel, Michael F. Greaney

**Affiliations:** ^1^ School of ChemistryThe University of ManchesterOxford RoadManchesterM13 9PLUK

**Keywords:** arynes, biaryls, heterocycles, rearrangements, synthetic methods

## Abstract

A new benzyne transformation is described that affords versatile biaryl structures without recourse to transition‐metal catalysis or stoichiometric amounts of organometallic building blocks. Aryl sulfonamides add to benzyne upon fluoride activation, and then undergo an aryl Truce–Smiles rearrangement to afford biaryls with sulfur dioxide extrusion. The reaction proceeds under simple reaction conditions and has excellent scope for the synthesis of sterically hindered atropisomeric biaryl amines.

The biaryl motif is fundamental to chemistry, as it is widely represented in natural products, materials, pharmaceuticals, and agrochemicals. Synthesis of these ubiquitous structures is often carried out using transition‐metal catalysis and stoichiometric amounts of metallated arenes, which enable the coupling of two sp^2^‐carbon centers via organometallic intermediates.^1^ The drive for sustainable synthetic methods which avoid both the use of expensive and scarce transition metals, and the separate preparation of aryl metal reactants, has stimulated efforts to develop metal‐free biaryl syntheses.^2^, ^3^, ^4^ Achieving the controlled union of two arene fragments without organometallic assistance is very challenging, and current methods are often forced to employ harsh reaction conditions and/or operationally complex protocols. Recent examples include UV photochemistry to generate aryl cations from electron‐rich arenes,3a oxidative coupling of electron‐rich arenes in the presence of strong Lewis acids,3b–3d and base‐mediated coupling of aryl iodides with simple arenes through electron transfer.^4^ This latter approach has received significant attention in recent literature and uses the combination of an alkali metal base and an electron‐rich ligand to generate aryl radicals through a single‐electron transfer. Subsequent S_Ar_H substitution and oxidation lead to biaryl formation (Scheme 1 a). The method is impressively simple, but requires high temperatures, up to 20‐fold excess of the arene coupling partner, and produces regioisomeric mixtures of substituted biaryls. The discovery of a mild, metal‐free biaryl synthesis with broad substrate scope would therefore be a significant development in the field.

**Scheme 1:**
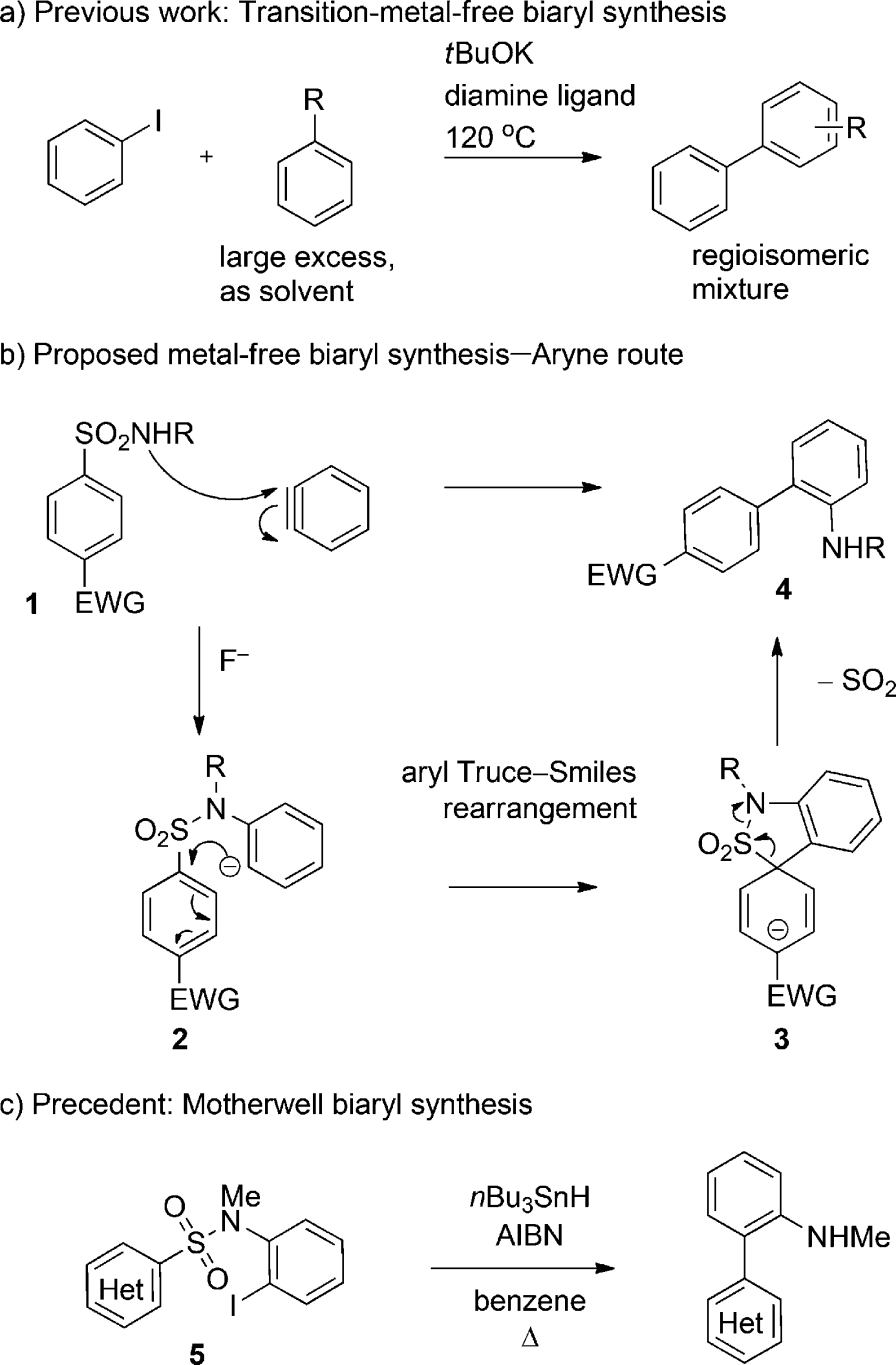
Proposed aryne addition/Truce–Smiles rearrangement for biaryl synthesis. AIBN=2,2′‐azobis(2‐methylpropionitrile), EWG=electron‐withdrawing group.

We were interested in applying the reactive intermediate benzyne to this problem. Arynes are versatile intermediates in biaryl synthesis, with their addition to aryl organometallics being a well‐described method, known since the earliest days of aryne chemistry.^5^, ^6^ Cross‐coupling with simple arenes, however, in the absence of organometallic species, has rarely been reported as a synthetic method.^7^

Given that arynes undergo smooth addition to heteroatom nucleophiles,^8^ we wondered if this feature could be exploited in a metal‐free biaryl synthesis through addition/rearrangement chemistry. Our synthetic plan is set out in Scheme 1 b. Starting with readily available aryl sulfonamides (**1**), addition of a benzyne [generated from a 2‐trimethylsilyl(phenyl) triflate precursor upon treatment with fluoride] should give the adduct **2**. This intermediate could then undergo a Smiles‐type *ipso* substitution with SO_2_ extrusion, thus affording a 2‐amino‐biaryls **4**, which are versatile molecules for further synthesis. Whilst Smiles rearrangements of stabilized carbanions are well known (the Truce variation),^9^ the rearrangement of less‐stable aryl anions as proposed here is very rare. A seminal report from Pütter and Waldau described an aryl Truce–Smiles reaction for stabilized phenolate anions, and more recent work from Quayle and co‐workers described a Truce–Smiles rearrangement of α‐silyl‐phenylsulfonate esters.^10^ Further encouragement for the idea came from the Motherwell biaryl synthesis, which demonstrates an analogous process under a radical manifold.^11^ Treatment of the haloaryl sulfonamides **5** with *n*Bu_3_SnH affords 2‐amino biaryls through radical *ipso* substitution and SO_2_ extrusion (Scheme 1 c).

As Smiles rearrangements frequently require strongly electron‐withdrawing groups on the aryl ring to stabilize the intermediate Meisenheimer complex **3**, we began work with the readily available 4‐nitrobenzenesulfonamide (**1 a**). We were pleased to discover that a screen of reaction conditions (Table 1) quickly established the reaction's viability, with the biaryl **4 a** being formed upon treatment with various fluoride sources (entries 1–4). The N‐phenylated compound was isolated in each case, thus indicating that the initial aniline product is rapidly arylated with another equivalent of benzyne.^12^ Reaction conditions of KF in the presence of 18‐crown‐6 in THF at 65 °C were optimal, thus affording 63 % of **4 a** from **1 a** and two equivalents of the benzyne precursor (entry 4). Using the N‐phenylated sulfonamide substrate **1 b** prevented the second N arylation, thus accessing **4 a** in a similar yield under the same reaction conditions but using only one equivalent of the benzyne precursor (entry 8).

**Table 1:** Reaction screening data. 
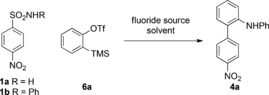

Entry	R	**1**/**6** (equiv)	Fluoride (equiv)	Yield [%]^[a]^
1	H	2:1	CsF (4)	16^[b,c]^
2	H	2:1	KF + 18‐c‐6 (4)	31^[b]^
3	H	2:1	TBAF (1)	38^[b]^
4	H	1:2	KF + 18‐c‐6 (2)	63
5	Ph	1:2	KF + 18‐c‐6 (2)	66
6	Ph	1:1	KF + 18‐c‐6 (2)	56
7	Ph	2:1	KF + 18‐c‐6 (2)	31
8	Ph	1:1	KF + 18‐c‐6 (3)	62^[d]^
9	Ph	1:1	KF + 18‐c‐6 (4)	59
10	Ph	1:1	CsF (3)	45
11	Ph	1:1	CsF (3)	64^[c]^

[a] Reaction conditions: Sulfonamide (**1**; 0.1 mmol), trimethylsilylphenyl triflate (**6**; 0.1 mmol), fluoride source, THF (0.1 m), reflux, 24 hours. Yield is that of the isolated **4 a**. [b] Ambient temperature and 16 hours. [c] Acetonitrile used as solvent. [d] Increasing scale to 1 mmol afforded **4 a** in a 56 % yield. Tf=trifluoromethanesulfonyl, TMS=trimethylsilyl.

With these simple reaction conditions in hand, we examined the scope of the reaction for a range of arynes and nitro‐substituted arylsulfonamides (**1**; Scheme 2). We were pleased to observe a versatile substrate scope: 3‐methoxy and 3‐phenyl arynes reacted with the expected regioselectivity to afford the 1,2,3‐substituted biaryls **4 b**–**d**. 1,2‐Naphthyne was likewise productive, but with a diminished regioselectivity of 3:1 (**4 e** major regioisomer; X‐ray). 4‐Substituted arynes gave the biaryls **4 f**–**i** in good yields as 1:1 mixtures, thus implicating the benzyne intermediate in the reaction mixture. *Ortho* substitution was well tolerated on the sulfonamide partner (**4 j**–**l**), and it was possible to switch the nitro activating group to this position, thus giving **7 a** in 72 % yield. 2,6‐Dichloro substitution was particularly effective, thus giving an excellent 81 % yield of **4 l**. Substitution adjacent to the *para*‐nitro group was also possible, but some attrition in yield was observed. The bulky CF_3_ group produced a poor yield of **4 n** and is likely a result of steric inhibition of the resonance stabilization for the Meisenheimer intermediate. The reaction also accommodated N‐alkyl sulfonamides. In contrast to the N‐aryl substrates, a second arylation with benzyne was observed to give the tertiary biaryl aniline products **7 a**–**f**. Restricted rotation around the biaryl axis would be expected for some of the more hindered products, and this was established for **4 d** and **7 f**, by HPLC analysis using a chiral stationary phase, thus establishing the 1:1 ratio of atropisomers in each case.

**Scheme 2:**
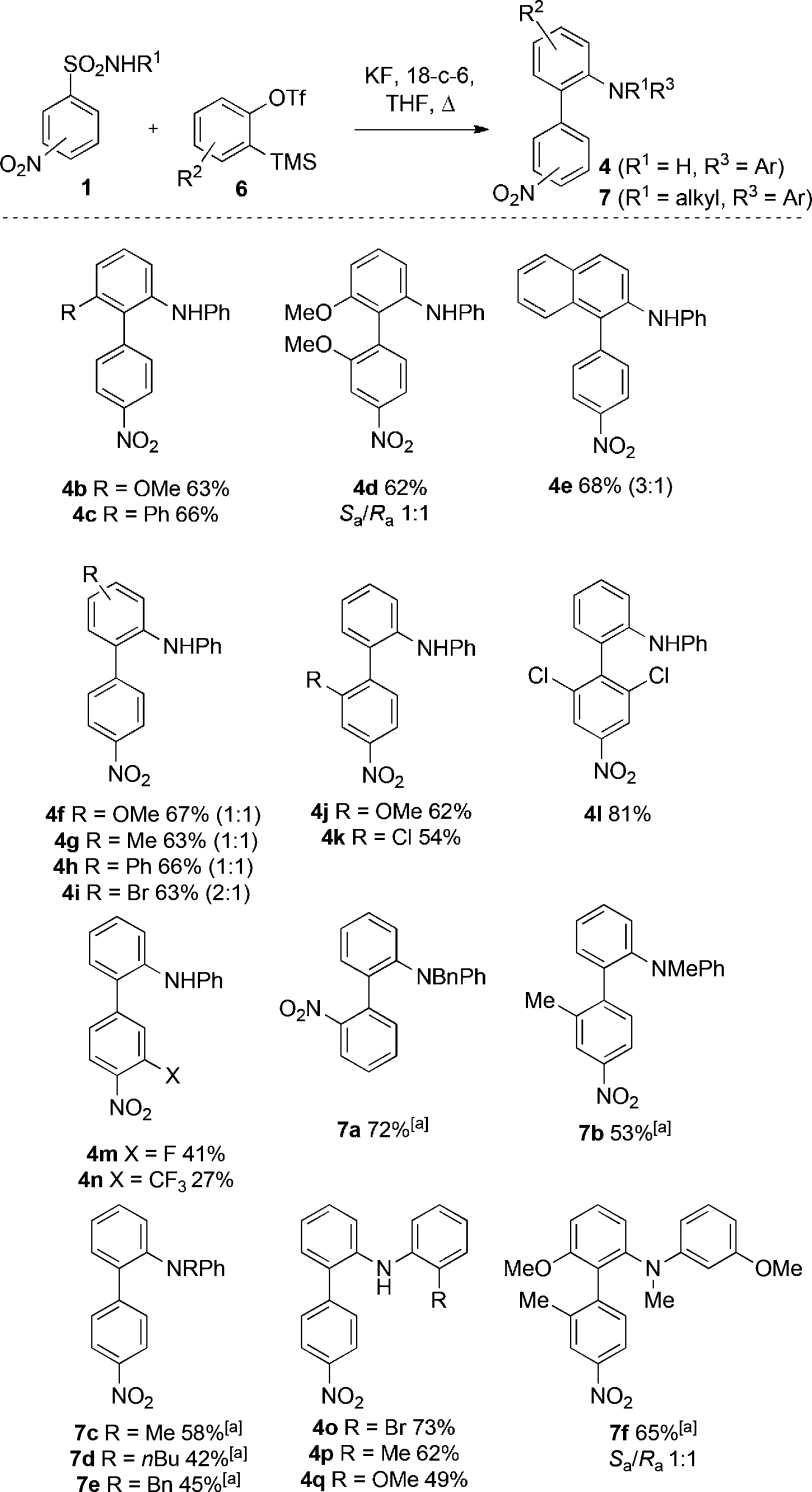
Substrate scope using nitro phenylsulfonamides. Yields are those of isolated products. [a] Two equivalents of **6** used.

The *ortho*‐amino functionality in the biaryl products is nicely disposed for additional heterocycle formation (Scheme 3). The bromophenyl product **4 o** proved a good substrate for C−H activation, thus affording the carbazole **8 a** in excellent yield on treatment with catalytic palladium(0).^13^ Alternatively, a denitrative S_N_Ar cyclization of **4 r** using NaH/HMPA gave the carbazole **8 b**.^14^ The nitro group represents a versatile access point for further nitrogen‐based functionality, and we could achieve an iterative biaryl synthesis by initial reduction, followed by sulfonamide formation with *p*‐nitrophenylsulfonyl chloride (NsCl). Aryne Truce–Smiles rearrangement was then successful, thus tolerating the substrate aniline, proceeding in a similar efficiency to that of the first, and producing the extended biaryl **10** in 67 % yield.

**Scheme 3:**
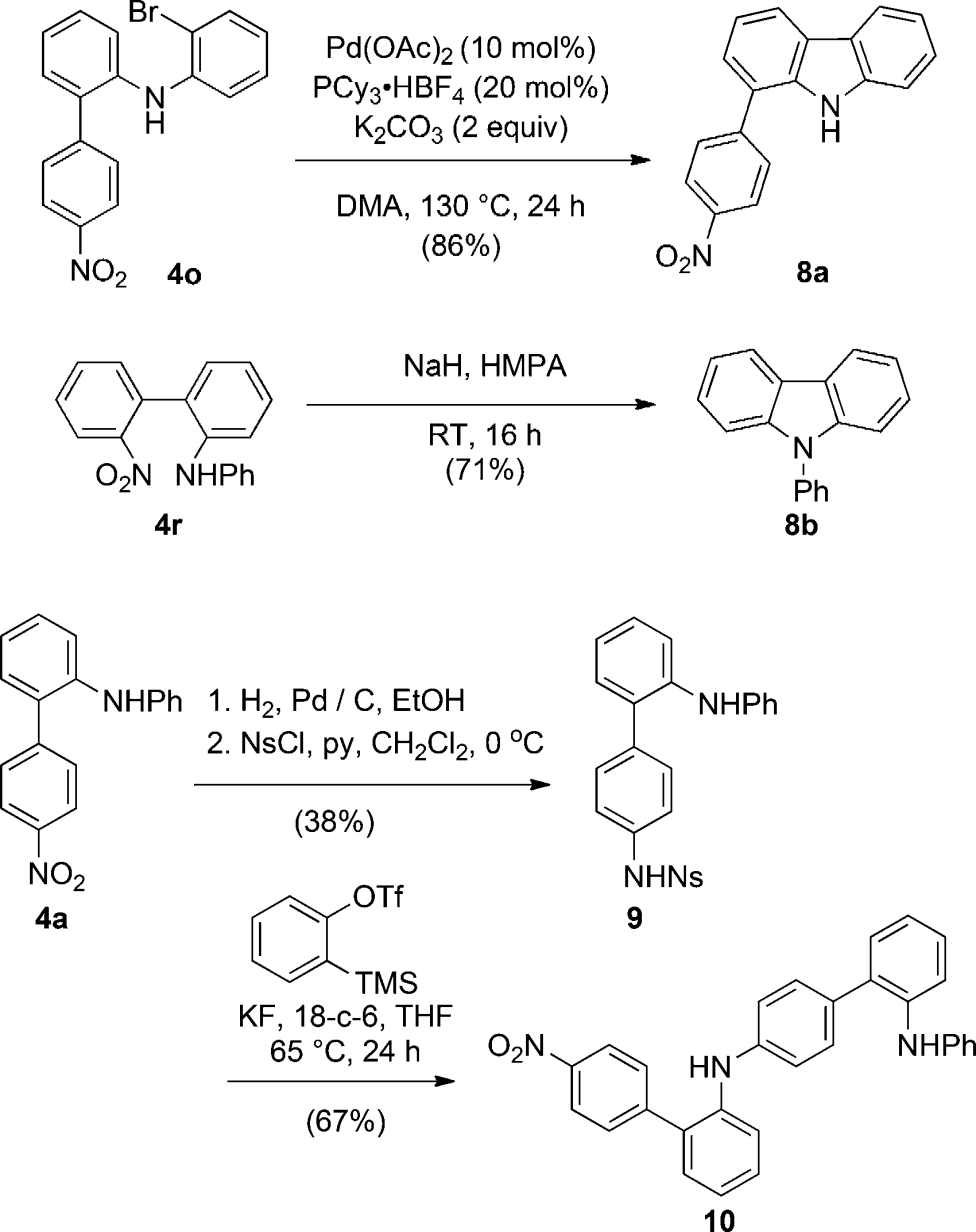
Heterocycle synthesis. Yields are those of isolated products. DMA=*N*,*N*‐dimethylacetamide, HMPA=hexamethylphosphoramide, NsCl=*p*‐nitrophenylsulfonyl chloride.

Having established the scope of the reaction for nitro‐substituted sulfonamides, we were keen to develop a more general procedure with broad substrate scope (Scheme 4). Pleasingly, we found that alternative electron‐withdrawing groups such as cyano and acyl were effective in the reaction (**4 s** and **4 t**), along with the triply halogenated 4‐bromo‐2,6‐dichloro motif (**4 u**; X‐ray). Indeed, this substrate gave higher yields than the analogous nitro‐containing compounds. Using an α‐methyl benzylamine sulfonamide gave the secondary aryl Truce–Smiles product **4 x**, which could be deprotected to the primary aniline product **11**.

**Scheme 4:**
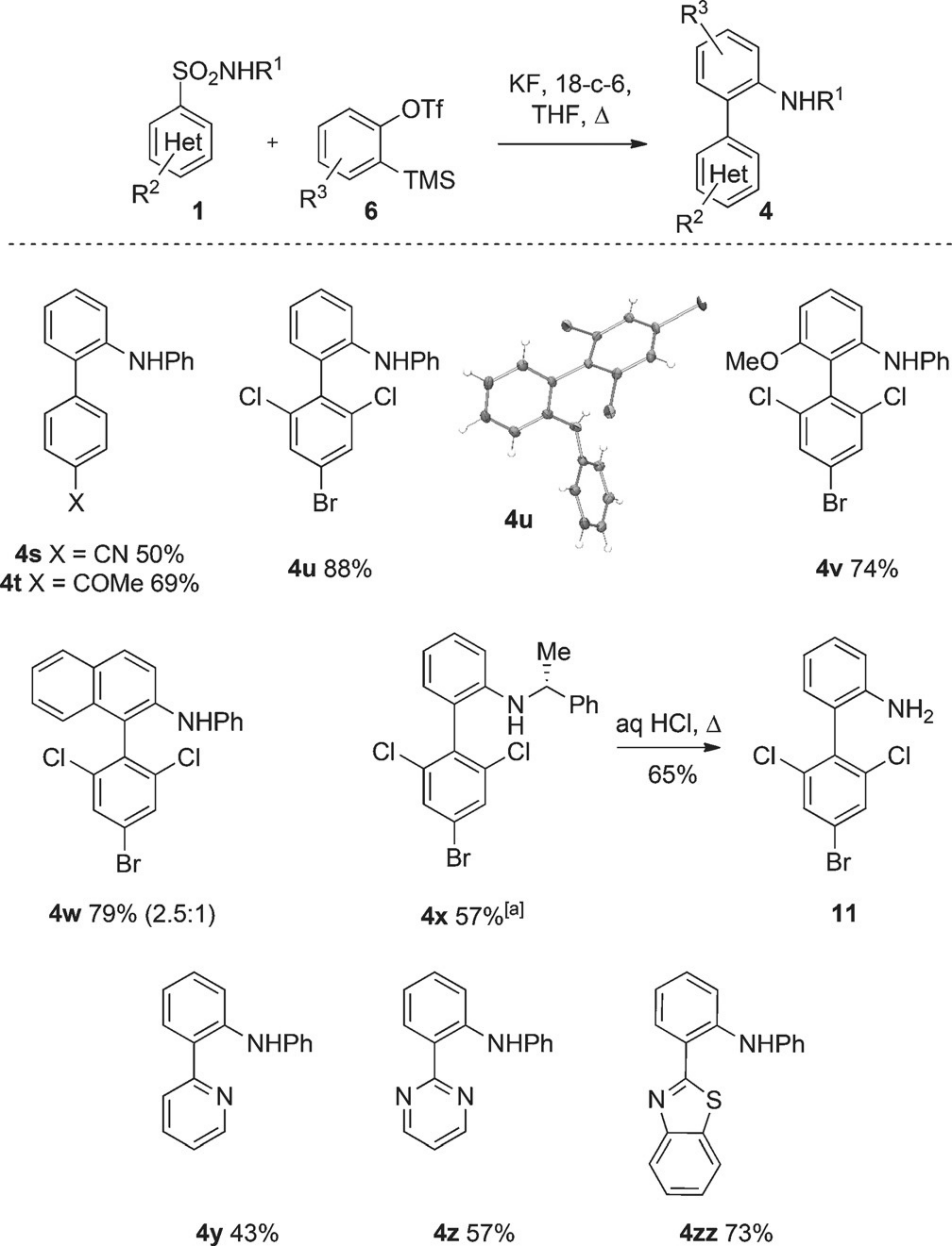
Substrate scope using other electron‐deficient sulfonamides. Yields are those of isolated products. [a] Two equivalents of **6** used.^16^

The facility of the reaction for accessing sterically congested, tetra‐*ortho*‐substituted biaryls is notable, and compliments traditional metal cross‐coupling methods,^15^ which are often challenged by such steric hindrance because of the requirement for oxidative addition and/or transmetalation processes of large metal–ligand complexes. In terms of limitations, tertiary sulfonamides were unreactive under the reaction conditions, and some degree of activation was required on the Smiles acceptor arene; simple *para*‐chloro, *para*‐bromo, and *para*‐methyl derivatives were recovered unchanged from the reaction.

Finally, we examined heteroarenes in the reaction. The Smiles rearrangement of heteroaromatic sulfones is key to the Julia–Kocienski olefination reaction, involving S→O migration, and has been demonstrated on several interesting heteroarene substrates such as benzothiazoles, tetrazoles, and benzimidazoles.^17^ An analogous S→Csp^2^ rearrangement in our system would be a new entry point into heterobiaryl structures under metal‐free conditions, something we were keen to investigate. We were pleased to find that the analogous heteroaromatic sulfonamides were productive under the same reaction conditions of KF/18‐c‐6, and we could prepare the arylated pyridine, pyrimidine, and benzothiazole derivatives **4 y**–**zz** in moderate to good yield (Scheme 4).

In conclusion, we have demonstrated that metal‐free biaryl synthesis is possible under mild reaction conditions, through a novel sulfonamide–benzyne addition and subsequent aryl Truce–Smiles rearrangement. The reaction is notably effective for accessing sterically hindered, tri‐ and tetra‐*ortho*‐substituted biaryls, which are difficult to synthesize using traditional cross‐coupling methods. Applications of this benzyne transformation will be the subject of future investigations in our laboratory.

## Supporting information

As a service to our authors and readers, this journal provides supporting information supplied by the authors. Such materials are peer reviewed and may be re‐organized for online delivery, but are not copy‐edited or typeset. Technical support issues arising from supporting information (other than missing files) should be addressed to the authors.

Supplementary
