# Supplementary material for: Metal Free Bi(hetero)aryl Synthesis: A Benzyne Truce–Smiles Rearrangement
Source: Angew Chem Int Ed Engl. 2016 Jan 13;55(7):2450–3. doi: 10.1002/anie.201510236 (PMC4819888; doi:10.1002/anie.201510236)

Angewandte Chemie
Eine Zeitschrift der Gesellschaft Deutscher Chemiker

Supporting Information

Metal Free Bi(hetero)aryl Synthesis: A Benzyne Truce–Smiles Rearrangement

*Catherine M. Holden, Shariar M. A. Sohel, and Michael F. Greaney**

anie_201510236_sm_miscellaneous_information.pdf

Supporting Information

Contents

1. General Information: ... 4
2. Synthesis of Sulfonamide Starting Materials ... 5
General procedure A for synthesis of *N*-aryl sulfonamides ... 5
General procedure B for synthesis of *N*-aryl sulfonamides ... 5
General procedure C for the synthesis of *N*-alkyl sulfonamides ... 5
3. Characterisation data for sulfonamides ... 6
Compound 1b ... 6
Compound 1c ... 6
Compound 1d ... 7
Compound 1e ... 8
Compound 1f ... 8
Compound 1g ... 9
Compound 1h ... 9
Compound 1i ... 11
Compound 1j ... 11
Compound 1k ... 12
Compound 1l ... 12
Compound 1m ... 13
Compound 1n ... 13
Compound 1o ... 14
Compound 1p ... 14
Compound 1q ... 15
Compound 1r ... 15
Compound 1s ... 15
Compound 1t ... 16

Compound 1u 17
4. General Procedures for Synthesis of Biaryls 18
General Procedure D 18
General Procedure E 18
Characterisation data for biaryls 19
Nitro-phenyl sulfonamide scope (Scheme 2) 19
Compound 4a 19
Compound 4b 19
Compound 4c 20
Compounds 4d 21
Compound 4e’ 22
Compound 4e’’ 22
Compounds 4f 23
Compounds 4g 24
Compounds 4h 25
Compounds 4i 26
Compound 4j 26
Compound 4k 27
Compounds 4l 27
Compounds 4m 28
Compound 4n 29
Compound 4o 29
Compound 4p 30
Compound 4q 30
Compound 4r 31
Compound 7a 31
Compound 7b 32
Compound 7d 33
Compound 7e 34
Compound 7f 34
Sulfonamide Scope (Scheme 4) 35
Compound 4s 35
Compound 4t 36

Compound 4u 36
Compound 4v 37
Comound 4w...38
Compound 4x 38
Compound 4y 39
Compound 4z...40
5. Characterisation data for derivative compounds 41
Compound 8a 41
Compound 8b 41
Compound 9 43
Compound 11 44
6. References..45
7. NMR Spectra……………………………………………………………………46

1. General Information:

Nuclear Magnetic Resonance (NMR) spectra were recorded on 500 or 400 MHz Bruker NMR spectrometers in $CDCl_3$ at 298 K (unless stated otherwise). All chemical shift values are reported in parts per million (ppm) with coupling constant (*J*) values reported in Hz. All spectra were referenced to $CDCl_3$ the residual solvent peak $CHCl_3$ (δ = 7.26 ppm) for 1H NMR and the $CDCl_3$ solvent peak (δ = 77.16 ppm) for $^{13}C\{^{1}H\}$ NMR. The notation of signals is: Proton: δ chemical shift in ppm (number of protons, multiplicity, *J* value(s), proton assignment). Carbon: δ chemical shift in ppm (carbon assignment). Fluorine: δ chemical shift in ppm (Fluorine assignment). Splitting patterns are assigned s = singlet, b = broad, d = doublet, td = triplet of doublet, dt = doublet of triplet, t = triplet, q = quartet.

Low resolution mass spectrometry was performed on an Agilent 6100 mass spectrometer (ES ionisation) and Hewlett Packard 5971 MSD (GC/MS with EI). High resolution mass spectrometry was performed on a Waters QTOF with ESI/APCI ionisation and a Thermo Finnigan MAT95XP (EI).

Chiral stationary phase HPLC was performed using an Aglient 1200 Series HPLC with a Chirapak IB 4.6 × 250 mm column and eluted with 1% isopropyl alcohol and 99% hexane at a rate of 1mL/min with absorbance reported at 254 nm.

Melting points were determined using a Kofler hot-stage apparatus or Stuart Scientific SMP10 apparatus and are uncorrected. Thin layer chromatography (TLC) was performed using pre-coated Merck 60F254 silica plates. Visualization was performed using UV light (285 nm) and treatment with acidic potassium permanganate. Flash chromatography was performed using silica gel (Sigma Aldrich, 40-63 μ, 60 Å) and a Biotage® Isoleara™ equipped with 10 g or 25 g Biotage® SNAP Ultra cartridges.

Tetrahydrofuran (THF) was distilled over sodium wire and benzophenone. CH_2Cl_2 and toluene and distilled over calcium hydride. Diethyl ether was dried using a solvent purification system. All other solvents and reagents were purchased from commercial sources and used as supplied.

2. Synthesis of Sulfonamide Starting Materials

General procedure A for synthesis of *N*-aryl sulfonamides

N-Aryl sulfonamides were synthesised according to the following modified literature procedure.[1] The sulfonyl chloride (1 eq.) was dissolved in ethanol (0.5 M) and the respective aniline (2 eq.) was added. The reaction mixture was stirred and monitored by TLC analysis (1:3 *v/v* EtOAc:hexane). Upon completion the reaction mixture was acidified with HCl (1 M) and stirred at 0 °C for 5 minutes and then was then diluted with EtOAc and water. The aqueous phase was washed twice with EtOAc, and the combined organics were washed with brine, dried over magnesium sulfate, filtered and concentrated under vacuum. The crude reaction mixture was then purified by column chromatography.

General procedure B for synthesis of *N*-aryl sulfonamides

N-Aryl sulfonamides were prepared by following the literature procedure.[2] A solution of sulfonyl chloride (1.0 mmol) in dry CH_2Cl_2 (15 mL) was added over 15 min to a solution of amine (1.1 mmol) and pyridine (1.1 mmol) in dry CH_2Cl_2 (15 mL). The reaction was stirred at ambient temperature for 2 – 3 hours and the progress of the reaction was monitored by TLC (1:3 *v/v* EtOAc:hexane). Upon completion the reaction mixture was acidified with HCl (1 M, pH 2), the aqueous phase was separated and extracted with CH_2Cl_2 (2 × 10 mL). The combined organic layers were washed with brine, dried over Na_2SO_4, and evaporated to dryness to give sulfonamide as a crystalline solid. The crude material was purified by column chromatography.

General procedure C for the synthesis of *N*-alkyl sulfonamides

N-Alkyl sulfonamides were synthesised according to modified literature procedures.[3] The sulfonyl chloride (1 mmol, 1 eq.) was dissolved in THF (0.2 M) and appropriate amine (3 eq.) was added to the reaction mixture. The reaction was stirred for 30 minutes at ambient temperature until completion was indicated by TLC (1:3 *v/v* EtOAc:hexane). The reaction mixture as acidified with 1 M HCl (pH 2) at 0 °C, then diluted with EtOAc and water. The aqueous phase was washed twice with EtOAc and the combined organics were washed with saturated brine and dried over magnesium sulfate, filtered and concentrated under vacuum. The crude product was triturated with EtOAc and hexane, filtered and dried under vacuum or purified by column chromatography.

3. Characterisation data for sulfonamides

Compound 1b

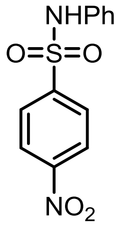

Chemical Formula: $C_{12}H_{10}N_2O_{4S}$
MW = 278

4-Nitro-*N*-phenylbenzenesulfonamide **1b** was synthesised according to general procedure A to afford a white crystalline solid (218 mg, 78%). R_f 0.41 (EtOAc:hexanes 1:3); ^{1}H NMR (400 MHz, DMSO-d_6) δ 10.61 (s, 1H, NH), 8.37 (d, *J* = 8.9 Hz, 2H, ArH), 7.99 (d, *J* = 8.9 Hz, 2H, ArH), 7.27 (d, *J* = 7.2 Hz, 1H, ArH), 7.25 (d, *J* = 7.2 Hz, 1H, ArH), 7.13 – 7.04 (m, 3H, ArH); ^{13}C NMR (101 MHz, DMSO-d_6) δ 149.8 (C), 144.9 (C), 136.9 (C), 129.4 (2 x CH), 128.3 (2 x CH), 124.8 (CH), 124.7 (2 x CH), 120.7 (2 x CH); *m/z* (ES$^-$) 277 ([M-H]$^-$, 100%). Data are consistent with literature values.[3]

Compound 1c

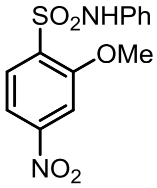

Chemical Formula: $C_{13}H_{12}N_2O_5S$
MW = 308

2-Methoxy-4-nitro-*N*-phenylbenzenesulfonamide **1c** was synthesized according to general procedure A (1.19 mmol) to give an off-white crystalline solid (301 mg, 82%). R_f 0.19 (EtOAc: hexane 1:4) = 0.19; ^{1}H NMR (400 MHz, $CDCl_3$) δ 8.01 (d, *J* = 8.6 Hz, 1H, ArH), 7.85 – 7.82 (m, 2H, ArH), 7.24 – 7.20 (m, 2H, ArH), 7.11 (d, *J* = 7.4 Hz, 1H, ArH), 7.07 – 7.03 (m, 3H, ArH), 4.17 (s, 3H, OCH_3); ^{13}C NMR (101 MHz, $CDCl_3$) δ 156.6 (C), 151.6 (C), 135.6 (C), 132.1 (CH), 131.9 (C), 129.4 (2 x CH), 125.9 (CH), 121.4 (2 x CH), 115.5 (CH), 107.2 (CH), 57.2 (OCH_3); HRMS (TOF MS ES$^-$) *m/z* calculated for $C_{13}H_{11}N_2O_5S$ 307.0389 $[M-H]^+$, found 307.0382; mp 154 °C.

Compound 1d

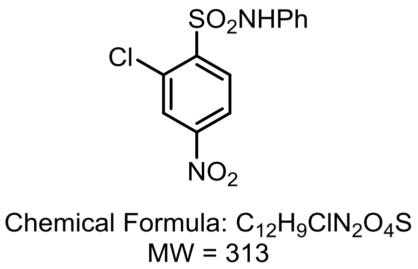

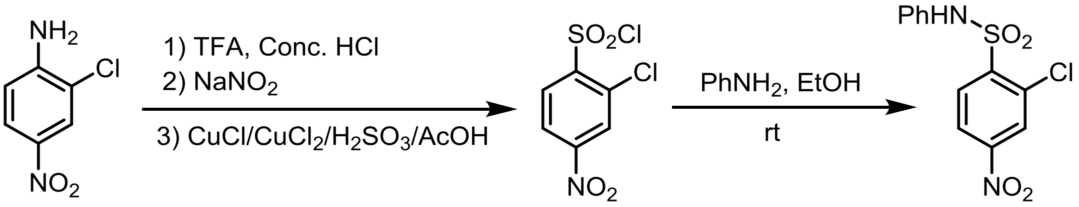

Conc. HCI (4 mL) was added to a solution of 2-chloro-4-nitroaniline (2.10 g, 12.1 mmol) in TFA (40 mL). The mixture was cooled to 0 °C and then a solution of sodium nitrite (1.06 g, 15.4 mmol) in water (3 mL) was added over a 20 minute period maintaining the temperature at 0°C. After 20 minutes the reaction mixture was poured into a solution of CuCl (80 mg), $CuCl_2$ (0.826 g, 6.2 mmol) and H_2SO_3 (40 ml) in acetic acid (40 mL) and kept at 0°C. After the initial effervescence subsided, the reaction mixture was allowed to sit at room temperature. After 30 minutes, the reaction mixture was diluted with 200 mL of water and extracted with hexane (2 × 100 mL). The combined organics were evaporated under vacuum to give the crude sulfonyl chloride as an amber oil (1.75 g). The sulfonyl chloride was used directly in the next step without further purification.

2-Chloro-4-nitro-N-phenylbenzenesulfonamide **1d** was synthesized according general procedure A to afford an off-white crystalline solid (1.21 g, 32% over two steps). R_f 0.39 (EtOAc:hexane 1:4); ^{1}H NMR (400 MHz, $CDCl_3$) δ 8.36 (d, *J* = 2.1 Hz, 1H, ArH), 8.19 (d, *J* = 8.7 Hz, 1H, ArH), 8.14 (d, *J* = 8.7, 2.1 Hz, 1H, ArH), 7.27 – 7.22 (m, 2H, ArH), 7.16 – 7.10 (m 4H, ArH); ^{13}C NMR (126 MHz, DMSO-d_6) δ 150.2 (C), 141.9 (C), 136.3 (C), 132.8 (CH), 131.9 (C), 129.3 (2 x CH), 126.7 (CH), 124.5 (CH), 122.7 (CH), 119.8 (2 x CH); HRMS (TOF MS ES$^-$) *m/z* calculated for $C_{12}H_8N_2O_4SCl$ 310.9893 [M-H]$^-$, found 310.9889; Mp: 98 °C.[4]

Compound 1e

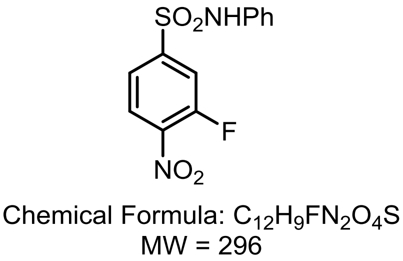

3-Fluoro-4-nitro-*N*-phenylbenzenesulfonamide **1e** was synthesized according to general procedure A (0.835 mmol) to give a yellow crystalline solid (239 mg, 97%). R_f 0.28 (EtOAc: hexane 1:4); ^{1}H NMR (500 MHz, $CDCl_3$) δ 8.09 (dd, *J* = 10.0 , 6.9 Hz, 1H, ArH), 7.69 (dd, *J* = 9.6, 1.8 Hz, 1H, ArH), 7.67 – 7.64 (m, 1H, ArH), 7.34 – 7.30 (m, 2H, ArH), 7.24 – 7.21 (m, 1H, ArH), 7.11– 7.08 (m, 2H, ArH), 6.78 (brs, 1H, NH); ^{13}C NMR (126 MHz, $CDCl_3$) δ 155.0 (d, *J* = 271.0 Hz, CF), 145.5 (d, *J* = 6.6 Hz, *meta*-C), 139.9 (m, *ortho*-C), 134.9 (CH), 129.8 (2 x CH), 127.0 (d, *J* = 2.7 Hz, *para*-CH), 126.9 (CH), 123.3 (d, *J* = 5.0 Hz, *meta*-CH), 122.6 (2 x CH), 117.8 (d, *J* = 23.7 Hz, *ortho*-CH); ^{19}F NMR (500 MHz, $CDCl_3$) δ -113.54; HRMS (TOF MS ES$^-$) *m/z* calculated for $C_{12}H_8N_2O_4SF$ 295.0189 [*M*-H]$^-$, found 295.0201; mp: 116 °C.

Compound 1f

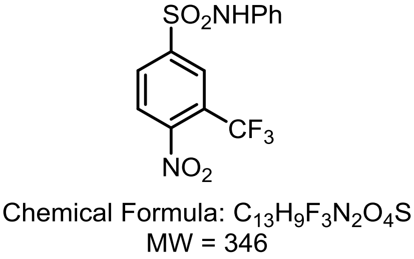

4-Nitro-*N*-phenyl-3-(trifluoromethyl)benzenesulfonamide **1f** was synthesized according to general procedure A (1.05 mmol) to give a white crystalline solid (272 mg, 76%). R_f 0.35 (EtOAc:hexane 1:4); ^{1}H NMR (500 MHz, $CDCl_3$) δ 8.18 (d, *J* = 1.9 Hz, 1H, ArH), 8.08 (dd, *J* = 8.4, 1.9 Hz, 1H, ArH), 7.90 (d, *J* = 8.4 Hz, 1H, ArH), 7.35 (brs, 1H, NH), 7.32 – 7.29 (m, 2H, ArH), 7.24 – 7.21 (m, 1H, ArH), 7.12 – 7.10 (m, 2H, ArH); ^{13}C NMR (126 MHz, $CDCl_3$) δ 150.0 (C), 143.0 (C), 134.7 (C), 132.0 (CH), 129.8 (2 x CH), 127.0 (q, *J* = 5.2 Hz, CH), 126.9 (CH), 125.8 (CH), 124.6 (q, *J* = 35.3 Hz, **C**-CF_3), 122.6 (2 x CH), 120.9 (q, *J* = 275.0 Hz, CF_3); ^{19}F NMR (500 MHz, $CDCl_3$) δ -60.26; HRMS (TOF MS AP$^+$) *m/z* calculated for $C_{13}H_{10}N_2O_4SF_3$ [*M*+H]$^+$ 347.0313, found 347.0303; Mp: 62 °C.

Compound 1g

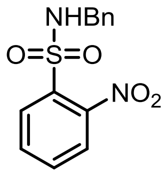

Chemical Formula: $C_{13}H_{12}N_2O_4S$
MW = 292

N-Benzyl-2-nitrobenzenesulfonamide **1g** was synthesized according to general procedure A (1 mmol) to give a white crystalline solid (252 mg, 86%). R$_f$ 0.14 (EtOAc:hexane 1:3); ^{1}H NMR (400 MHz, $CDCl_3$) δ 8.00 (dd, *J* = 8.0, 1.6 Hz, 1H, ArH), 7.82 (dd, *J* = 7.8, 1.5 Hz, 1H, ArH), 7.70 – 7.61 (m, 2H, ArH), 7.23 – 7.20 (m, 5H, ArH), 5.71 (t, *J* = 6.3 Hz, 1H, NH), 4.32 (d, *J* = 6.3, 2H, CH_2); ^{13}C NMR (101 MHz, $CDCl_3$) δ 147.8, 135.6, 134.0, 133.4, 132.7, 131.0, 128.7, 128.0, 127.8, 125.2, 47.9; HRMS (ES$^+$) *m/z* calculated for $C_{13}H_{12}N_2O_4NaS$ $[M+Na]^+$ 315.0415, found : 315.0426; mp: 92 °C. Data are consistent with literature.[5]

Compound 1h

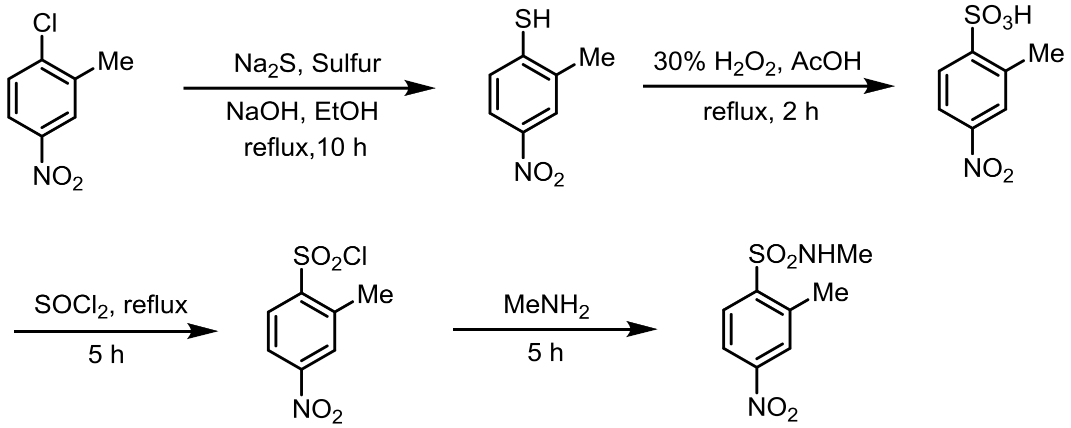

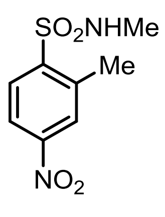

Chemical Formula: $C_{13}H_{12}N_2O_4S$
MW = 230

N,2-Dimethyl-4-nitrobenzenesulfonamide **1h** was synthesised according to modified a modified literature procedure.**[6]**

A mixture of 2-chloro-5-nitrotoluene (1.0 g, 5.8 mmol), 60% purity grade Na_2S (0.55 g, 4.2 mmol), sulfur (0.136 g, 4.2 mmol), NaOH (0.233 g, 5.8 mmol) in 50 mL ethanol was refluxed for 10 h. The reaction was quenched with 10 mL 10% HCl. The reaction mixture was extracted twice with ethyl acetate (25 mL) and the organic layer was washed with 10% HCl, dried (anhydrous Na_2SO_4), filtered and evaporated in vacuo. The crude product 2-methyl-4-nitrobenzenethiol (0.556 g, 56%) was obtained as a yellow solid which was used in the following step without further purification. R_f 0.36 (EtOAc:hexanes 1:1); ^{1}H NMR (500 MHz, $CDCl_3$) δ 8.02 (d, *J* = 2.4 Hz, 1H, ArH), 7.92 (dd, *J* = 8.5, 2.4 Hz, 1H, ArH), 7.34 (d, *J* = 8.5 Hz, 1H, ArH), 3.68 (brs, 1H, SH), 2.39 (s, 3H, CH_3); ^{13}C NMR (126 MHz, $CDCl_3$) δ 145.5, 141.9, 136.0, 129.0, 124.8, 121.6, 20.8; HRMS (TOF MS ES^-) *m/z* calculated for $C_7H_6NO_2S$ 168.0119 [*M*-H]$^-$, found 168.0120; mp: 172 °C.

A mixture of 2-methyl-4-nitrobenzenethiol (0.556 g), 6% H_2O_2 (14.3 mL, 25.3 mmol) and AcOH (5.4 g, 90.5 mmol) was refluxed for 2 h. The completion of reaction was confirmed by TLC. The crude product 2-methyl-4-nitrobenzenesulfonic acid was obtained upon evaporation of the reaction mixture under reduced pressure. The crude 2-methyl-4-nitrobenzenesulfonic acid appeared as a light yellow crystalline solid (0.657g, 92%) and was used without further purification. R_f 0.74 (MeOH:$CHCl_3$ 5:95); ^{1}H NMR (400 MHz, $CDCl_3$) δ 8.07 (d, *J* = 2.4 Hz, 1H, ArH), 8.01 (dd, *J* = 8.8, 2.4, 1H, ArH), 7.58 (d, *J* = 8.8, 1H, ArH), 2.58 (s, 3H, CH_3); ^{13}C NMR (101 MHz, $CDCl_3$) δ 146.3, 142.2, 137.1, 125.8, 125.0, 122.2, 19.1.

To 2-methyl-4-nitrobenzenesulfonic acid (0.657 g) was added $SOCl_2$ (1.2 mL, 16.3 mmol) and the mixture was refluxed for 5 hours. Excess $SOCl_2$ was evaporated in vacuo to give the acid chloride as a black solid which was used in the following step directly.

To a THF (10 mL) solution of 2-methyl-4-nitrobenzenesulfonyl chloride (crude product), was added an ethanolic solution of $MeNH_2$, (33% solution by wt, 1.7 mL, 18.4 mmol) and the reaction mixture was stirred for 5 hours at ambient temperature. The volatiles were evaporated in vacuo, and the crude material was extracted with ethyl acetate. The organic layer was dried over (anhydrous Na_2SO_4), filtered and evaporated to give the crude sulfonamide. The crude product was subsequently purified by column chromatography (SiO_2, 1:9 *v/v* EtOAc:hexane). The pure sulfonamide was obtained as a pale yellow crystalline solid (140 mg, 20% over two steps). R_f 0.48 (EtOAc:hexanes 1:1); ^{1}H NMR (400 MHz, $CDCl_3$) δ 8.19 (s, 1H, ArH), 8.16 – 8.15 (m, 2H, ArH), 4.51 (brq, *J* = 5.4 Hz, 1H, ArH), 2.76 (s, 3H,

NCH_3), 2.71 (d, *J* = 5.4, 3H, CH_3); ^{13}C NMR (101 MHz, $CDCl_3$) δ 149.9, 142.9, 139.4, 130.9, 127.2, 121.1, 29.2, 20.6; HRMS (TOF MS ES$^-$) calculated for $C_8H_9N_2O_4S$ 229.0283 [*M*-H]$^+$ found 229.0285; mp: 166 °C.

Compound 1i

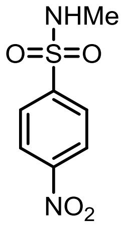

Chemical Formula: $C_7H_8N_2O_4S$
MW = 216

N-Methyl-4-nitrobenzenesulfonamide **1i** was synthesised according to general procedure C to afford a white crystalline solid (182 mg, 84%). R_f 0.17 (EtOAc:hexane 1:3); ^{1}H NMR (400 MHz, DMSO-d_6) δ 8.43 (d, *J* = 8.8 Hz, 2H, ArH), 8.02 (d, *J* = 8.8 Hz, 2H, ArH), 7.84 (s, 1H, NH), 2.46 (s, 3H, CH_3); ^{13}C NMR (101 MHz, DMSO-d_6) δ 149.6 (C), 144.9 (C), 128.3 (2 x CH), 124.7 (2 x CH), 28.6 (CH_3); *m/z* (ES$^-$) 215 ([M-H]$^-$, 100%). Data are consistent with literature values.[3]

Compound 1j

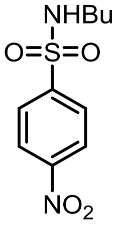

Chemical Formula: $C_{10}H_{14}N_2O_4S$
MW = 258

N-Butyl-4-nitrobenzenesulfonamide **1j** was synthesised according to general procedure C to afford a white crystalline solid (181 mg, 70%). R_f 0.39 (EtOAc:hexane 1:3); ^{1}H NMR (400 MHz, DMSO-d_6) δ 8.41 (d, *J* = 8.7 Hz, 2H, ArH), 8.03 (d, *J* = 8.7 Hz, 2H, ArH), 7.96 (t, *J* = 5.9 Hz, 1H, NH), 2.78 (q, *J* = 6.6 Hz, 2H, N$\mathbf{CH_2}$$CH_2CH_2CH_3$), 1.34 (p, *J* = 6.9 Hz, 2H, $NCH_2$$\mathbf{CH_2}$$CH_2CH_3$), 1.22 (h, *J* = 7.2 Hz, 2H, $NCH_2CH_2$$\mathbf{CH_2}$$CH_3$), 0.79 (t, *J* = 7.3 Hz, 3H, $NCH_2CH_2CH_2$$\mathbf{CH_3}$); ^{13}C NMR (101 MHz, DMSO-d_6) δ 149.5 (C), 146.2 (C), 128.0 (2 x CH), 124.6 (2 x CH), 42.2 (N**C**H_2 $CH_2CH_2CH_3$), 31.1(NCH_2**C**$H_2CH_2CH_3$), 19.2($NCH_2CH_2$$\mathbf{CH_2}$$CH_3$), 13.4 ($NCH_2CH_2CH_2$$\mathbf{CH_3}$); *m/z* (ES$^-$) 257.1 ([*M*-H]$^-$, 100%). Data are consistent with literature values.[7]

Compound 1k

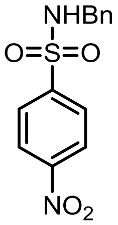

Chemical Formula: $C_{13}H_{12}N_2O_4S$
MW = 292

N-Benzyl-4-nitrobenzenesulfonamide **1k** was synthesised according to general procedure C to afford a white crystalline solid (225 mg, 77%). R_f 0.32 (EtOAc:hexanes 1:3); ^{1}H NMR (400 MHz, DMSO-d_6) δ 8.57 (t, *J* = 6.2 Hz, 1H, NH), 8.36 (d, *J* = 8.9 Hz, 2H, ArH), 8.00 (d, *J* = 8.9 Hz, 2H, ArH), 7.30 – 7.16 (m, 5H, ArH), 4.06 (d, *J* = 6.2 Hz, 2H, CH_2); ^{13}C NMR (101 MHz, DMSO-d_6) δ 149.4 (C), 146.4 (C), 137.1 (C), 128.3 (2 x CH), 128.1 (2 x CH), 127.7 (2 x CH), 127.3 (CH), 124.5 (2 x CH), 46.2 (CH_2); *m/z* (ES^-) 291 ([*M*-H]$^-$, 100%). Data are consistent with literature values.[8]

Compound 1l

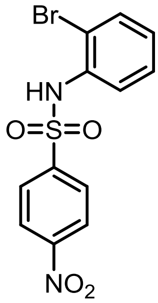

Chemical Formula: $C_{12}H_9BrN_2O_4S$
MW = 357

N-(2-Bromophenyl)-4-nitrobenzenesulfonamide **1l** was synthesised according to general procedure A to afford a pink solid (191 mg, 54%). R_f 0.46 (EtOAc:hexane 1:3); ^{1}H NMR (400 MHz, DMSO-d_6) δ 10.40 (s, 1H, NH), 8.40 (d, *J* = 8.9 Hz, 2H, ArH), 7.94 (d, *J* = 8.9 Hz, 2H, ArH), 7.61 (dd, *J* = 8.4, 1.5 Hz, 1H, ArH), 7.35 (td, *J* = 7.6, 1.5 Hz, 1H, ArH), 7.23 – 7.16 (m, 2H, ArH); ^{13}C NMR (101 MHz, DMSO-d_6) δ 149.8 (C), 146.0 (C), 134.2 (C), 133.4 (CH), 128.9 (CH), 128.9 (CH), 128.6 (CH), 128.3 (2 x CH), 124.7 (2 x CH), 120.9 (C); HRMS (TOF MS ES^-) *m/z* calculated for $C_{12}H_8BrN_2O_4S$ 354.9388 [*M*-H]$^-$, found 354.9379; mp 136 – 138 °C.

Compound 1m

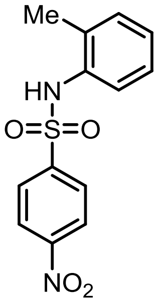

Chemical Formula: $C_{13}H_{12}N_2O_4S$
MW = 292

4-Nitro-*N*-(*o*-tolyl)benzenesulfonamide was synthesized according to general procedure B to give a pinkish white crystalline solid (277 mg, 94%). R_f 0.28 (EtOAc:hexanes 1:3); ^{1}H NMR (400 MHz, $CDCl_3$) δ 8.28 (d, *J* = 8.8, 2H, ArH), 7.90 (d, *J* = 8.8, 2H, ArH), 7.28 – 7.26 (m, 1H, ArH), 7.20 – 7.12 (m, 3H, ArH), 6.52 (brs, 1H, NH), 2.02 (s, 3H, CH_3); ^{13}C NMR (101 MHz, $CDCl_3$) δ 150.2, 145.3, 133.3, 132.1, 131.1, 128.4, 127.3, 127.2, 125.0, 124.3, 17.7; *m/z* (ES^-) 291 ($[M-H]^-$, 100%); Mp: 152 °C. Data are consistent with literature.[3]

Compound 1n

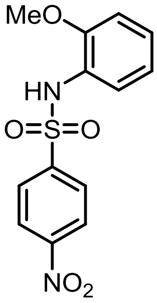

Chemical Formula: $C_{13}H_{12}N_2O_5S$
MW = 308

N-(2-Methoxyphenyl)-4-nitrobenzenesulfonamide was synthesized according to general procedure B to give a pale yellow crystalline solid (253 mg, 82%). R_f 0.28 (EtOAc:hexane 1:3); ^{1}H NMR (400 MHz, DMSO-d_6) δ 9.95 (s, 1H, NH), 8.36 (d, *J* = 8.7, 2H, ArH), 7.92 (d, *J* = 8.7, 2H, ArH), 7.21 (dd, *J* = 8.0, 1.6 Hz, 1H, ArH), 7.17 (t, *J* = 8.0 Hz, 1H, ArH), 6.91 – 6.88 (m, 2H, ArH), 3.41 (s, 3H, OCH_3); ^{13}C NMR (101 MHz, DMSO-d_6) δ 153.0, 149.5, 146.3, 128.2, 127.7, 126.9, 124.3, 124.1, 120.5, 111.9, 55.2; *m/z* (ES^-) 307 ($[M\text{-}H]^-$, 100%); Mp 142 °C.[3]

Compound 1o

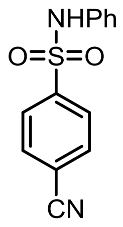

Chemical Formula: $C_{13}H_{10}N_2O_4S$
MW = 258

4-Cyano-*N*-phenylbenzenesulfonamide **1o** was synthesised according to general procedure A to afford a white solid (240 mg, 93%). R_f 0.08 (EtOAc:hexanes 1:9); ^{1}H NMR (500 MHz, $CDCl_3$) δ 7.88 (d, *J* = 8.5 Hz, 2H, ArH), 7.76 (d, 2H, ArH), 7.33 – 7.27 (m, 3H, ArH), 7.21 (t, *J* = 7.4 Hz, 1H, ArH), 7.09 (d, *J* = 7.9 Hz, 2H, ArH), 6.78 (s, 1H, NH); ^{13}C NMR (101 MHz, $CDCl_3$) δ 144.2 (C), 143.0 (C), 140.1 (C), 132.7 (2 x CH), 130.8 (CH), 130.2 (2 x CH), 130.0 (C), 129.5 (2 x CH), 121.9 (CH), 121.5 (CH), 118.9 (C), 118.7 (CH), 118.2 (2 x CH), 111.2 (C); HRMS (TOF MS ES$^-$) *m/z* calculated for $C_{13}H_9N_2SO_2$ 257.0385 $[M\text{-}H]^+$, found 257.0390. Data are consistent with literature values.[9]

Compound 1p

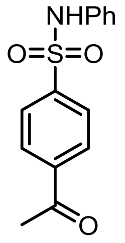

Chemical Formula: $C_{14}H_{13}NO_3S$
MW = 275

4-Acetyl-*N*-phenylbenzenesulfonamide was synthesised according to general procedure A to afford a pale brown solid (246 mg, 89%). R_f 0.04 (EtOAc:hexanes 1:9); ^{1}H NMR (400 MHz, $CDCl_3$) δ 8.07 (d, *J* = 8.4 Hz, 2H, ArH), 7.95 (d, *J* = 8.5 Hz, 2H, ArH), 7.38 – 7.29 (m, 3H, ArH), 7.23 (t, *J* = 7.4 Hz, 2H, ArH), 7.18 (d, *J* = 7.6 Hz, 2H, ArH), 2.70 (s, 3H, CH_3); ^{13}C NMR (101 MHz, $CDCl_3$) δ 197.2 (C=O), 142.8 (C), 140.2 (C), 136.0 (C), 129.6 (2 x CH), 129.0 (2 x CH), 127.7 (2 x CH), 126.0 (CH), 122.1 (2 x CH), 27.04 (CH_3). Data are consistent with literature values.[10]

Compound 1q

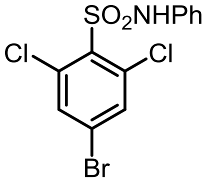

Chemical Formula: $C_{12}H_8BrCl_2NO_2S$
MW = 381

4-Bromo-2,6-dichloro-*N*-phenylbenzenesulfonamide was synthesized according to general procedure A to give an white crystalline solid (248 mg, 70%). R_f 0.55 (EtOAc: hexane 1:4); ^{1}H NMR (400 MHz, DMSO-d$_6$) δ 10.90 (s, 1H, NH), 7.96 (s, 2H, ArH), 7.28 – 7.24 (m, 2H, ArH), 7.10 –7.08 (m, 2H, ArH), 7.06 – 7.02 (m 1H, ArH); ^{13}C NMR (101 MHz, DMSO-d$_6$) δ 136.6 (C), 135.3 (2 x C), 134.1 (2 x CH), 133.4 (C), 129.4 (2 x CH), 126.6 (CH), 124.2 (CH), 118.8 (2 x CH); HRMS (TOF MS ES$^-$) *m/z* calculated for $C_{12}H_7NO_2SCl_2Br$ 377.8758 [*M*-H$^+$], found 377.8765; Mp: 108 °C.

Compound 1r

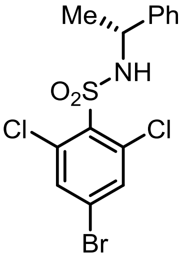

Chemical Formula: $C_{14}H_{12}BrCl_2NO_2S$
Molecular Weight: 409

(R)-4-bromo-2,6-dichloro-*N*-(1-phenylethyl)benzenesulfonamide **1q** was synthesised according to general procedure B (4 mmol) to give a white crystalline solid (1.26g, 78%). ^{1}H NMR (500 MHz, $CDCl_3$) δ 7.36 (s, 2H, ArH), 7.15 – 7.07 (m, 5H, ArH), 5.78 (d, *J* = 8.5 Hz, 1H, CH), 4.66 – 4.55 (m, 1H, NH), 1.52 (d, *J* = 7.0 Hz, 3H, CH_3); ^{13}C NMR (126 MHz, $CDCl_3$) δ 140.4, 135.3, 135.2, 133.6, 128.5, 127.8, 125.9, 125.5, 54.7, 22.9.

Compound 1s

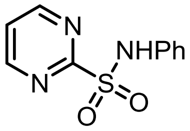

Chemical Formula: $C_{10}H_9N_3O_2S$
Molecular Weight: 235

N-Phenylpyrimidine-2-sulfonamide was synthesised according to a modified literature procedure.[11] HCl (2 M, 25 mL) and CH_2Cl_2 (25 mL) were cooled to – 5 °C. With rapid stirring cold NaOCl (10 %, 3.3 eq., 11 mL) was added at such a rate than the internal temperature does not exceed 0 °C. The 2-mercaptanpyrimidine (560 mg, 5 mmol) was added in small portions and an internal temperature of -5 to -10 °C was maintained. The reaction was left to stir rapidly for 20 minutes after which time the excess chlorine was quenched by adding $Na_2S_2O_3$ (sat.). The crude reaction was transferred to a cold separating funnel and the organic phase was quickly extracted with cold dichloromethane CH_2Cl_2 (50 mL). The organic phase was cooled to 0 °C and aniline (1.4 mL, 15 mmol, 3 eq.) was added. The ice bath was then removed and the reaction was stirred for 40 minutes until completion was confirmed by TLC (1:19 Et_2O:CH_2Cl_2). The crude reaction mixture was purified by column chromatography to afford a pale yellow solid (200 mg, 0.11 mmol, 17%). ^{1}H NMR (400 MHz, $CDCl_3$) δ 8.79 (t, *J* = 4.3 Hz, 2H, ArH), 8.11 (d, *J* = 38.5 Hz, 1H, NH), 7.40 (td, *J* = 4.9, 1.7 Hz, 1H, ArH), 7.21 (dd, *J* = 8.6, 1.4 Hz, 2H, ArH), 7.14 (ddd, *J* = 8.1, 6.6, 3.0 Hz, 2H, ArH), 7.01 (tq, *J* = 6.9, 1.7 Hz, 1H, ArH); ^{13}C NMR (126 MHz, $CDCl_3$) δ 164.8, 164.8, 158.6, 136.0, 129.3, 125.7, 123.6, 123.6, 122.1; *m/z* (ES^-) 234 ($[M\text{-}H]^-$).

Compound 1t

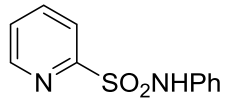

Chemical Formula: $C_{11}H_{10}N_2O_2S$
Molecular Weight: 234

N-Phenylpyridine-2-sulfonamide **1t** was synthesised according to a modified literature procedure.[11] HCl (2 M, 25 mL) and CH_2Cl_2 (25 mL) were cooled to – 5 °C. With rapid stirring cold NaOCl (10 %, 3.3 eq., 11 mL) was added at such a rate than the internal temperature does not exceed 0 °C. The 2-mercaptanpyridine (555 mg, 5 mmol) was added in small portions and internal temperature of -5 to -10 °C was maintained. The reaction was left to stir rapidly for 20 minutes after which time the excess chlorine was quenched by adding $Na_2S_2O_3$ (sat.). The crude reaction was transferred to a cold separating funnel and the organic phase was quickly extracted with cold dichloromethane CH_2Cl_2 (50 mL). The organic phase was cooled to 0 °C and aniline (1.4 mL, 15 mmol, 3 eq.) was added. The ice bath was then removed and the reaction was stirred for 40 minutes until completion was confirmed by TLC (1:19 Et_2O:CH_2Cl_2). The crude reaction mixture was purified by column chromatography to afford a pale yellow solid (820 mg, 3.5 mmol, 70%). ^{1}H NMR (400 MHz, Acetone-d_6) δ 9.22

(s, 1H, NH), 8.65 (ddd, J = 4.7, 1.7, 0.9 Hz, 1H, ArH), 8.01 – 7.89 (m, 2H, ArH), 7.55 (ddd, J = 7.4, 4.7, 1.4 Hz, 1H, ArH), 7.28 – 7.13 (m, 4H, ArH), 7.03 – 6.97 (m, 1H, ArH); ^{13}C NMR (101 MHz, Acetone-d_6) δ 157.9 (C), 150.9 (CH), 139.1 (CH), 138.6 ©, 129.8, 127.9, 125.2, 123.5, 121.8; ES$^-$ 233 ([M-H]$^-$, 100%).

Compound 1u

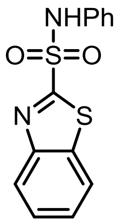

Chemical Formula: $C_{13}H_{10}N_2O_2S_2$
MW = 290

N-Phenylbenzo[d]thiazole-2-sulfonamide **1u** was synthesised according to modified literature procedure.[12] A stirred suspension of 2-mercaptobenzothiazole (2.0 g, 12 mmol, 1 eq.) in HCl (1M, 30 mL) and CH_2Cl_2 (0.2 M, 60 mL) was cooled in a salt ice bath and sodium hypochlorite (~10%, 36 mL, 0.36 mmol, 0.03 eq.) was slowly added. The solution continued to be stirred for 1 hour. The reaction mixture was then separated in a pre-cooled separating funnel, and the aqueous layer was extracted with cold CH_2Cl_2 (10 mL). The organic layers were quickly washed with cold $NaHCO_3$ and saturated brine, and dried over $MgSO_4$ for 30 minutes at -78 °C under a nitrogen atmosphere, then filtered and concentrated under vacuum. Ice cold dry diethyl ether was added (5 mL) and the mixture cooled to -78 °C, filtered and then the solid washed with cold (-78 °C) dry Et_2O and dried for 30 minutes. The resulting cream solid was stored under nitrogen at -18 °C.

A portion of the cream solid (500 mg, 2.1 mmol) was then dissolved in ethanol at 0 °C and aniline (0.4 mL, 4.2 mmol) was added. The mixture was warmed to room temperature by which time the reaction appeared complete by TLC. The crude reaction mixture was recrystallized from ethanol, however some aniline remained, and so column chromatography was performed (SiO_2 gel, 0:1 to 1:1 EtOAc:hexane) to afford a white crystalline solid (207 mg, 35%). ^{1}H NMR (400 MHz, DMSO-d_6) δ 11.24 (s, 1H), 8.28 – 8.21 (m, 1H), 8.18 (d, J = 7.7 Hz, 1H), 7.71 – 7.58 (m, 3H), 7.28 (d, J = 7.7 Hz, 2H), 7.21 (d, J = 7.5 Hz, 2H), 7.11 (t, J = 7.3 Hz, 1H); ^{13}C NMR (101 MHz, DMSO-d_6) δ 165.6 (C), 151.6 (C), 136.4 (C), 135.9 (C), 129.3 (2 x CH), 127.9 (CH), 127.8 (CH), 125.1 (CH), 124.6 (CH), 123.3 (CH), 121.1 (2 x

CH); HRMS (TOF MS ES^+) *m/z* calculated for 313.0081 $[M+Na]^+$, found 313.0089. Data are consistent with literature values.[13]

4. General Procedures for Synthesis of Biaryls

General Procedure D

The sulfonamide (1 eq.), potassium fluoride (3 eq.) and 18-crown-6 (3 eq.) were measured into a microwave vial and then THF (0.1M) and the aryne precursor (1 eq.) were added. The vial was sealed with a cap and the solution was then stirred at reflux for 24 hours. The reaction was then cooled, and then diluted with ethyl acetate and water. The aqueous phase was separated and extracted twice with ethyl acetate. The combined organic were washed with brine, dried over anhydrous magnesium sulphate, filtered and concentrated under vacuum. The crude product was then purified using column chromatography (SiO_2 gel, 0:1 to 1:9 *v/v* EtOAc:hexanes) to afford the title compound.

General Procedure E

The sulfonamide (1 eq.), potassium fluoride (6 eq.) and 18-crown-6 (6 eq.) were measured into a microwave vial and then THF (0.1M) and the aryne precursor (2 eq.) were added. The vial was sealed with a cap and the solution was then stirred at reflux for 24 hours. The reaction was then cooled, and then diluted with ethyl acetate and water. The aqueous phase was separated and extracted twice with ethyl acetate. The combined organic were washed with brine, dried over anhydrous magnesium sulphate, filtered and concentrated under vacuum. The crude product was then purified using column chromatography (SiO_2 gel, 0:1 to 1:9 *v/v* EtOAc:hexanes) to afford the title compound.

Characterisation data for biaryls

Nitro-phenyl sulfonamide scope (Scheme 2)

Compound 4a

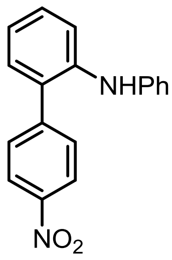

Chemical Formula: $C_{18}H_{14}N_2O_2$
MW = 290

4'-Nitro-*N*-phenyl-[1,1'-biphenyl]-2-amine **4a** was synthesised according to general procedure D (0.25 mmol). The title compound was isolated as a red crystalline solid (40 mg, 56%). R_f 0.83 (EtOAc:hexane, 1:9); ^{1}H NMR (400 MHz, $CDCl_3$) δ 8.26 (d, *J* = 8.8 Hz, 2H, ArH), 7.65 (d, *J* = 8.8 Hz, 2H, ArH), 7.40 (d, *J* = 8.1 Hz, 1H, ArH), 7.36 – 7.30 (td, J = 8.3, 1.1 Hz, 1H, ArH), 7.30 – 7.21 (m, 3H, ArH), 7.07 (td, *J* = 7.4, 1.3 Hz, 1H, ArH), 7.00 (d, J = 7.7 Hz, 2H, ArH), 6.95 (tt, *J* = 7.4, 1.1 Hz, 1H, ArH), 5.44 (s, 1H, NH); ^{13}C NMR (101 MHz, $CDCl_3$) δ 147.1 (C), 146.3 (C), 143.1 (C), 140.3 (C), 130.9 (CH), 130.3 (2 x CH), 129.8 (C), 129.7 (CH), 129.6 (2 x CH), 124.2 (2 x CH), 122.1 (CH), 121.6 (CH), 119.0 (2 x CH), 118.3 (CH); HRMS (TOF MS ES^+) *m/z* calculated for $C_{18}H_{13}N_2O_2$ 289.0977 [*M*-H]$^-$, found 289.0991; mp: 93-98 °C.

Compound 4b

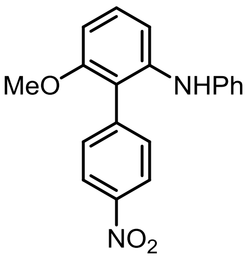

Chemical Formula: $C_{19}H_{16}N_2O_3$
MW = 320

6-Methoxy-4'-nitro-*N*-phenyl-[1,1'-biphenyl]-2-amine **4b** was synthesised according to general procedure D (0.25 mmol) and was isolated as an orange crystalline solid (52 mg, 65%). R_f 0.39 (EtOAc:hexanes, 1:9); ^{1}H NMR (400 MHz, $CDCl_3$) δ 8.28 (d, *J* = 8.6 Hz, 2H, ArH), 7.54 (d, *J* = 8.6 Hz, 2H, ArH), 7.31 – 7.19 (m, 3H, ArH), 7.0 (t, *J* = 7.9 Hz, 3H, ArH),

6.95 (t, J = 7.3 Hz, 1H, ArH), 6.60 (dd, J = 8.3, 0.9 Hz, 1H, ArH), 5.16 (br s, 1H, ArH), 3.74 (s, 3H, ArH); ^{13}C NMR (101 MHz, $CDCl_3$) δ 157.5 (C), 147.2 (C), 142.7 (C), 142.3 (C), 141.9 (C), 132.2 (2 x CH), 129.9 (CH), 129.5 (2 x CH), 124.0 (2 x CH), 121.9 (CH), 119.1 (2 x CH), 117.8 (C), 110.4 (CH), 103.6 (CH), 55.8 (OCH_3); HRMS (TOF MS ES$^+$) *m/z* calculated for $C_{19}H_{16}N_2O_3Na$ 343.1059 [*M*+Na]$^+$, found 343.1075; mp 153 - 156 °C.

Compound 4c

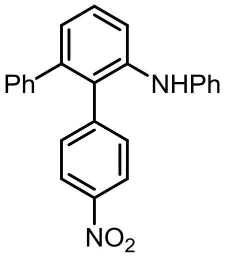

Chemical Formula: $C_{24}H_{18}N_2O_{32}$
MW = 366

4''-Nitro-*N*-phenyl-[1,1':2',1''-terphenyl]-3'-amine **4c** was synthesised according to general procedure D (0.25 mmol) and isolated as an orange solid (65 mg, 71%). R_f 0.41 (EtOAc:hexanes, 1:9); ^{1}H NMR (500 MHz, $CDCl_3$) δ 8.07 (d, J = 8.8 Hz, 2H, ArH), 7.38 (dd, J = 8.2, 1.3 Hz, 1H, ArH), 7.34 (d, J = 7.4 Hz, 1H, ArH), 7.30 (dt, J = 9.1, 2.5 Hz, 2H, ArH), 7.25 (dd, J = 8.5, 7.4 Hz, 2H, ArH), 7.14 (dt, J = 4.1, 1.6 Hz, 2H, ArH), 7.04 – 6.98 (m, 4H, ArH), 6.95 (t, J = 7.4 Hz, 1H, ArH), 5.17 (s, 1H, NH); ^{13}C NMR (126 MHz, $CDCl_3$) δ 146.9 (C), 144.9 (C), 142.9 (C), 142.8 (C), 140.9 (C), 132.3 (2 x CH), 129.7 (2 x CH), 129.6 (2 x CH), 129.2 (CH), 128.0 (2 x CH), 127.9 (C), 126.9 (CH), 123.8 (2 x CH), 123.3 (CH), 121.9 (CH), 119.0 (2 x CH), 116.7 (C); HRMS (TOF MS APCI$^+$) *m/z* calculated for $C_{24}H_{19}N_2O_2$ 367.1447 [*M*+H]$^+$, found 367.1429; Mp 148 °C.

Compounds 4d

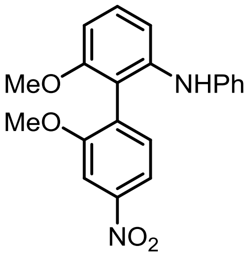

Chemical Formula: $C_{20}H_{18}N_2O_4$
MW = 350

2',6-Dimethoxy-4'-nitro-N-phenyl-[1,1'-biphenyl]-2-amine **4d** was synthesised according to general procedure D (0.46 mmol) and was isolated as red crystalline solid (99 mg, 62%). R_f 0.2 (EtOAc:hexanes 1:9); ^{1}H NMR (400 MHz, $CDCl_3$) δ 7.83 (d, *J* = 8.3 Hz, 1H, ArH), 7.75 (s, 1H, ArH), 7.33 (d, *J* = 8.3 Hz, 1H, ArH), 7.21 – 7.11 (m, 3H, ArH), 6.94 (d, *J* = 8.2 Hz, 1H, ArH), 6.88 (d, *J* = 7.9 Hz, 2H, ArH), 6.82 (t, *J* = 7.4 Hz, 1H, ArH), 6.54 (d, *J* = 8.2 Hz, 1H, ArH), 5.16 (brs, 1H, NH), 3.72 (s, 3H, OCH_3), 3.65 (s, 3H, OCH_3); ^{13}C NMR (100 MHz, $CDCl_3$) δ 157.8 (C), 157.6 (C), 148.4 (C), 143.1 (C), 142.1 (C), 133.2 (CH), 131.0 (C) , 129.5 (CH), 129.2 (2 x CH), 121.2 (CH), 118.4 (2 x CH), 116.0 (CH), 114.9 (C), 110.7 (CH), 106.2 (C), 103.7 (CH), 56.1 (OCH_3), 55.7 (OCH_3); HRMS (TOF MS ES^+) *m/z* calculated for $C_{20}H_{18}N_2O_4Na$ 373.1164 $[M+Na]^+$, found 373.1179; mp: 162°C; rt (HPLC) 16.17, 17.39.

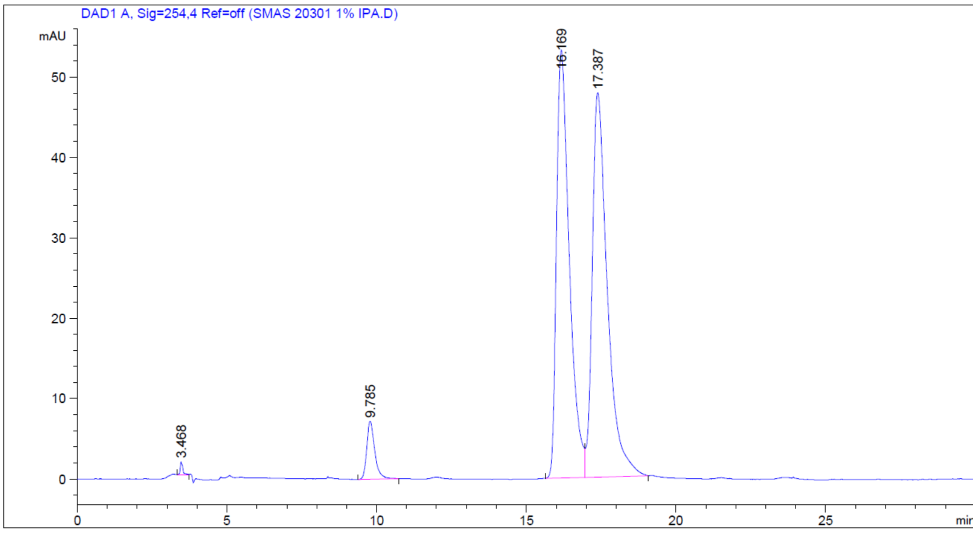

Compound 4e’

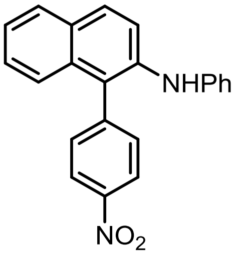

Chemical Formula: $C_{22}H_{16}N_2O_2$
MW = 340

1-(4-Nitrophenyl)-*N*-phenylnaphthalen-2-amine **4e’** was synthesised according to general procedure D (0.25 mmol) and was isolated as a red crystalline solid (43 mg, 51%). R_f 0.42 (EtOAc:hexanes, 1:9); ^{1}H NMR (400 MHz, $CDCl_3$) δ 8.43 – 8.36 (m, 2H, ArH), 7.86 – 7.79 (m, 2H, ArH), 7.63 – 7.55 (m, 3H, ArH), 7.36 (ddd, *J* = 6.7, 4.8, 3.3 Hz, 2H, ArH), 7.32 – 7.22 (m, 3H, ArH), 7.05 – 6.92 (m, 3H, ArH), 5.34 (br s, 1H, NH); ^{13}C NMR (101 MHz, $CDCl_3$) δ 147.7 (C), 144.4 (C), 143.1 (C), 138.1 (C), 133.1 (C), 132.4 (2 x CH), 129.7 (CH), 129.6 (2 x CH), 129.5 (C), 128.4 (CH), 127.1 (CH), 124.6 (2 x CH), 124.2 (CH), 124.0 (CH), 123.3 (C), 122.0 (CH), 119.4 (CH), 118.8 (2 x CH); *m/z* (EI^+) 340 ($[M]^+$, 100%); HRMS (TOF MS ES^+) *m/z* calculated for $C_{22}H_{16}O_2N_2$ 340.1206 $[M]^+$, found 340.1197; mp: 162-164 °C.

Compound 4e’’

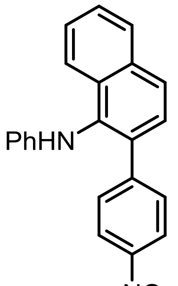

Chemical Formula: $C_{22}H_{16}N_2O_2$
MW = 340

2-(4-Nitrophenyl)-*N*-phenylnaphthalen-1-amine **4e’’** was synthesised according to general procedure D (0.25 mmol scale) and was isolated as a yellow solid (14 mg, 17%). R_f 0.3 (EtOAc:hexanes, 1:9); ^{1}H NMR (400 MHz, $CDCl_3$) δ 8.22 (d, *J* = 8.5 Hz, 2H, ArH), 8.02 (d, *J* = 8.4 Hz, 1H, ArH), 7.93 (d, *J* = 8.1 Hz, 1H, ArH), 7.84 (d, *J* = 8.5 Hz, 1H, ArH), 7.56 (dd, *J* = 9.2, 2.6 Hz, 3H, ArH), 7.54 – 7.43 (m, 2H, ArH), 7.15 (dd, *J* = 8.4, 7.1 Hz, 2H, ArH), 6.81 (t, *J* = 7.3 Hz, 1H, ArH), 6.61 – 6.49 (m, 2H, ArH), 5.52 (s, 1H, NH); ^{13}C NMR (101

MHz, $CDCl_3$) δ 147.3 (C), 147.2 (C), 146.8 (C), 134.8 (C), 134.7 (C), 132.6 (C), 130.7 (C), 130.2 (2 x CH), 129.5 (2 x CH), 128.5 (CH), 127.7 (CH), 127.0 (CH), 126.9 (CH), 126.5 (CH), 125.0 (CH), 123.9 (2 x CH), 119.7 (CH), 114.9 (2 x CH); HRMS (EI, +ve) *m/z* Calculated for $C_{22}H_{16}O_2N_2$ 340.1206 $[M]^+$, found 340.1214; mp: decomposition at 202 °C.

Compounds 4f

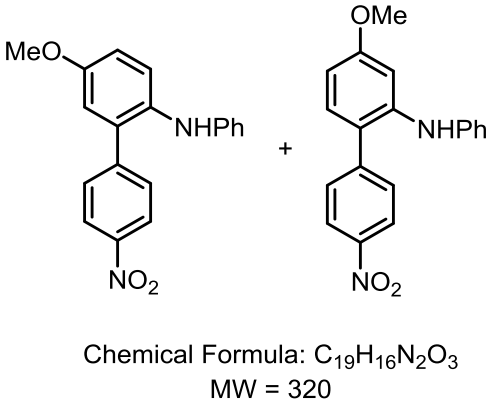

5-Methoxy-4'-nitro-*N*-phenyl-[1,1'-biphenyl]-2-amine and 4-methoxy-4'-nitro-*N*-phenyl-[1,1'-biphenyl]-2-amine **4f** were synthesised, as an inseparable mixture, according to general procedure D (0.48 mmol) and were isolated as a red solid (77 mg, 67%). R_f 0.25 (EtOAc: hexanes, 1:9); ^{1}H NMR (400 MHz, $CDCl_3$) δ ^{1}H NMR (400 MHz, $CDCl_3$) δ 8.31 – 8.18 (m, 4H, ArH), 7.70 – 7.52 (m, 4H, ArH), 7.36 (d, *J* = 8.8 Hz, 1H, ArH), 7.31 – 7.27 (m, 1H, ArH), 7.24 – 7.14 (m, 3H, ArH), 7.09 – 7.01 (m, 2H, ArH), 7.01 – 6.90 (m, 3H, ArH), 6.90 – 6.76 (m, 5H, ArH), 6.62 (dd, *J* = 8.5, 2.5 Hz, 1H, ArH), 5.47 (s, 1H, NH), 5.11 (s, 1H, NH), 3.85 (s, 3H, OCH_3), 3.80 (s, 3H, OCH_3); ^{13}C NMR (101 MHz, $CDCl_3$) δ 160.9 (C), 155.9 (C), 147.0 (C), 146.7 (C), 146.1 (C), 146.0 (C), 145.2 (C), 142.5 (C), 141.5 (C), 133.9 (C), 132.5 (C), 131.7 (C), 130.1 (C), 130.0 (2 x CH), 129.5 (2 x CH), 129.4 (2 x CH), 124.7 (CH), 124.1 (2 x CH), 123.8 (2 x CH), 122.0 (C), 121.8 (CH), 119.8 (C), 118.7 (2 x CH), 115.7 (CH), 115.5 (2 x CH), 115.2 (CH), 107.4 (CH), 103.6 (C), 55.7 (OCH_3), 55.4 (OCH_3); HRMS (TOF MS ES^+) calculated for $C_{19}H_{16}N_2O_3Na$ 343.1059 $[M+Na]^+$, found 343.1074.

Compounds 4g

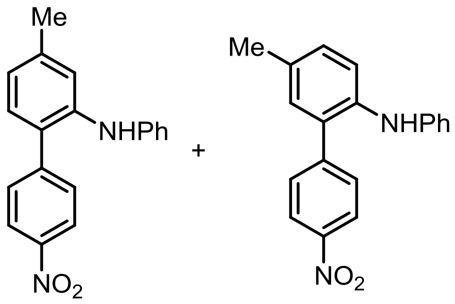

Chemical Formula: $C_{19}H_{16}N_2O_2$
MW = 304

4-Methyl-4'-nitro-*N*-phenyl-[1,1'-biphenyl]-2-amine and 5-methyl-4'-nitro-*N*-phenyl-[1,1'-biphenyl]-2-amine **4g** were synthesised, as an inseparable mixture, of isomers according to general procedure D (0.46 mmol) and were isolated as a red crystalline solid (89 mg, 64%). R_f 0.45 (EtOAc:hexanes 1:9); ^{1}H NMR (400 MHz, $CDCl_3$) δ 8.30 – 8.20 (m, 4H, ArH), 7.68 – 7.56 (m, 4H, ArH), 7.35 – 7.28 (m, 1H, ArH), 7.26 – 7.19 (m, 4H, ArH), 7.17 (dd, *J* = 8.1, 5.4 Hz, 2H, ArH), 7.11 (s, 1H, ArH), 6.99 (dt, *J* = 6.9, 1.2 Hz, 2H, ArH), 6.97 – 6.83 (m, 5H, ArH), 5.38 (s, 1H, NH), 5.28 (s, 1H, NH), 2.37 (s, 3H, CH_3), 2.35 (s, 3H, CH_3); ^{13}C NMR (101 MHz, $CDCl_3$) δ 146.9 (C), 146.8 (C), 146.3 (C), 146.2 (C), 143.8 (C), 143.0 (C), 139.9 (C), 139.9 (C), 137.2 (C), 132.1 (CH), 131.2 (CH), 130.7 (C), 130.6 (CH), 130.3 (CH), 130.1 (2 x CH), 130.1 (2 x CH), 129.4 (2 x CH), 129.4 (2 x CH), 127.0 (C), 124.0 (2 x CH), 123.9 (2 x CH), 122.9 (CH), 121.4 (CH), 120.7 (CH), 120.5 (CH), 119.4 (CH), 118.1 (2 x CH), 117.0 (2 x CH), 21.4 (CH_3), 20.7 (CH_3); HRMS (TOS MS ES^+) calculated for $C_{19}H_{17}N_2O_2$ 305.1290 $[M+H]^+$, found 305.1298.

Compounds 4h

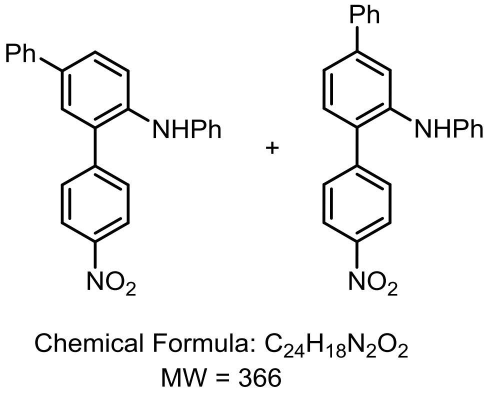

4''-Nitro-*N*-phenyl-[1,1':3',1''-terphenyl]-4'-amine and 4-nitro-*N*-phenyl-[1,1':4',1''-terphenyl]-2'-amine were synthesised **4h**, as an inseparable mixture of isomers, according to general procedure D (0.48 mmol) and were isolated as a red crystalline solid (111 mg, 66%). R_f 0.37 (EtOAc:hexanes 1:9); ^{1}H NMR (400 MHz, $CDCl_3$) δ 8.36 – 8.23 (m, 4H, ArH), 7.77 – 7.67 (m, 4H, ArH), 7.63 (d, *J* = 1.7 Hz, 1H, ArH), 7.62 – 7.53 (m, 5H, ArH), 7.52 – 7.42 (m, 6H, ArH), 7.41 – 7.33 (m, 3H, ArH), 7.33 – 7.21 (m, 9H, ArH), 7.06 (td, *J* = 5.6, 4.9, 2.5 Hz, 4H, ArH), 7.02 – 6.91 (m, 2H, ArH), 5.50 (s, 1H, NH), 5.47 (s, 1H, NH); ^{13}C NMR (101 MHz, $CDCl_3$) δ 147.1 (C), 147.0 (C), 146.0 (C), 145.8 (C), 142.8 (C), 142.7 (C), 142.6 (C), 140.5 (C), 140.3 (C), 140.1 (C), 139.5 (C), 134.7 (C), 131.2 (CH), 130.3 (2 x CH), 130.1 (2 x CH), 129.7 (C), 129.6 (2 x CH), 129.5 (2 x CH), 129.3 (C), 128.9 (2 x CH), 128.8 (2 x CH), 128.5 (CH), 128.2 (CH), 127.7 (CH), 127.1 (2 x CH), 126.6 (2 x CH), 124.2 (2 x CH), 124.2 (2 x CH), 121.7 (CH), 121.6 (CH), 120.7 (CH), 118.7 (CH), 118.5 (2 x CH), 118.2 (2 x CH), 117.3 (CH); HRMS (TOF MS ES^-) calculated for $C_{24}H_{17}N_2O_2$ 365.1290 $[M\text{-}H]^-$, found 365.1273.

Compounds 4i

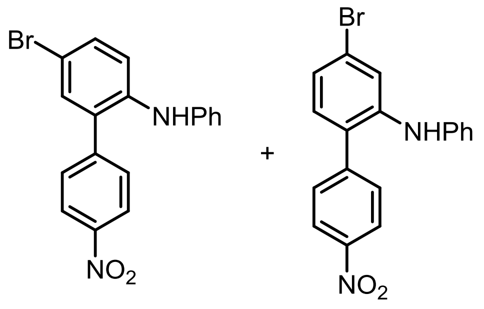

Chemical Formula: $C_{18}H_{13}BrN_2O_2$
MW = 369

5-Bromo-4'-nitro-*N*-phenyl-[1,1'-biphenyl]-2-amine and 4-bromo-4'-nitro-*N*-phenyl-[1,1'-biphenyl]-2-amine **4i** were synthesised, as an inseparable mixture, according to general procedure D (0.25 mmol) and were isolated as red solid (38 mg, 63%). R_f 0.43 (EtOAc:hexanes 1:9); ^{1}H NMR (400 MHz, $CDCl_3$) δ ppm 8.28 (d, *J* = 8.1 Hz, 2 H, ArH) 7.58 - 7.69 (m, 2 H, ArH) 7.36 - 7.53 (m, 2 H, ArH) 7.21 - 7.35 (m, 3 H, ArH) 6.94 - 7.19 (m, 4 H, ArH) 5.49 (s, 1 H, NH) 5.41 (s, 1 H, NH); ^{13}C NMR (101 MHz, $CDCl_3$) δ 147.2, 147.1, 145.0, 144.6, 142.1, 141.9, 141.4, 139.5, 133.1, 132.3, 131.9, 130.9, 130.1, 130.1, 129.6, 129.5, 127.3, 124.3, 124.2, 124.1, 123.6, 122.7, 122.1, 119.7, 119.7, 119.5, 118.6, 113.5; *m/z* (TOF MS AP$^+$) 369 ([*M*+H]$^+$, 100%), 371 ([*M*+H]$^+$, 100%); HRMS (TOF MS AP$^+$) *m/z* calculated for $C_{18}H_{14}N_2O_2Br$ 369.0239 [*M*+H]$^+$ found 369.0251.

Compound 4j

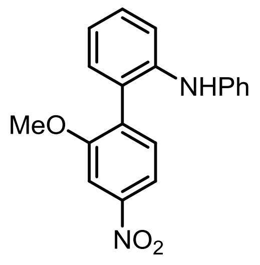

Chemical Formula: $C_{19}H_{16}N_2O_3$
MW = 320

2'-Methoxy-4'-nitro-*N*-phenyl-[1,1'-biphenyl]-2-amine **4j** was synthesised according to general procedure D (0.46 mmol) and was isolated as bright red solid (120 mg, 82%). R_f0.3 (EtOAc:hexanes, 1:9); ^{1}H NMR (400 MHz, $CDCl_3$) δ 7.93 (dd, *J* = 8.3, 2.0 Hz, 1H, ArH), 7.83 (d, *J* = 2.0 Hz, 1H, ArH), 7.45 (d, *J* = 8.3 Hz, 1H, ArH), 7.40 (dd, *J* = 8.3 , 1.3 Hz, 1H, ArH), 7.35 – 7.31 (m, 1H, ArH), 7.23 – 7.20 (m, 3H, ArH), 7.06 (dt, *J* = 7.4, 1.5 Hz, 1H, ArH), 6.96 – 6.93 (m, 2H, ArH), 6.90 – 6.87 (m, 1H, ArH), 5.54 (brs, 1H, NH), 3.85 (s, 3H,

OCH_3); ^{13}C NMR (101 MHz, $CDCl_3$) δ 156.9 (C), 148.3 (C), 143.4 (C), 141.0 (C), 135.3 (C), 132.2 (CH), 131.2 (CH), 129.3 (C), 129.2 (2 x CH), 127.4 (C), 121.5 (CH), 120.8 (CH), 118.8 (CH), 117.6 (2 x CH), 116.3 (CH), 105.9 (CH), 56.1 (OCH_3); HRMS (TOF MS ES^+) *m/z* calculated for $C_{19}H_{16}N_2O_3Na$ 343.1059 [*M*+Na]$^+$, found 343.1071; mp: 82 °C.

Compound 4k

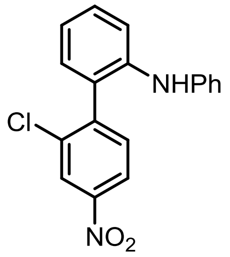

Chemical Formula: $C_{18}H_{13}ClN_2O_2$
MW = 325

2'-Chloro-4'-nitro-*N*-phenyl-[1,1'-biphenyl]-2-amine **4j** was synthesised according to general procedure D (0.46 mmol) and was isolated as red crystalline solid (86 mg, 58%). R_f 0.3 (EtOAc:hexanes, 1:9); ^1H NMR (400 MHz, $CDCl_3$) δ 8.28 (d, *J* = 2.3 Hz, 1H, ArH), 8.09 (dd, *J* = 8.4, 2.3 Hz, 1H, ArH), 7.48 (d, *J* = 8.4 Hz, 1H, ArH), 7.32 – 7.25 (m, 2H, ArH), 7.17 – 7.13 (m, 2H, ArH), 7.10 – 7.28 (m, 1H, ArH), 7.00 – 6.96 (m, 1H, ArH), 6.91 – 6.88 (m, 2H, ArH), 6.87 – 6.83 (m, 1H, ArH), 5.10 (brs, 1H, NH); ^{13}C NMR (100 MHz, $CDCl_3$) δ 147.7 (C), 144.8 (C), 142.6 (C), 140.7 (C), 135.2 (C), 132.7 (CH), 130.4 (CH), 129.9 (CH), 129.3 (2 x CH), 127.4 (C), 125.1 (CH), 122.1 (CH), 121.7 (CH), 121.3 (CH), 118.6 (2 x CH), 118.2 (CH); HRMS (TOF MS AP^+) *m/z* calculated for $C_{18}H_{14}N_2O_2Cl$ 325.0744 [*M*+H]$^+$, found 325.0755; mp: 102 °C.

Compound 4l

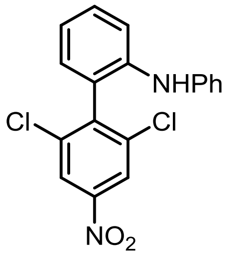

Chemical Formula: $C_{18}H_{12}Cl_2N_2O_2$
MW = 358

2',6'-Dichloro-4'-nitro-*N*-phenyl-[1,1'-biphenyl]-2-amine **4l** was synthesised according to general procedure D (0.25 mmol) and was isolated as a deep red crystalline solid (72 mg,

81%). R_f 0.56 (EtOAc:hexanes 1:9); ^{1}H NMR (400 MHz, DMSO-d_6) δ 8.37 (s, 2H, ArH), 7.34 – 7.26 (m, 2H, ArH), 7.21 (brs, 1H, NH), 7.13 (t, *J* = 7.8 Hz, 2H, ArH), 7.09 – 7.06 (m, 1H, ArH), 7.02 – 6.97 (m, 1H, ArH), 6.95 (d, *J* = 7.7 Hz, 2H, ArH), 6.76 (t, *J* = 7.3 Hz, 1H, ArH); ^{13}C NMR (101 MHz, DMSO-d6) δ 147.3 (C), 143.7 (C), 143.6 (C), 141.5 (C), 136.5 (2 x CH), 130.4 (C), 130.0 (C), 129.0 (2 x CH), 125.4 (CH), 123.4 (2 x CH), 120.8 (CH), 120.3 (CH), 118.5 (CH), 117.9 (2 x CH); HRMS (TOF MS AP$^+$) *m/z* calculated for $C_{18}H_{13}N_2O_2Cl_2$ 359.0354 [*M*+H]$^+$, found 359.0346; mp 172-174 °C.

Compounds 4m

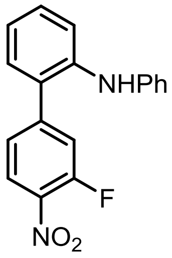

Chemical Formula: $C_{18}H_{13}FN_2O_2$
MW = 308

3'-Fluoro-4'-nitro-*N*-phenyl-[1,1'-biphenyl]-2-amine **4m** was synthesised according to general procedure D (0.46 mmol) and was isolated as red crystalline (58 mg, 41%). R_f 0.26 (EtOAc:hexanes, 1:9); ^{1}H NMR (400 MHz, $CDCl_3$) δ 8.0 (t, *J* = 8.3 Hz, 1H, ArH), 7.34 – 7.28 (m, 2H, ArH), 7.22 (t, *J* = 7.8 Hz, 1H, ArH), 7.17 – 7.13 (m, 4H, ArH), 6.95 (t, *J* = 7.4 Hz, 1H, ArH), 6.89 – 6.83 (m, 3H, ArH), 5.30 (brs, 1H, NH); ^{13}C NMR (126 MHz, $CDCl_3$) δ 155.9 (d, *J* = 265.7 Hz, CF), 147.9 (d, *J* = 8.5 Hz, C), 142.8 (C), 140.4 (C), 136.2 (d, *J* = 7.5 Hz, C), 130.8 (CH), 130.3 (CH), 129.6 (2 x CH), 128.7 (d, *J* = 1.5 Hz, C), 126.7 (d, *J* = 2.3 Hz, CH), 125.5 (d, *J* = 3.8 Hz, CH), 122.2 (CH), 121.9 (CH), 119.3 (C), 119.2 (CH), 119.1 (C), 118.5 (2 x CH); ^{19}F NMR (500 MHz, $CDCl_3$) δ -116.97; HRMS (TOF MS AP$^+$) *m/z* calculated for $C_{18}H_{14}N_2O_2F$ 309.1039 [*M*+H]$^+$, found 309.1037; mp: 62 °C.

Compound 4n

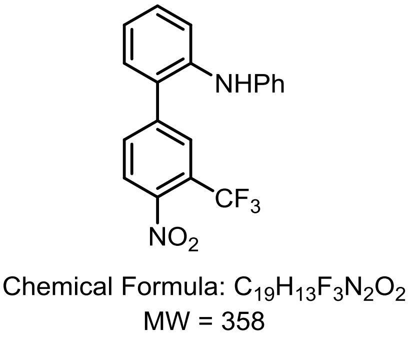

4'-Nitro-*N*-phenyl-3'-(trifluoromethyl)-[1,1'-biphenyl]-2-amine **4n** was synthesised according to general procedure D (0.46 mmol) and was isolated as yellow solid (44 mg, 27%). R_f 0.27 (EtOAc:hexanes, 1:9); ^{1}H NMR (400 MHz, $CDCl_3$) δ 7.87 – 7.85 (m, 2H, ArH), 7.77 (dd, *J* = 8.3, 1.8 Hz, 1H, ArH), 7.33 – 7.27 (m, 2H, ArH), 7.21 – 7.16 (m, 3H, ArH), 7.06 – 7.02 (m, 1H, ArH), 6.90 – 6.86 (m, 3H, ArH), 5.26 (brs, 1H, NH); ^{13}C NMR (101 MHz, $CDCl_3$) δ 146.7 (C), 146.6 (C), 142.6 (C), 140.2 (C), 133.6 (C), 130.7 (CH), 130.3 (C), 129.5 (2 x CH), 128.8 (q, *J* = 5.2 Hz, **CH**=C-CF_3), 128.6 (C), 125.6 (CH), 124.4 (q, *J* = 34.0 Hz, **C**-CF_3), 121.8 (q, *J* = 274.0 Hz, CF_3), 122.4 (CH), 121.7 (C), 119.5 (CH), 118.2 (2 x CH); ^{19}F NMR (400 MHz, $CDCl_3$) δ -59.93; HRMS (TOF MS AP^+) *m/z* calculated for $C_{19}H_{14}N_2O_2F_3$ 359.1007 $[M+H]^+$, found 359.0999; mp: 56 °C.

Compound 4o

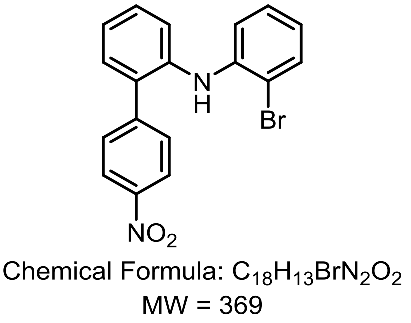

N-(2-Bromophenyl)-4'-nitro-[1,1'-biphenyl]-2-amine **4o** was synthesised according to general procedure D (0.25 mmol) was isolated as a yellow crystalline solid (68 mg, 73%). R_f 0.47 (EtOAc:hexanes, 1:9); ^{1}H NMR (500 MHz, $CDCl_3$) δ 8.30 – 8.21 (m, 2H, ArH), 7.65 – 7.59 (m, 2H, ArH), 7.51 – 7.45 (m, 1H, ArH), 7.41 – 7.36 (m, 2H, ArH), 7.34 (dt, *J* = 7.4, 1.1 Hz, 1H, ArH), 7.21 – 7.16 (m, 1H, ArH), 7.16 – 7.10 (m, 2H, ArH), 6.76 – 6.72 (m, 1H, ArH), 5.90 (s, 1H, NH); ^{13}C NMR (126 MHz, $CDCl_3$) δ 147.3 (C), 146.0 (C), 141.2 (C), 138.9 (C), 133.2 (CH), 132.0 (C), 131.0 (CH), 130.1 (2 x CH), 129.9 (CH), 128.3 (CH), 124.1 (2 x CH),

123.7 (CH), 121.6 (CH), 121.5 (CH), 116.3 (CH), 112.8 (C); HRMS (TOF MS AP$^+$) *m/z* calculated for $C_{18}H_{14}N_2O_2Br$ 369.0239 [*M*+H]$^+$, found 369.0241; mp 109-112 °C.

Compound 4p

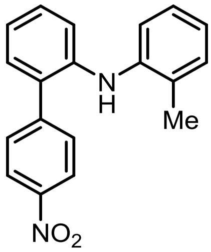

Chemical Formula: $C_{19}H_{16}N_2O_2$
MW = 304

4'-Nitro-*N*-(o-tolyl)-[1,1'-biphenyl]-2-amine **4p** was synthesised according to general procedure D (0.54 mmol) and was isolated as an orange crystalline solid (87 mg, 62%). R_f 0.38 (EtOAc:hexanes, 1:9); ^{1}H NMR (400 MHz, CD_2Cl_2) δ 8.26 (d, *J* = 8.7, 2H, ArH), 7.70 (d, *J* = 8.7, 2H, ArH), 7.31– 7.27 (m, 2H, ArH), 7.18 (d, *J* = 7.50, 1H, ArH), 7.12 – 7.11 (m, 2H, ArH), 7.06 – 7.01 (m, 2H, ArH) 6.95 – 6.92 (m, 1H, ArH), 5.33 (brs, 1H, NH), 2.12 (s, 3H, CH_3); ^{13}C NMR (101 MHz, CD_2Cl_2) δ147.4 (C), 146.8 (C), 141.6 (C), 141.4 (C), 131.3 (CH), 131.0 (CH), 130.6 (2 x CH), 130.0 (CH), 129.6 (C), 129.4 (C), 127.2 (CH), 124.4 (2 x CH), 122.9 (CH), 121.5 (CH), 119.9 (CH), 118.4 (CH), 18.0 (CH_3); HRMS (ES$^+$) calculated for $C_{19}H_{17}N_2O_2$ 305.1290 [*M*+H]$^+$, found 305.1279; mp: 120 °C.

Compound 4q

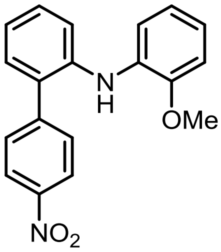

Chemical Formula: $C_{19}H_{16}N_2O_3$
MW = 320

N-(2-Methoxyphenyl)-4'-nitro-[1,1'-biphenyl]-2-amine **4q** was synthesised according to general procedure D (0.23 mmol) and as isolated as a dark yellow solid (71 mg, 49%). R_f0.25 (EtOAc:hexanes, 1:9); ^{1}H NMR (400 MHz, $CDCl_3$) δ 8.17 (d, *J* = 8.7, 2H, ArH), 7.56 (d, *J* = 8.7, 2H, ArH), 7.38 (d, *J* = 8.3, 1H, ArH), 7.27– 7.23 (m, 1H, ArH), 7.20 (dd, *J* = 7.7, 1.6 Hz, 1H, ArH), 7.17 – 7.13 (m, 1H, ArH), 6.99 (dt, *J* = 7.5, 1.2 Hz, 1H, ArH), 6.80 – 6.70 (m, 3H, ArH), 5.79 (brs, 1H, NH), 3.68 (s, 3H, OCH_3); ^{13}C NMR (101 MHz, $CDCl_3$) δ 148.6 (C),

146.9 (C), 146.3 (C), 139.8 (C), 132.6 (C), 130.7 (CH), 130.5 (C), 130.1 (2 x CH), 129.5 (CH), 123.9 (2 x CH), 122.0 (CH), 120.7 (CH), 120.5 (CH), 119.4 (CH), 115.2 (CH), 110.6 (CH), 55.5 (OCH_3); HRMS (ES^+) calculated for $C_{19}H_{17}N_2O_3$ 321.1239 $[M+H]^+$, found 321.1255; mp: 86 °C.

Compound 4r

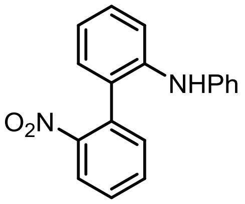

Chemical Formula: $C_{18}H_{14}N_2O_2$
MW = 290

2'-Nitro-*N*-phenyl-[1,1'-biphenyl]-2-amine **4r** was synthesised according to general procedure E from sulfonamide (0.33 mmol) and 2-trimethylsilylphenyl triflate (0.66 mmol) and was as isolated as a dark yellow oil (41 mg, 31%). R_f 0.39 (EtOAc:hexanes 2:8); ^{1}H NMR (400 MHz, $CDCl_3$) δ 8.11 (d, *J* = 8.1, 1H, ArH), 7.81 (t, *J* = 7.6 Hz, 1H, ArH), 7.68 (t, *J* = 7.8 Hz, 1H, ArH), 7.62 (d, *J* = 7.7 Hz, 1H, ArH), 7.52 – 7.47 (m, 2H, ArH), 7.39 (t, *J* = 7.8 Hz, 2H, ArH), 7.34 (d, *J* = 7.6 Hz, 1H, ArH), 7.24 – 7.21(m, 1H, ArH), 7.10– 7.06 (m, 3H, ArH), 5.37 (brs, 1H, NH); ^{13}C NMR (101 MHz, $CDCl_3$) δ 149.5 (C), 143.1 (C), 140.8 (C), 133.7 (C), 133.0 (CH), 132.7 (CH), 129.6 (CH), 129.3 (2 x CH), 129.2 (C), 128.7 (2 x CH), 124.3 (CH), 122.0 (CH), 121.2 (CH), 118.9 (CH), 118.1 (2 x CH); HRMS (ES^+) calculated for $C_{18}H_{15}N_2O_2$ 291.1134 $[M+H]^+$, found 291.1125.

Compound 7a

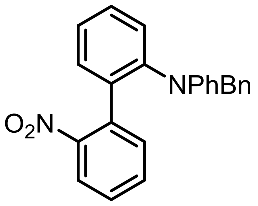

Chemical formula: $C_{25}H_{20}N_2O_2$
MW = 380

N-Benzyl-2'-nitro-*N*-phenyl-[1,1'-biphenyl]-2-amine **7a** was synthesised according to general procedure E (0.4 mmol) and was isolated as a red brown crystalline solid (109 mg, 72%). R_f 0.39 (EtOAc:hexanes, 1:9); ^{1}H NMR (500 MHz, $CDCl_3$) δ 7.78 (dd, J = 8.1, 1.3 Hz, 1H, ArH), 7.44 – 7.36 (m, 3H, ArH), 7.33 – 7.27 (m, 4H, ArH), 7.19 – 7.13 (m, 3H, ArH),

7.08 – 7.06 (m, 2H, ArH), 7.02 – 6.99 (m, 2H, ArH), 6.66 (tt, J = 7.3, 1.1 Hz, 1H, ArH), 6.61 – 6.59 (m, 2H, ArH), 4.64 (d, *J* = 17.2 Hz, 1H, C$\mathbf{H_a}$$H_b$), 4.55 (d, J = 17.2 Hz, 1H, C$H_a$$\mathbf{H_b}$); ^{13}C NMR (126 MHz, $CDCl_3$) δ 148.7 (C), 147.7 (C), 145.5 (C), 138.7 (C), 135.6 (C), 134.6 (C), 132.5 (CH), 132.3 (CH), 130.5 (CH), 129.8 (CH), 128.6 (2 x CH), 128.4 (CH), 128.3 (2 x CH), 128.1 (CH), 126.6 (2 x CH), 126.4 (CH), 126.0 (CH), 124.2 (CH), 118.5 (CH), 115.7 (2 x CH), 55.0 (CH_2); HRMS (ES^+) calculated for $C_{25}H_{20}N_2O_2Na$ 403.1422 [*M*+Na]$^+$, found 403.1419; mp: 96 °C.

Compound 7b

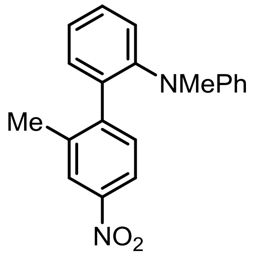

Chemical formula: $C_{20}H_{18}N_2O_2$
MW = 318

N,2'-Dimethyl-4'-nitro-*N*-phenyl-[1,1'-biphenyl]-2-amine **7b** was synthesised according to general procedure E (0.46 mmol) and was isolated as an amorphous yellow solid (77 mg, 53%). R_f 0.47 (EtOAc:hexanes, 1:9); ^{1}H NMR (400 MHz, $CDCl_3$) δ 7.95 (d, *J* = 2.3 Hz, 1H, ArH), 7.81 (dd, *J* = 8.5, 2.3 Hz, 1H, ArH), 7.36 – 7.32 (m, 1H, ArH), 7.27 (d, *J* = 8.0 Hz, 1H, ArH), 7.20 (dt, *J* = 7.4, 1.4 Hz, 1H, ArH), 7.17 – 7.14 (m, 1H, ArH), 7.11 (d, *J* = 8.5 Hz, 1H, ArH), 7.02 (t, *J* = 8.0 Hz, 2H, ArH), 6.64 (t, *J* = 7.4 Hz, 1H, ArH), 6.53 (t, *J* = 7.4 Hz, 2H, ArH), 2.81 (s, 3H, NCH_3), 2.18 (s, 3H, CH_3); ^{13}C NMR (101 MHz, $CDCl_3$) δ 149.0 (C), 146.8 (C), 146.6 (C), 137.7 (C), 137.1 (C), 131.3 (CH), 130.9 (CH), 129.6 (CH), 128.8 (2 x CH), 127.7 (CH), 125.3 (CH), 124.8 (CH), 120.4 (CH), 118.4 (CH), 115.0 (2 x CH), 39.8 (NCH_3), 20.4 (CH_3); HRMS (ES^+) calculated for $C_{20}H_{18}N_2O_2Na$ 341.1266 [*M*+Na]$^+$, found 341.1277.

Compound 7c

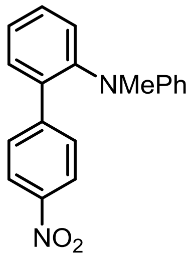

Chemical formula: $C_{19}H_{16}N_2O_2$
MW = 304

N-Methyl-4'-nitro-*N*-phenyl-[1,1'-biphenyl]-2-amine **7c** was synthesised according to general procedure E (0.25 mmol) and was isolated as a yellow solid (44 mg, 58%). R_f 0.60 (EtOAc:hexanes, 1:9); ^{1}H NMR (400 MHz, $CDCl_3$) δ 8.16 (d, *J* = 8.7 Hz, 2H, ArH), 7.54 (d, *J* = 8.7 Hz, 2H, ArH), 7.45 (t, *J* = 8.4 Hz, 2H, ArH), 7.38 – 7.31 (m, 2H, ArH), 7.18 (t, J = 7.9 Hz, 2H, ArH), 6.76 (t, *J* = 7.3 Hz, 1H, ArH), 6.67 (d, *J* = 8.1 Hz, 2H, ArH), 2.90 (s, 3H, CH_3); ^{13}C NMR (101 MHz, $CDCl_3$) δ 149.1 (C), 147.1 (C), 146.8 (C), 146.5 (C), 137.7 (C), 131.3 (CH), 130.4 (CH), 129.5 (2 x CH), 129.2 (2 x CH), 129.1 (CH), 126.6 (CH), 123.7 (2 x CH), 118.1 (CH), 114.1 (2 x CH), 39.6 (CH_3); HRMS (TOF MS EI$^+$) *m/z* Calculated for $C_{19}H_{16}O_2N_2$ 304.1206 [*M*]$^+$, found 304.1198; mp 126 – 130 °C.

Compound 7d

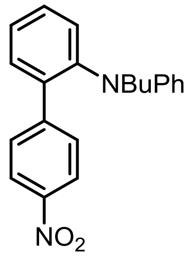

Chemical formula: $C_{22}H_{22}N_2O_2$
MW = 346

N-Butyl-4'-nitro-N-phenyl-[1,1'-biphenyl]-2-amine **7d** was synthesised according to general procedure E (0.25 mmol) and was isolated as a yellow solid (36 mg, 42%). R_f 0.63 (EtOAc:hexanes, 1:9); ^{1}H NMR (400 MHz, $CDCl_3$) δ 8.15 (d, *J* = 8.8 Hz, 2H, ArH), 7.50 (d, *J* = 8.8 Hz, 2H, ArH), 7.45 (t, *J* = 7.8 Hz, 2H, ArH), 7.38 (d, *J* = 7.4 Hz, 1H, ArH), 7.32 (d, *J* = 7.9 Hz, 1H, ArH), 7.17 (t, *J* = 7. Hz, 2H, ArH), 6.74 (t, *J* = 7.3 Hz, 1H, ArH), 6.64 (d, *J* = 8.0 Hz, 2H, ArH), 3.11 (t, *J* = 8.0 Hz, 2H, $NC\mathbf{H_2}CH_2CH_2CH_3$), 1.53 – 1.41 (p, *J* = 7.5 Hz, 2H, $NCH_2CH_2C\mathbf{H_2}CH_3$), 1.17 (h, *J* = 7.4 Hz, 2H, $NCH_2CH_2\mathbf{CH_2}CH_3$), 0.81 (t, *J* = 7.3 Hz, 3H, $NCH_2CH_2CH_2C\mathbf{H_3}$); ^{13}C NMR (101 MHz, $CDCl_3$) δ 148.9 (C), 147.1 (C), 146.9 (C),

145.0 (C), 138.2 (C), 131.5 (CH), 130.7 (CH), 130.2 (CH), 129.6 (2 x CH), 129.3 (2 x CH), 126.7 (CH), 123.7 (2 x CH), 117.7 (CH), 114.2 (2 x CH), 51.4 (N**CH₂**CH₂CH₂CH₃), 28.8 (NCH₂**CH₂**CH₂CH₃), 20.3 (NCH₂CH₂**CH₂**CH₃), 14.0 NCH₂CH₂CH₂**CH₃**); HRMS (TOF MS EI$^+$) *m/z* Calculated for $C_{22}H_{22}O_2N_2$ 346.1676 [*M*]$^+$, found 346.1667.

Compound 7e

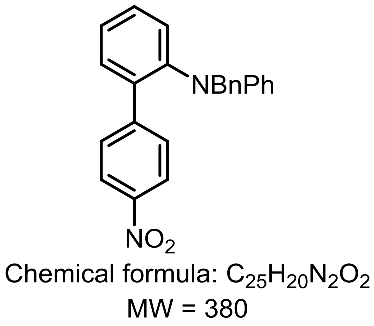

N-Benzyl-4'-nitro-*N*-phenyl-[1,1'-biphenyl]-2-amine **7e** was synthesised according to general procedure E (0.25 mmol) and was isolated as a yellow solid (43 mg, 45%). R_f 0.43 (EtOAc:hexanes, 1:9); ^{1}H NMR (500 MHz, $CDCl_3$) δ 8.17 – 8.12 (m, 2H, ArH), 7.50 – 7.41 (m, 5H, ArH) 7.38 – 7.32 (m, 1H, ArH), 7.24 – 7.07 (m, 7H, ArH), 6.75 (tt, *J* = 7.3, 1.1 Hz, 1H, ArH), 6.70 – 6.60 (m, 2H, ArH), 4.39 (s, 2H, CH_2); ^{13}C NMR (126 MHz, $CDCl_3$) δ 149.2 (C), 147.2 (C), 146.9 (C), 145.2 (C), 138.4 (C), 138.2 (C), 131.7 (CH), 130.6 (2 x CH), 130.3 (2 x CH), 129.7 (2 x CH), 129.3 (CH), 128.5 (2 x CH), 127.0 (CH), 126.9 (2 x CH), 126.8 (CH), 123.8 (2 x CH), 118.4 (CH), 114.6 (2 x CH), 56.2 (CH_2); HRMS (TOF MS EI$^+$) *m/z* calculated for $C_{25}H_{20}O_2N_2$ 380.1519 [*M*]$^+$, found 380.1506; mp 132 - 136 °C.

Compound 7f

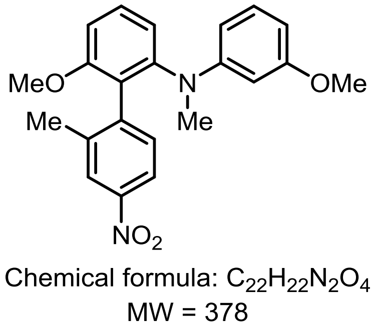

6-Methoxy-*N*-(3-methoxyphenyl)-*N*,2'-dimethyl-4'nitro-[1,1'-biphenyl]-2-amine **7f** was synthesised according to general procedure E (0.46 mmol) and was isolated as an amorphous yellow solid (82 mg, 48%). R_f 0.14 (EtOAc:hexanes, 1:9); ^{1}H NMR (400 MHz, $CDCl_3$) δ 8.05 (d, *J* = 2.5 Hz, 1H, ArH), 7.90 (dd, *J* = 8.5, 2.5 Hz, 1H, ArH), 7.40 (t, *J* = 8.2 Hz, 1H,

ArH), 7.16 (d, J = 8.5, Hz, 1H, ArH), 7.02 (t, J = 8.2, Hz, 1H, ArH), 6.97 (d, J = 8.0, Hz, 1H, ArH), 6.89 (d, J = 8.4 Hz, 1H, ArH), 6.29 (dd, J = 8.0, 2.4 Hz, 1H, ArH), 6.17 (dd, J = 8.4, 2.4 Hz, 1H, ArH), 6.11 (t, J = 2.4 Hz, 1H, ArH), 3.77 (s, 3H, OCH_3), 3.72 (s, 3H, OCH_3), 2.78 (s, 3H, NCH_3), 2.22 (s, 3H, CH_3); ^{13}C NMR (101 MHz, $CDCl_3$) δ 160.3 (C), 157.2 (C), 150.5 (C), 147.6 (C), 146.9 (C), 142.7 (C), 139.1 (C), 131.1 (CH), 130.2 (CH), 129.4 (CH), 126.8 (C), 124.3 (CH), 120.8 (CH), 120.3 (CH), 108.2 (CH), 107.4 (CH), 102.3 (CH), 100.9 (CH), 55.7 (OCH_3), 55.0 (OCH_3), 39.3 (NCH_3), 19.9 (CH_3); HRMS (ES^+) calculated for $C_{22}H_{22}N_2O_4Na$ 401.1477 $[M+Na]^+$, found 401.1477; rt (HPLC) 13.19, 14.46

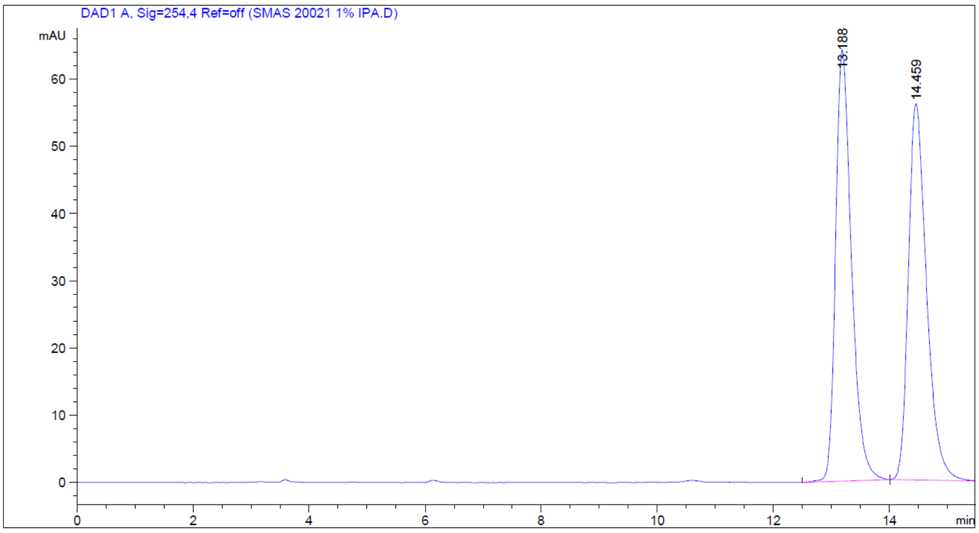

Sulfonamide Scope (Scheme 4)

Compound 4s

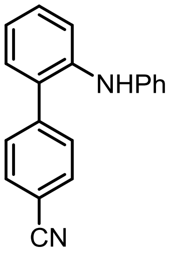

Chemical Formula: $C_{19}H_{14}N_2$
MW = 270

2'-(Phenylamino)-[1,1'-biphenyl]-4-carbonitrile **4s** was synthesized according to general procedure D (0.25 mmol) and was isolated as a yellow oil (34 mg, 50%). R_f 0.38 (EtOAc:hexanes, 1:9); ^1H NMR (400 MHz, $CDCl_3$) δ 7.71 – 7.65 (m, 2H ArH), 7.60 – 7.55 (m, 2H, ArH), 7.38 (dd, J = 8.3, 1.3 Hz, 1H, ArH), 7.29 (td, J = 8.5, 8.0, 1.9 Hz, 1H, ArH), 7.25 – 7.19 (m, 3H, ArH), 7.03 (td, J = 7.4, 1.2 Hz, 1H, ArH), 7.01 – 6.96 (m, 2H, ArH), 6.93

(t, J = 7.4 Hz, 1H, ArH), 5.41 (s, 1H, NH); ^{13}C NMR (101 MHz, $CDCl_3$) δ 144.2 (C), 143.0 (C), 140.1 (C), 132.7 (2 x CH), 130.8 (CH), 130.2 (2 x CH), 130.0 (C), 129.5 (CH), 129.5 (2 x CH), 121.9 (CH), 121.5 (CH), 118.9 (CN), 118.7 (CH), 118.7 (2 x CH), 111.2 (C); HRMS (TOF MS AP$^+$) *m/z* Calculated for $C_{19}H_{14}N_2H$ 271.1235 [*M*]$^+$, found 271.1245.

Compound 4t

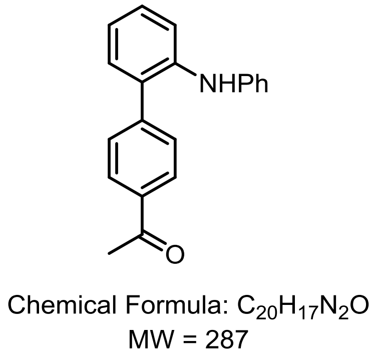

1-(2'-(Phenylamino)-[1,1'-biphenyl]-4-yl)ethan-1-one **4t** was synthesized according to general procedure D (0.25 mmol) and was isolated as a white solid (50 mg, 69%). R_f 0.23 (EtOAc:hexanes 1:9) ^{1}H NMR (400 MHz, $CDCl_3$) δ 8.08 – 8.01 (m, 2H, ArH), 7.63 – 7.57 (m, 2H, ArH), 7.45 (dd, J = 8.2, 1.2 Hz, 1H, ArH), 7.37 – 7.24 (m, 4H, ArH), 7.11 – 7.03 (m, 3H, ArH), 7.00 – 6.95 (m, 1H, ArH), 5.58 (s, 1H, NH), 2.66 (s, 3H, CH_3); ^{13}C NMR (101 MHz, $CDCl_3$) δ 197.8 (C=O), 144.2 (C), 143.1 (C), 140.1 (C), 136.0 (C), 130.8 (CH), 130.6 (C), 129.6 (2 x CH), 129.5 (2 x CH), 129.1 (2 x CH), 129.0 (CH), 121.6 (CH), 121.3 (CH), 118.2 (2 x CH), 118.2 (CH), 26.81 (CH_3); HRMS (TOF MS ES$^+$) *m/z* calculated for $C_{20}H_{17}NONa$ 310.1208 [*M*+Na]$^+$, found 310.12136; mp 138 °C.

Compound 4u

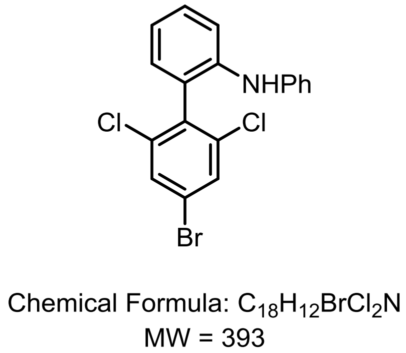

4'-Bromo-2',6'-dichloro-*N*-phenyl-[1,1'-biphenyl]-2-amine **4u** was synthesised according to general procedure D (0.46 mmol) and isolated as a white solid (158 mg, 88%). R_f 0.52 (EtOAc:hexanes 1:9); ^{1}H NMR (500 MHz, $CDCl_3$) δ 7.61 (s, 2H, ArH), 7.38 – 7.32 (m, 2H,

ArH), 7.25 – 7.22 (m, 2H, ArH), 7.09 (dd, J = 7.6, 1.7 Hz, 1H, ArH), 7.06 – 7.04 (m, 1H, ArH), 7.03 – 7.01 (m, 2H, ArH), 6.96 – 6.92 (m, 1H, Ar), 5.07 (brs, 1H, NH); ^{13}C NMR (125 MHz, CDCl3) δ 142.8 (C), 141.2 (C), 136.8 (2 x C), 135.7 (C), 131.1 (2 x CH), 130.5 (CH), 129.6 (CH), 129.2 (2 x CH), 125.8 (C), 121.9 (C), 121.6 (CH), 121.0 (CH), 119.0 (2 x CH), 117.7 (CH); HRMS (TOF MS AP$^+$) *m/z* calculated for $C_{18}H_{13}NCl_2Br$ 391.9608 [*M*+H]$^+$, found 391.9615; mp: 72 °C.

Compound 4v

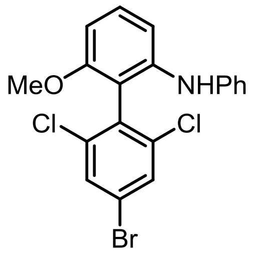

Chemical Formula: $C_{19}H_{14}BrCl_2NO$
MW = 423

4'-Bromo-2',6'-dichloro-6-methoxy-*N*-phenyl-[1,1'-biphenyl]-2-amine **4v** was synthesised according to general procedure D (0.25 mmol) and was isolated as a white solid (78 mg, 79%). R_f 0.52 (EtOAc:hexanes 1:9); ^{1}H NMR (400 MHz, $CDCl_3$) δ 7.60 (s, 2H, ArH), 7.29 (t, J = 8.3 Hz, 1H, ArH), 7.23 (tt, J = 7.4, 2.0 Hz, 2H, ArH), 7.06 – 7.02 (m, 2H, ArH), 6.98 (dd, J = 8.4, 0.9 Hz, 1H, ArH), 6.97 – 6.92 (m, 1H, ArH), 6.58 (dd, J = 8.3, 0.9 Hz, 1H, ArH), 5.02 (s, 1H, NH), 3.75 (s, 3H, OCH_3); ^{13}C NMR (101 MHz, $CDCl_3$) δ 157.6 (C), 142.7 (C), 142.4 (C), 137.5 (CH), 132.6 (C), 131.2 (2 x CH), 130.3 (CH), 129.3 (2 x CH), 122.0 (CH), 121.9 (C), 119.8 (2 x CH), 114.2 (C), 109.8 (CH), 103.3 (CH), 56.0 (OCH_3); HRMS (TOF MS AP$^+$) *m/z* calculated for $C_{19}H_{15}NOCl_2Br$ 421.9714 [*M*+H], found 421.9749; Mp 120 °C.

Compound 4w

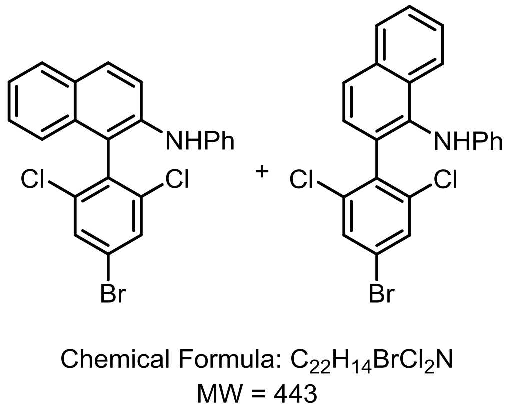

1-(4-Bromo-2,6-dichlorophenyl)-*N*-phenylnaphthalen-2-amine and 2-(4-bromo-2,6-dichlorophenyl)-*N*-phenylnaphthalen-1-amine **4w** were synthesised, as an inseparable mixture, according to general procedure D (0.25 mmol) and were isolated as a colourless crystalline solid (71 mg, 65%, 2.5:1). R_f 0.62 (EtOAc:hexanes 1:9); ^{1}H NMR (400 MHz, $CDCl_3$) δ 7.91 (d, *J* = 8.5 Hz, 1H, ArH), 7.81 (d, *J* = 8.2 Hz, 1H, ArH), 7.71 (dd, *J* = 9.1, 5.6 Hz, 9H, ArH), 7.58 (s, 8H, ArH), 7.50 – 7.37 (m, 7H, ArH), 7.32 (ddd, *J* = 8.3, 6.9, 1.3 Hz, 1H, ArH), 7.26 – 7.19 (m, 8H, ArH), 7.19 – 7.08 (m, 10H, ArH), 6.94 (dq, *J* = 8.7, 2.3 Hz, 14H, ArH), 6.86 (tt, *J* = 7.2, 1.2 Hz, 4H, ArH), 6.60 (tt, *J* = 7.2, 1.2 Hz, 1H, ArH), 6.48 – 6.36 (m, 2H, ArH), 5.15 (s, 4H, NH); ^{13}C NMR (101 MHz, $CDCl_3$) δ 146.4, 142.8, 139.0, 137.9, 136.7, 136.2, 135.9, 134.8, 133.9, 132.3, 131.6, 131.0, 130.7, 130.0, 129.9, 129.5, 129.3, 129.0, 128.5, 128.5, 127.7, 127.2, 126.9, 126.5, 126.1, 125.0, 123.9, 123.3, 122.5, 122.1, 121.7, 119.5, 119.18, 119.17, 119.0, 115.3; HRMS (TOF MS AP$^+$) *m/z* calculated for $C_{22}H_{15}NCl_2Br$ 441.9765 [*M*]$^+$, found 441.9751.

Compound 4x

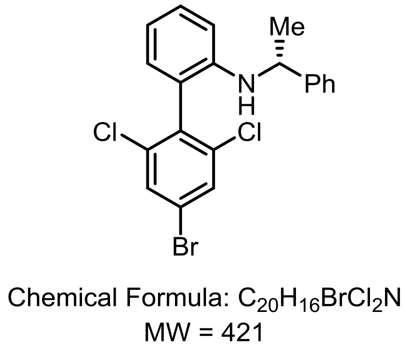

(R)-4'-Bromo-2',6'-dichloro-*N*-(1-phenylethyl)-[1,1'-biphenyl]-2-amine **4x** was synthesised according to general procedure E (0.25 mmol) and was isolated as a pale yellow oil (66 mg,

63%). R_f = 0.63 (EtOAc:hexanes, 1:9); ^{1}H NMR (400 MHz, $CDCl_3$) δ 7.68 (d, *J* = 1.9 Hz, 1H, ArH), 7.66 (d, *J* = 1.9 Hz, 1H, ArH), 7.37 – 7.26 (m, 5H, ArH), 7.25 – 7.20 (m, 1H, ArH), 7.15 (ddd, *J* = 8.2, 7.4, 1.6 Hz, 1H, ArH), 6.92 (dd, *J* = 7.5, 1.7 Hz, 1H, ArH), 6.76 (td, *J* = 7.4, 1.1 Hz, 1H, ArH), 6.51 – 6.45 (m, 1H, ArH), 4.58 – 4.48 (m, 1H, NHC**H**), 3.52 (brs, 1H, N**H**CH), 1.41 (d, *J* = 6.7 Hz, 3H, CH_3); ^{13}C NMR (101 MHz, $CDCl_3$) δ 145.1 (C), 144.0 (C), 137.5 (C), 137.1 (C), 135.9 (C), 131.5 (CH), 131.3 (CH), 129.9 (CH), 129.7 (CH), 128.7 (2 x CH), 127.0 (CH), 125.9 (2 x CH), 122.1 (C), 121.5 (C), 117.1 (CH), 112.2 (CH), 53.4 (NCH), 25.3 (CH_3); HRMS (FTMS APCI$^+$) *m/z* calculated for $C_{20}H_{17}BrCl_2N$ 419.9916 $[M+H]^+$, found 419.9917.

Compound 4y

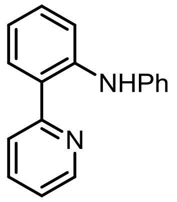

N-Phenyl-2-(pyridin-2-yl)aniline **4y** was synthesised according to general procedure D (0.25 mmol) was isolated as a white solid (29 mg, 42%). R_f 0.46 (EtOAc:hexanes, 1:9); ^{1}H NMR (500 MHz, $CDCl_3$) δ 10.14 (s, 1H, NH), 8.57 – 8.52 (m, 1H, ArH), 7.70 (td, *J* = 7.8, 1.9 Hz, 1H, ArH), 7.63 (dt, *J* = 8.1, 1.1 Hz, 1H, ArH), 7.53 (dd, *J* = 7.8, 1.6 Hz, 1H, ArH), 7.39 (dd, *J* = 8.3, 1.2 Hz, 1H, ArH), 7.18 (dtd, *J* = 8.5, 6.9, 1.8 Hz, 3H, ArH), 7.15 – 7.09 (m, 3H, ArH), 6.88 – 6.81 (m, 2H, ArH); ^{13}C NMR (126 MHz, $CDCl_3$) δ 159.2 (C), 147.6 (CH), 143.1 (C), 142.9 (C), 137.2 (CH), 130.0 (CH), 129.8 (CH), 129.4 (2 x CH), 124.9 (C), 122.9 (CH), 121.4 (CH), 121.3 (CH), 119.6 (2 x CH), 119.4 (CH), 116.8 (CH); HRMS (FTMS ESI$^+$) calculated for $C_{17}H_{15}N_2$ 247.1230 $[M+H]^+$ 247.1234. Data are consistent with literature values.[14]

Compound 4z

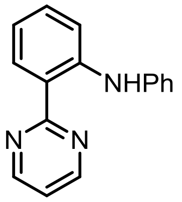

Chemical Formula: $C_{16}H_{14}N_3$
MW = 247

N-Phenyl-2-(pyrimidin-2-yl)aniline **4z** was synthesised according to general procedure D (0.35 mmol) and was isolated as a yellow oil (48 mg, 55%). R_f 0.27 (EtOAc:hexanes, 1:9); ^{1}H NMR (400 MHz, $CDCl_3$) δ 10.67 (s, 1H, NH), 8.69 (d, *J* = 4.9 Hz, 2H, ArH), 8.44 (dd, *J* = 8.0, 1.7 Hz, 1H, ArH), 7.32 (dd, *J* = 8.5, 1.2 Hz, 1H, ArH), 7.28 – 7.13 (m, 5H, ArH), 7.03 (t, *J* = 4.9 Hz, 1H, ArH), 6.93 (tt, *J* = 7.0, 1.6 Hz, 1H, ArH), 6.81 (ddd, *J* = 8.2, 7.0, 1.3 Hz, 1H, ArH); ^{13}C NMR (101 MHz, $CDCl_3$) δ 165.8 (C), 156.3 (2 x CH), 145.7 (C), 142.2 (C), 131.7 (CH), 131.4 (CH), 129.4 (2 x CH), 122.4 (CH), 121.4 2 x CH), 120.8 (C), 118.4 (CH), 117.7 (CH), 115.5 (CH); HRMS (TOF MS AP$^+$) Calculated for $C_{16}H_{15}N_3$ 248.1188 [*M*+H]$^+$, found 248.1181.

Compound 4zz

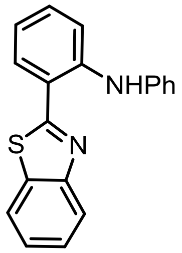

Chemical Formula: $C_{19}H_{14}N_2S$
MW = 302

2-(Benzo[d]thiazol-2-yl)-*N*-phenylaniline **4zz** was synthesised according to general procedure D (0.25 mmol) and was isolated as a yellow solid (22 mg, 74%). R_f 0.74 (EtOAc:hexane, 1:9); ^{1}H NMR (400 MHz, $CDCl_3$) δ 10.69 (s, 1H, NH), 7.88 (d, *J* = 8.1 Hz, 1H, ArH), 7.78 (d, *J* = 7.9 Hz, 1H, ArH), 7.70 (d, *J* = 7.9 Hz, 1H, ArH), 7.36 (t, *J* = 7.9 Hz, 1H, ArH), 7.31 – 7.23 (m, 5H, ArH), 7.15 (t, *J* = 8.5 Hz, 1H, ArH), 6.99 (t, *J* = 6.6 Hz, 1H, ArH), 6.73 (t, *J* = 7.5 Hz, 1H, ArH); ^{13}C NMR (101 MHz, $CDCl_3$) δ 169.1 (C), 153.5 (C), 144.2 (C), 141.5 (C), 133.4 (C), 131.6 (CH), 130.9 (CH), 129.5 (2 x CH), 126.3 (CH), 125.2 (CH), 123.3 (CH), 122.6 (CH), 122.4 (2 x CH), 121.3 (CH), 118.0 (CH), 116.7 (C), 114.6

(CH); HRMS (TOF MS AP^{+}) *m/z* Calculated for $C_{19}H_{15}N_2S$ 303.0956 [*M*+H]$^{+}$, found 303.0948.

5. Characterisation data for derivative compounds

Compound 8a

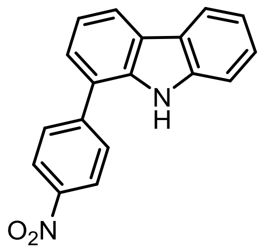

Chemical formula: $C_{18}H_{12}N_2O_2$
MW = 288

1-(4-Nitrophenyl)-9*H*-carbazole **8a** was synthesised according to a modified literature procedure.[15] *N*-(2-Bromophenyl)-4'-nitro-[1,1'-biphenyl]-2-amine **4o** (50 mg, 0.13 mmol, 1 eq.), palladium acetate (2.2 mg, 0.01 mmol, 10 mol%), PCy_3HBF_4 (7.4 mg, 0.02 mmol, 20 mol%), oven dried potassium carbonate (37 mg, 0.27 mmol, 2 eq.) were dissolved in degassed DMA (0.65 mL, 0.2M) and heated to reflux for 24 hours. The crude reaction mixture was filtered through a pad of Celite© and then purified by column chromatography (SiO_2, 0 – 10% EtOAc:hexanes) to afford the title compound as a yellow solid (33 mg, 86%). R_f 0.40 (EtOAc:hexanes, 1:9); ^{1}H NMR (400 MHz, DMSO-d_6) δ 7.38 (d, J = 8.5 Hz, 2H), 7.00 (d, J = 8.5 Hz, 2H), 6.34 (d, J = 8.2 Hz, 2H), 6.29 – 6.25 (m, 3H), 6.22 (d, J = 7.7 Hz, 1H), 6.07 (dd, J = 10.5, 7.6 Hz, 5H), 5.76 (d, J = 7.9 Hz, 2H), 5.66 (t, J = 7.3 Hz, 1H); ^{13}C NMR (101 MHz, DMSO-d_6) δ 149.9, 145.3, 145.2, 139.86, 136.0, 135.9, 133.5, 131.0, 129.8, 129.0, 128.4, 124.8, 123.1, 122.4, 120.5, 118.6, 115; *m/z* (ES^{+}) 287 ([M-H]$^{-}$, 100%). Data are consistent with the literature values.[16]

Compound 8b

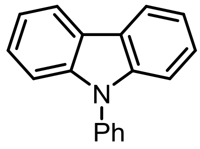

Chemical Formula: $C_{18}H_{13}N$
Molecular Weight: 243

The preparation procedure of 9-phenyl-9*H*-carbazole **8b** from 2’-nitro-*N*-phenyl-[1,1’-biphenyl]-2-amine **4r** was adapted from a literature procedure describing a synthesis of

dibenzo[*b*,*d*]furan from 2’-nitro-[1,1’-biphenyl]-2-ol.[17] To a suspension of NaH (11.5 mg, 0.48 mmol) in hexamethylphosphoric triamide (1.0 mL) was added a solution of 2’-nitro-*N*-phenyl-[1,1’-biphenyl]-2-amine (70 mg, 0.24 mmol) in hexamethylphosphoric triamide (1.0 mL). The reaction mixture was heated at 70°C with vigorous stirring. After 22 hours the starting material had been consumed (TLC analysis). The reaction mixture was cooled and poured into 5% HCl followed by extraction of the aqueous layer with EtOAc. The organic layer was washed with water and brine, dried over Na_2SO_4, filtered and evaporated. The crude product was purified by column chromatography (SiO_2, 1:1 v/v EtOAc:hexane) to give the pure compound as greenish crystalline solid (41 mg, 71%). R_f 0.39 (1:19 EtOAc:hexane); ^{1}H NMR (400 MHz, CD_2Cl_2) δ 8.15(dt, *J* = 7.8, 1.1 Hz, 2H), 7.65 – 7.61 (m, 2H), 7.59 – 7.57 (m, 2H), 7.51 – 7.47 (m, 1H), 7.42 – 7.41 (m, 4H), 7.30 – 7.26 (m, 2H); ^{13}C NMR (101 MHz, $CDCl_3$) δ 140.8, 137.6, 129.8, 127.4, 127.1, 125.9, 123.3, 120.3, 119.8, 109.7; HRMS (TOF MS AP$^+$) calculated for $C_{18}H_{13}NH$ [*M*+H]$^+$ 244.1126, found 244.1114; Mp: 96 ^{o}C. The analytical data are consistent with commercial sample (CAS 1150-62-5).

Compound 12

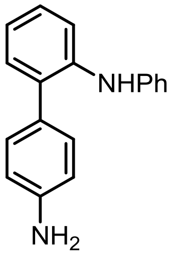

Chemical formula: $C_{18}H_{16}N_2$
MW = 260

4'-Nitro-*N*-phenyl-[1,1'-biphenyl]-2-amine **9** (145 mg, 0.5 mmol) was dissolved in EtOH (25 mL) and was reduced using a Thales Nano H-Cube flow reactor with 10% Pd/C at ambient temperature (30 bar, 1 mL/min). The crude reaction mixture was purified by column chromatography (SiO_2 gel, 0 – 1:1 *v/v* EtOAc:hexanes) to afford the title compound as a light brown oil (113 mg, 86%). R_f 0.11 (EtOAc:hexane 1:9); ^{1}H NMR (400 MHz, $CDCl_3$) δ 7.26 (d, *J* = 8.1 Hz, 1H, ArH), 7.17 – 7.05 (m, 6H, ArH), 6.95 – 6.89 (m, 2H, ArH), 6.88 – 6.83 (m, 1H, ArH), 6.79 (t, *J* = 7.3 Hz, 1H, ArH), 6.59 (d, *J* = 8.5 Hz, 2H, ArH), 5.56 (s, 1H, NH), 3.54 (s, 2H, NH_2); ^{13}C NMR (101 MHz, $CDCl_3$) δ 145.9 (C), 143.7 (C), 140.2 (C), 131.8 (C), 130.9 (CH), 130.4 (2 x CH), 129.4 (2 x CH), 128.9 (C), 127.6 (CH), 121.1 (CH), 120.9 (CH), 118.0 (2 x CH), 117.4 (CH), 115.4 (2 x CH); HRMS (TOF MS ES$^+$) *m/z* Calculated for $C_{18}H_{17}N_2$ 261.1392 [*M*+H]$^+$, found 261.1399.

Compound 9

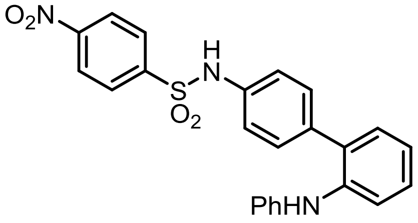

Chemical formula: $C_{24}H_{19}N_3O_4S$
MW = 446

4-Nitrobezenesulfonyl chloride (94 mg, 0.42 mmol) was dissolved in dry CH_2Cl_2 (7 mL) and added to N^2-phenyl-[1,1'-biphenyl]-2,4'-diamine **12** (110mg, 0.42 mmol) and dry pyridine (34 µL, 0.46 mmol, 1.1 eq.) in dry CH_2Cl_2 (7 mL). The reaction mixture was stirred at ambient temperature for 4 hours and then acidified with 1 M HCl. The aqueous phase was extracted with CH_2Cl_2 (2 mL x 3); the combined organics were washed with brine (10 mL) and dried over $MgSO_4$, filtered and concentrated under vacuum. The crude reaction mixture was purified by column chromatography (SiO_2, 10 - 40% *v/v* EtOAc:hexanes) to afford the title compound as a yellow solid (82 mg, 44%). R_f 0.09 (EtOAc:hexanes 1:9); ^{1}H NMR (400 MHz, DMSO-d_6) δ 7.38 (d, J = 8.5 Hz, 2H, ArH), 7.00 (d, J = 8.5 Hz, 2H, ArH), 6.34 (d, J = 8.2 Hz, 2H, ArH), 6.29 – 6.24 (m, 3H, ArH), 6.22 (d, J = 7.7 Hz, 1H, ArH), 6.07 (dd, J = 10.5, 7.6 Hz, 5H, ArH), 5.76 (d, J = 7.9 Hz, 1H, NH), 5.66 (t, J = 7.3 Hz, 1H, NH); ^{13}C NMR (101 MHz, DMSO) δ 149.9 (C), 145.3 (C), 145.2 (C), 139.8 (C), 136.0 (C), 135.9 (C), 133.5 (C), 131.0 (C), 129.8 (2 x CH), 129.0 (2 x CH), 128.4 (2 x CH), 124.8 (2 x CH), 123.1 (CH), 122.4 (CH), 120.5 (2 x CH), 118.6 (CH), 115.4 (2 x CH); HRMS (TOF MS ES$^-$) *m/z* calculated For 444.1018 [*M*-H]$^-$, found 444.1003; mp 218 – 220°C.

Compound 10

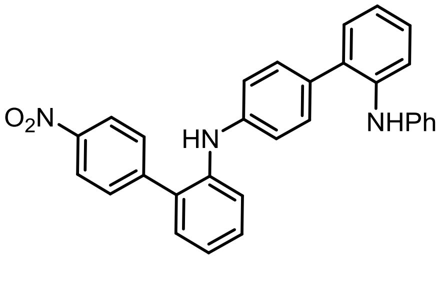

Chemical formula: $C_{30}H_{23}N_3O_2$
MW = 458

2-(Trimethylsilyl)phenyl trifluoromethanesulfonate (44 µL, 0.18 mmol, 1 eq.) was added to 4-nitro-*N*-(2'-(phenylamino)-[1,1'-biphenyl]-4-yl)benzenesulfonamide (82 mg, 0.18 mmol),

KF (31 mg, 0.54 mmol, 3 eq.), 18-crown-6 (143 mg, 0.54 mmol, 3 eq.) in THF (1.8 mL, 0.1 M). The reaction mixture was stirred at reflux for 24 hours and then cooled to ambient temperature. The crude reaction mixture was purified by column chromatography (SiO_2, 0 to 1:9 *v/v* EtOAc:hexanes) to afford the title compound as a red solid (55 mg, 67%). R_f 0.25 (EtOAc:hexane 1:9); ^{1}H NMR (400 MHz, $CDCl_3$) δ 8.28 (d, *J* = 8.7 Hz, 2H, ArH), 7.65 (d, *J* = 8.7 Hz, 2H, ArH), 7.47 (d, *J* = 8.1 Hz, 1H, ArH), 7.36 (d, *J* = 7.7 Hz, 2H, ArH), 7.32 (d, *J* = 8.4 Hz, 2H, ArH), 7.30 – 7.19 (m, 5H, ArH), 7.09 (t, *J* = 7.4 Hz, 1H, ArH), 7.06 – 7.02 (m, 4H, ArH), 6.98 (t, *J* = 7.4 Hz, 1H, ArH), 6.92 (t, *J* = 7.3 Hz, 1H, ArH), 5.63 (s, 1H, NH), 5.49 (s, 1H, NH); ^{13}C NMR (101 MHz, $CDCl_3$) δ 147.2, 146.2, 143.6, 142.5, 140.3, 139.8, 131.8, 131.4, 131.0, 130.9, 130.5, 130.4, 130.2, 129.8, 129.5, 128.0, 124.3, 122.5, 121.3, 121.1, 119.5, 118.2, 118.0, 117.7; HRMS (TOF MS ES$^+$) *m/z* calculated for $C_{30}H_{24}N_3O_2$ 458.1869 [*M*+H]$^+$, found 458.1865; Mp 214 – 216 °C.

Compound 11

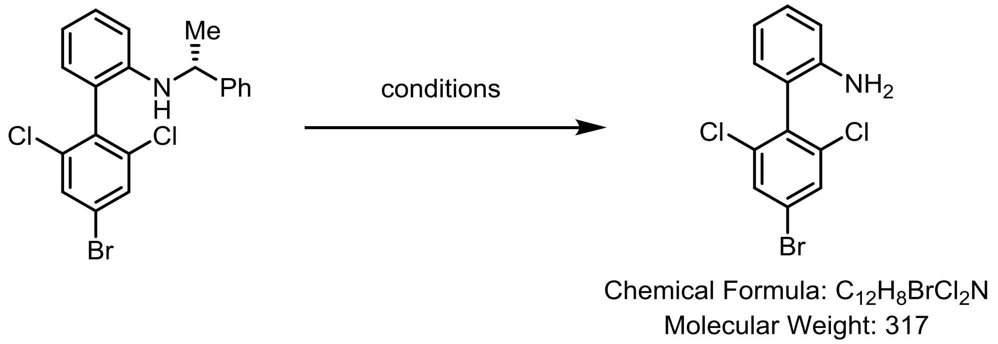

4'-Bromo-2',6'-dichloro-[1,1'-biphenyl]-2-amine **11** was synthesised according to modified literature procedure.[18] Conc. HCl (4mL) was added to (R)-4'-bromo-2',6'-dichloro-*N*-(1-phenylethyl)-[1,1'-biphenyl]-2-amine **4x** (122 mg, 0.29 mmol) in a microwave vial and then sealed then the mixture was heated to 100 °C for 24 hours. The reaction was cooled to 0 °C, quenched with sat. $NaHCO_3$, and then diluted with EtOAc (20 mL). The aqueous phase was washed thrice with EtOAc and then the combined organics were washed with sat. brine and dried over $MgSO_4$, filtered and concentrated under vacuum. The crude reaction mixture was purified by column chromatography (SiO_2, 10% EtOAc:hexane) to afford a white solid (65%, 64 mg, 0.19 mmol). R_f 0.44 (EtOAc:Hexane, 1:9); ^{1}H NMR (400 MHz, $CDCl_3$) δ 7.62 (s, 2H, ArH), 7.29 – 7.22 (m, 1H, ArH), 6.96 (ddd, *J* = 7.7, 1.6, 0.5 Hz, 1H, ArH), 6.87 (td, *J* = 7.4, 1.1 Hz, 1H, ArH), 6.82 (ddd, *J* = 7.9, 1.1, 0.5 Hz, 1H, ArH), 3.32 (brs, 2H, NH); ^{13}C NMR (101 MHz, $CDCl_3$) δ 143.5, 136.9, 135.8, 131.3, 130.1, 130.0, 122.0, 121.9, 118.8, 115.9;

HRMS (FTMS, ESI^+) calculated for $C_{12}H_8BrCl_2N$ $[M+H]^+$ 315.9290, found 315.9195; Mp 106-108 °C.

6. References

[1] M. Jafarpour, A. Rezaeifard, T. Golshani, *Phosphorus, Sulfur, and Silicon* **2010**, *186*, 140-148.

[2] A. Le Pera, A. Leggio, A. Liguori, *Tetrahedron* **2006**, *62*, 6100-6106.

[3] H. R. Lawrence, A. Kazi, Y. Luo, R. Kendig, Y. Ge, S. Jain, K. Daniel, D. Santiago, W. C. Guida, S. M. Sebti, *Bioorg. Med. Chem.* **2010**, *18*, 5576-5592.

[4] B. Flatt, X.-H. Gu, R. Martin, R. Mohan, B. Murphy, M. C. Nyman, W. C. Stevens, Jr., T.-L. Wang, Exelixis, Inc., USA . **2006**, p. 196 pp.

[5] C. Guisado, J. E. Waterhouse, W. S. Price, M. R. Jorgensen, A. D. Miller, *Org. Biomol. Chem.* **2005**, *3*, 1049-1057.

[6] H. Guo, Q. Xu, O. Kwon, *J. Am. Chem. Soc.* **2009**, *131*, 6318-6319.

[7] D. Rennison, D. Conole, M. D. Tingle, J. Yang, C. T. Eason, M. A. Brimble, *Bioorg. Med. Chem. Lett.* **2013**, *23*, 6629-6635.

[8] E. M. Bissinger, R. Heinke, A. Spannhoff, A. Eberlin, E. Metzger, V. Cura, P. Hassenboehler, J. Cavarelli, R. Schule, M. T. Bedford, W. Sippl, M. Jung, *Bioorg. Med. Chem.* **2011**, *19*, 3717-3731.

[9] S. Y. Moon, J. Nam, K. Rathwell, W. S. Kim, *Org. Lett.* **2014**, *16*, 338-341.

[10] A. Gioiello, E. Rosatelli, M. Teofrasti, P. Filipponi, R. Pellicciari, *ACS Comb. Sci.* **2013**, *15*, 235-239.

[11] S. W. Wright, K. N. Hallstrom, *J. Org. Chem.* **2006**, *71*, 1080-1084.

[12] J. Bornholdt, J. Felding, R. P. Clausen, J. L. Kristensen, *Chem. - Eur. J.* **2010**, *16*, 12474-12480.

[13] A. Jirgensons, A. Nikitjuka, A. Nekrasova, *Synlett* **2014**, *26*, 183-186.

[14] J. Du, Y. Yang, H. Feng, Y. Li, B. Zhou, *Chem. - Eur. J.* **2014**, *20*, 5727-5731.

[15] L. C. Campeau, M. Parisien, A. Jean, K. Fagnou, *J. Am. Chem. Soc.* **2006**, *128*, 581-590.

[16] J.-H. Chu, C.-C. Wu, D.-H. Chang, Y.-M. Lee, M.-J. Wu, *Organometallics* **2013**, *32*, 272-282.

[17] A. F. Sierakowski, *Aust. J. Chem.* **1983**, *36*, 1281.

[18] R. T. Michael Fisher, *Synthesis* **2003**, *10*, 1603-1609.

7. NMR Spectra for biaryls

^1H and ^{13}C Spectra ($CDCl_3$, 400 and 101 MHz) for compound 4a

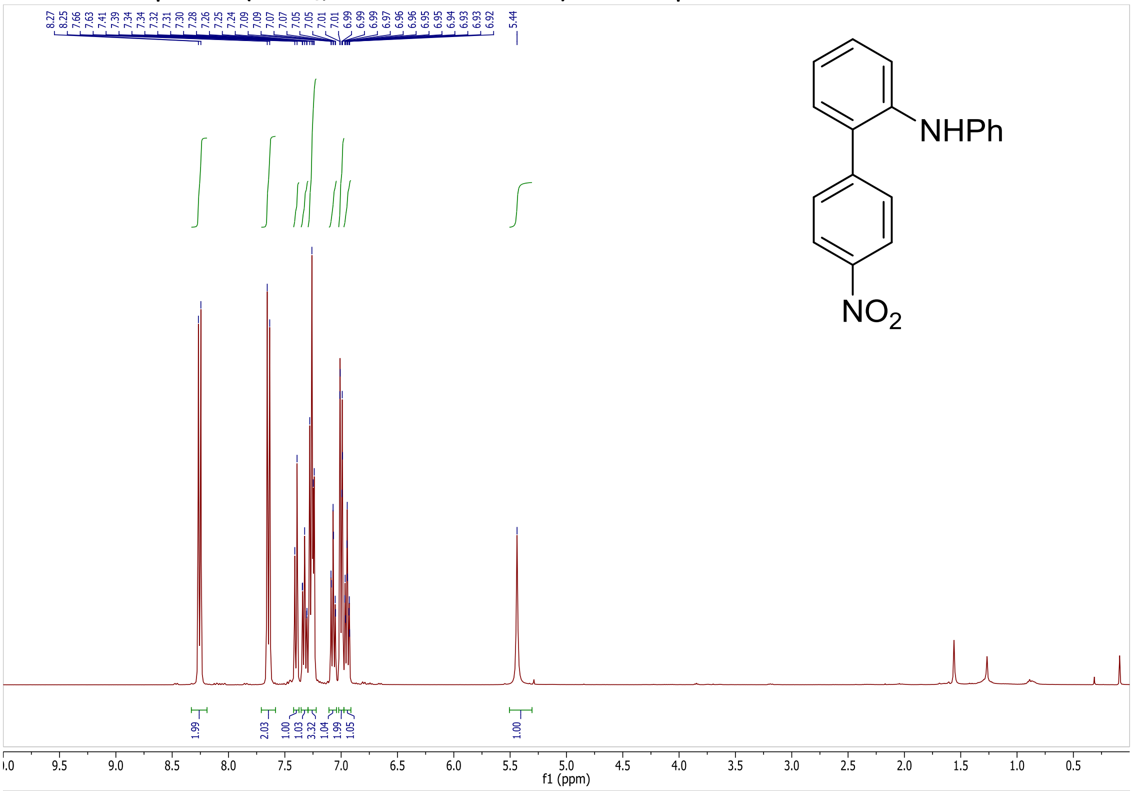

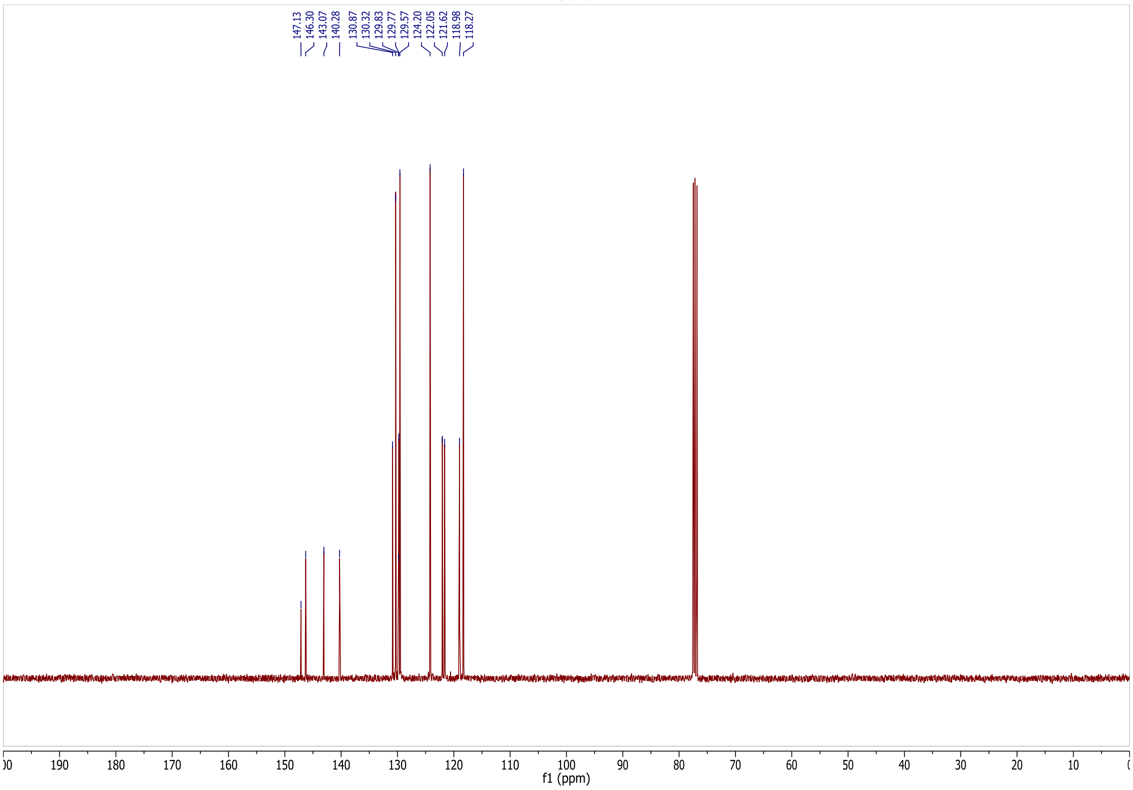

^{1}H and ^{13}C Spectra ($CDCl_3$, 400 and 101 MHz) for compound 4b

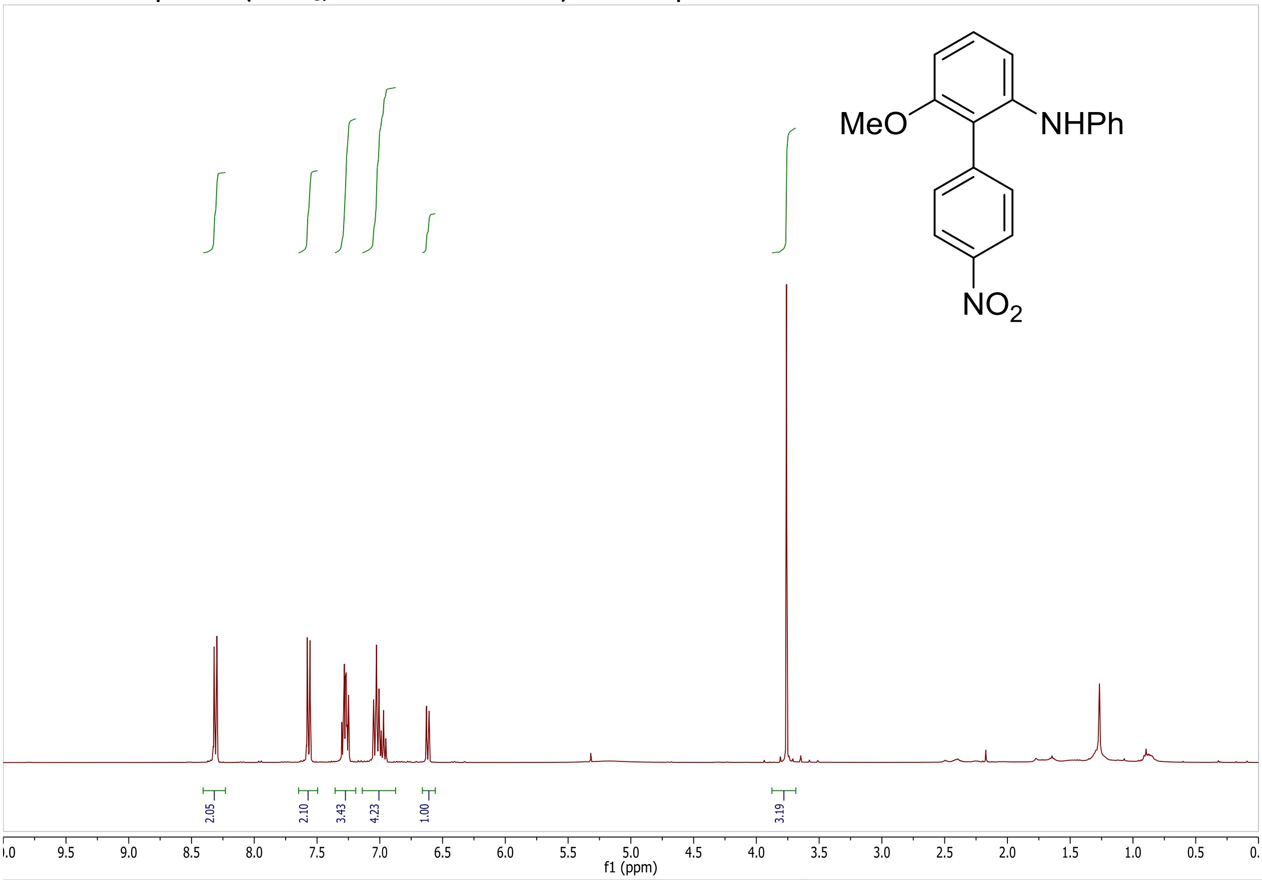

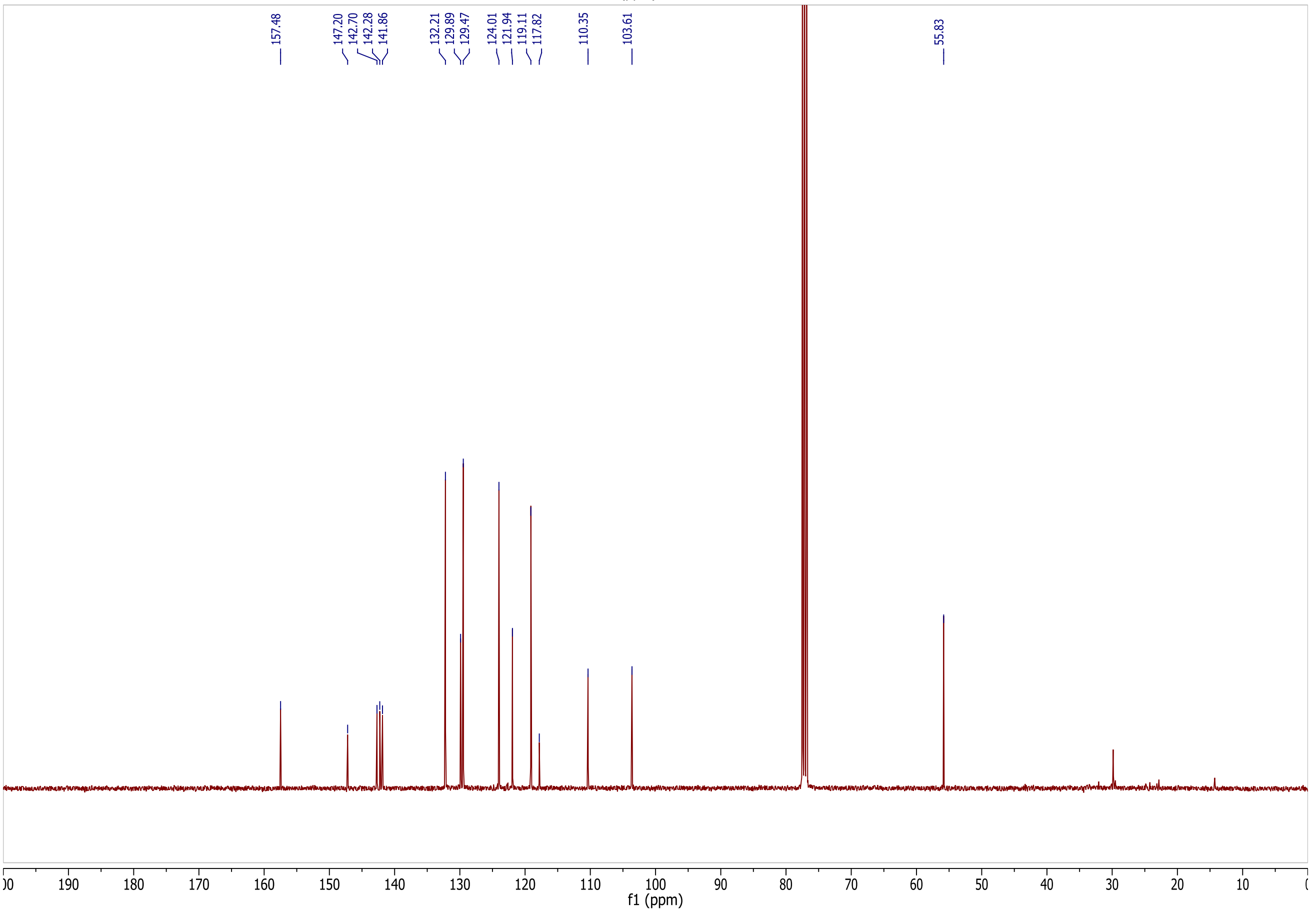

[1]H and [13]C Spectra ($CDCl_3$, 500 and 126 MHz) for compound 4c

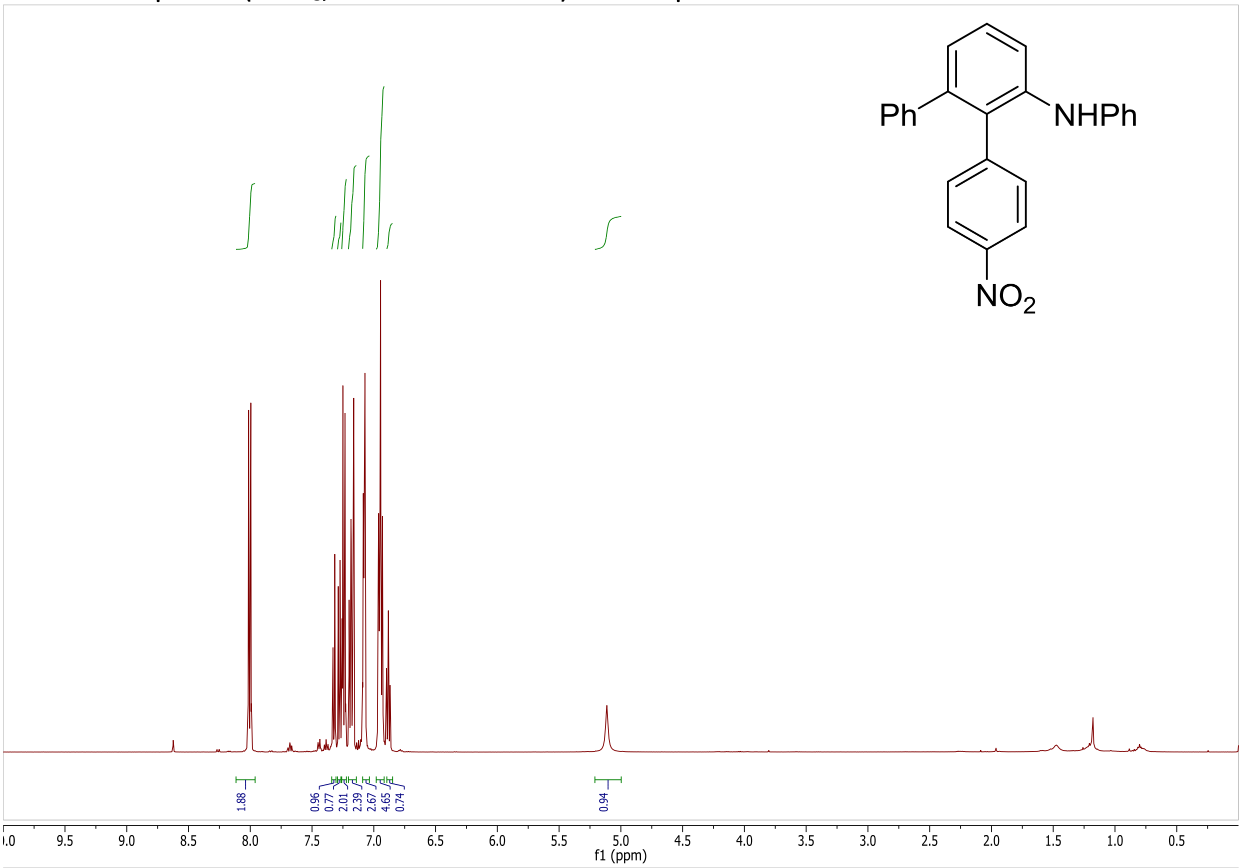

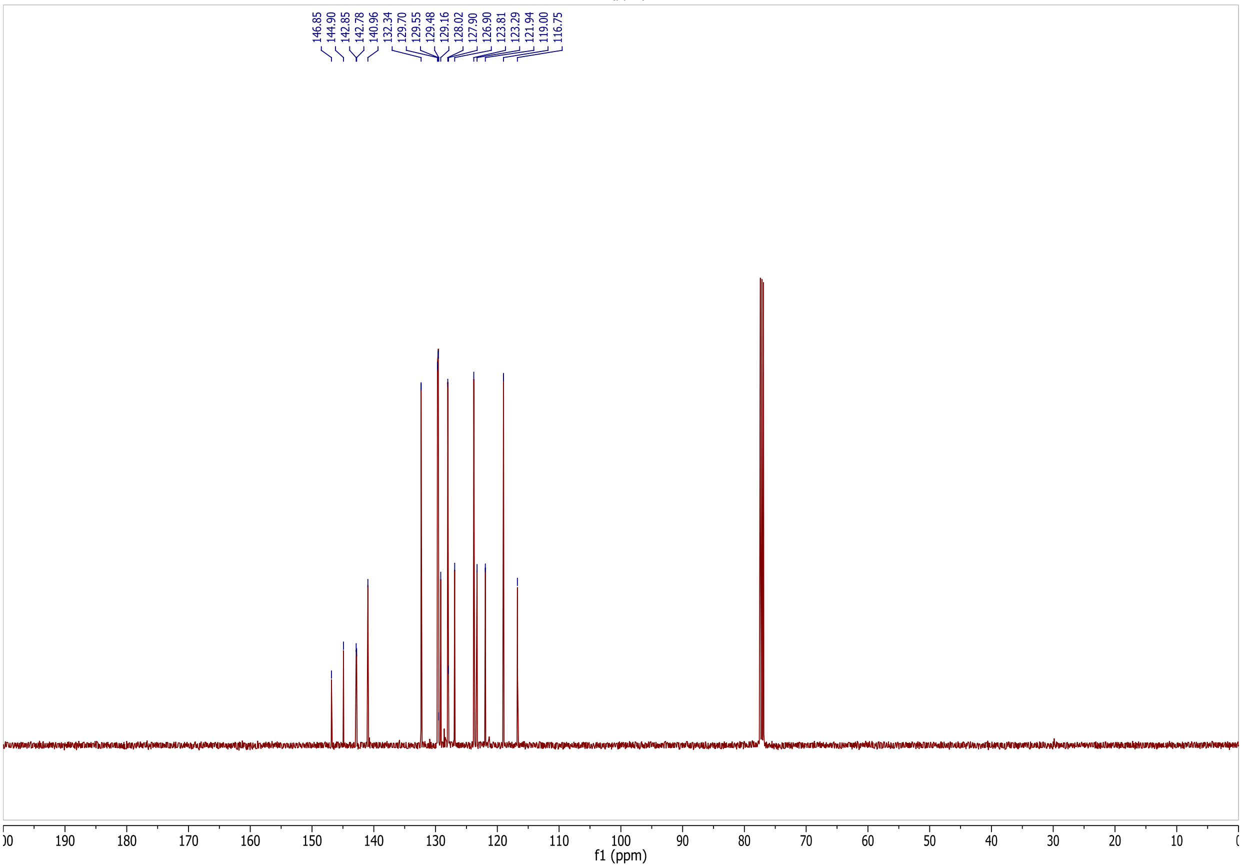

^{1}H and ^{13}C Spectra ($CDCl_3$, 400 and 101 MHz) for compound 4d

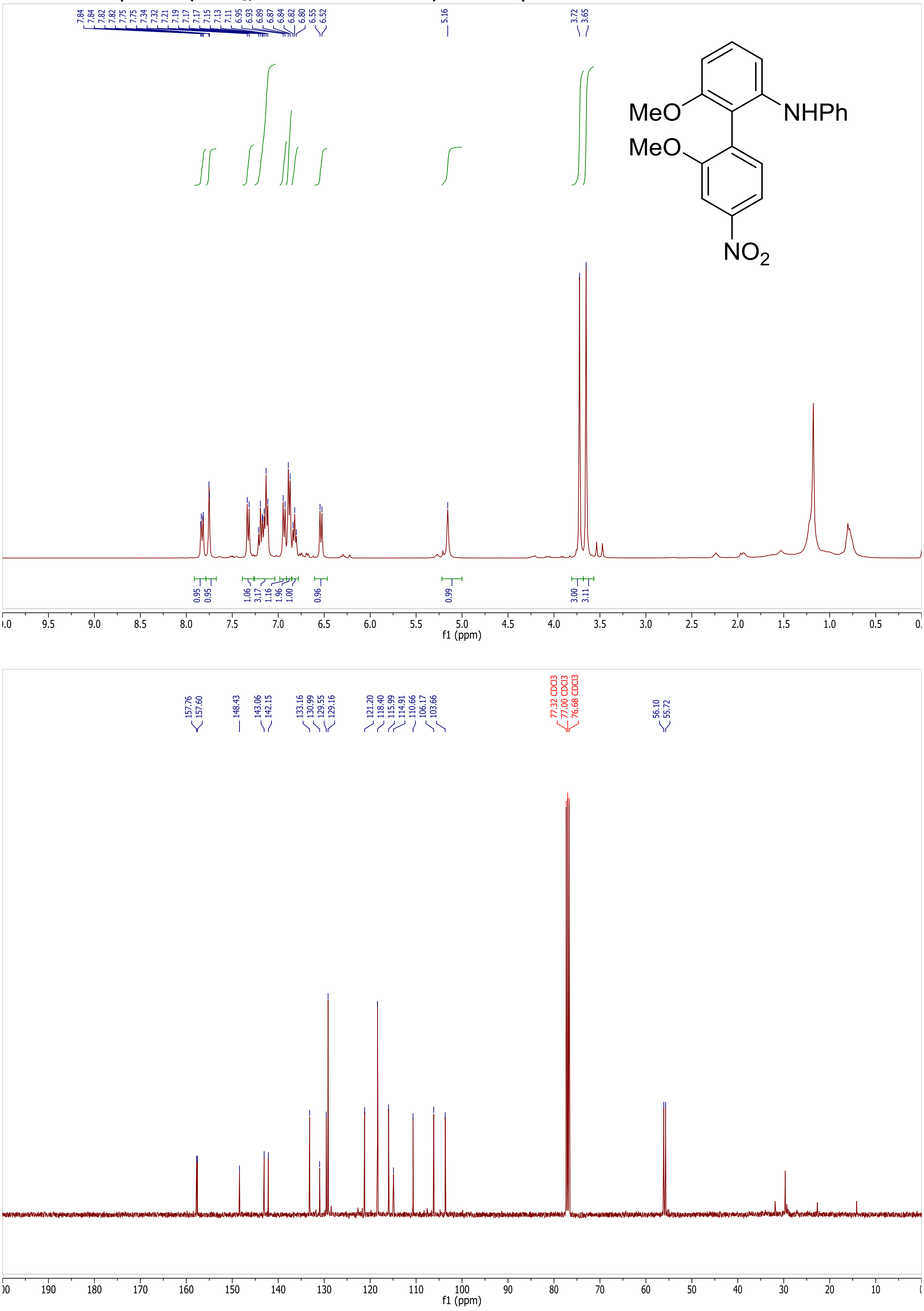

^{1}H and ^{13}C Spectra ($CDCl_3$, 400 and 101 MHz) for compound 4e'

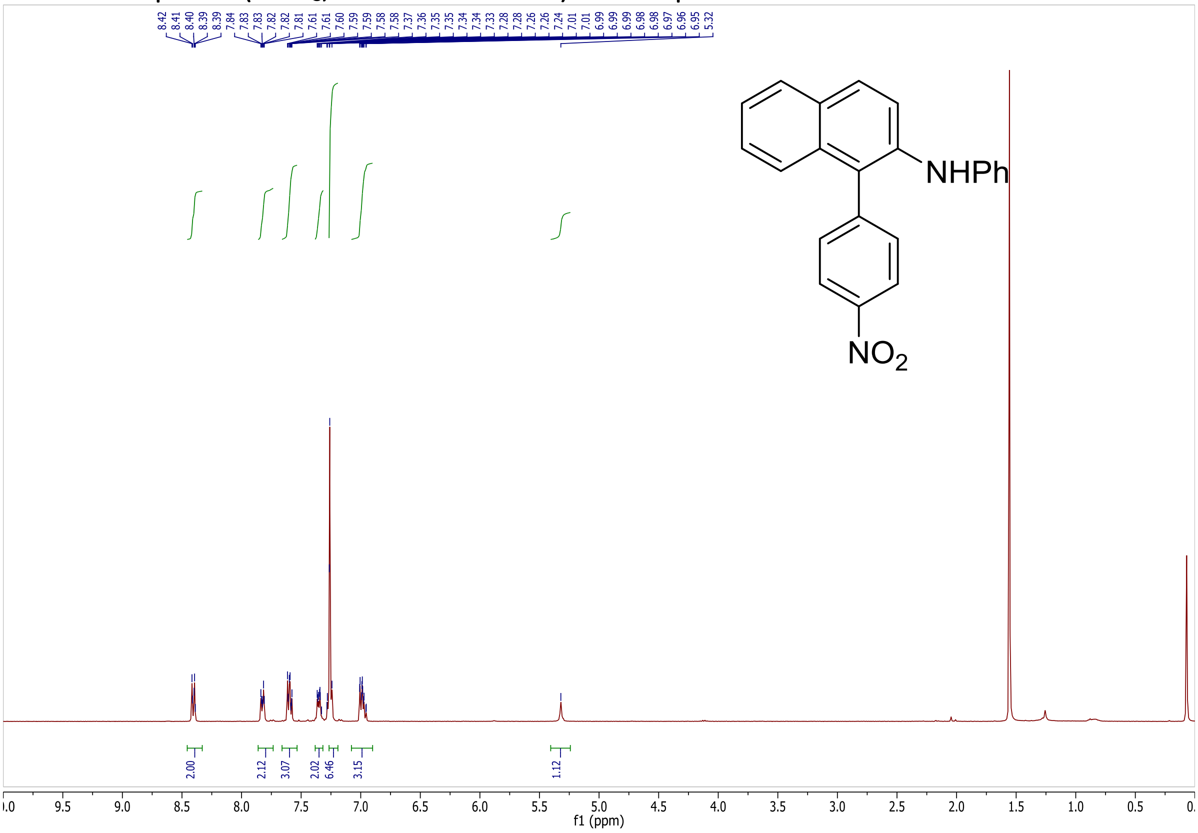

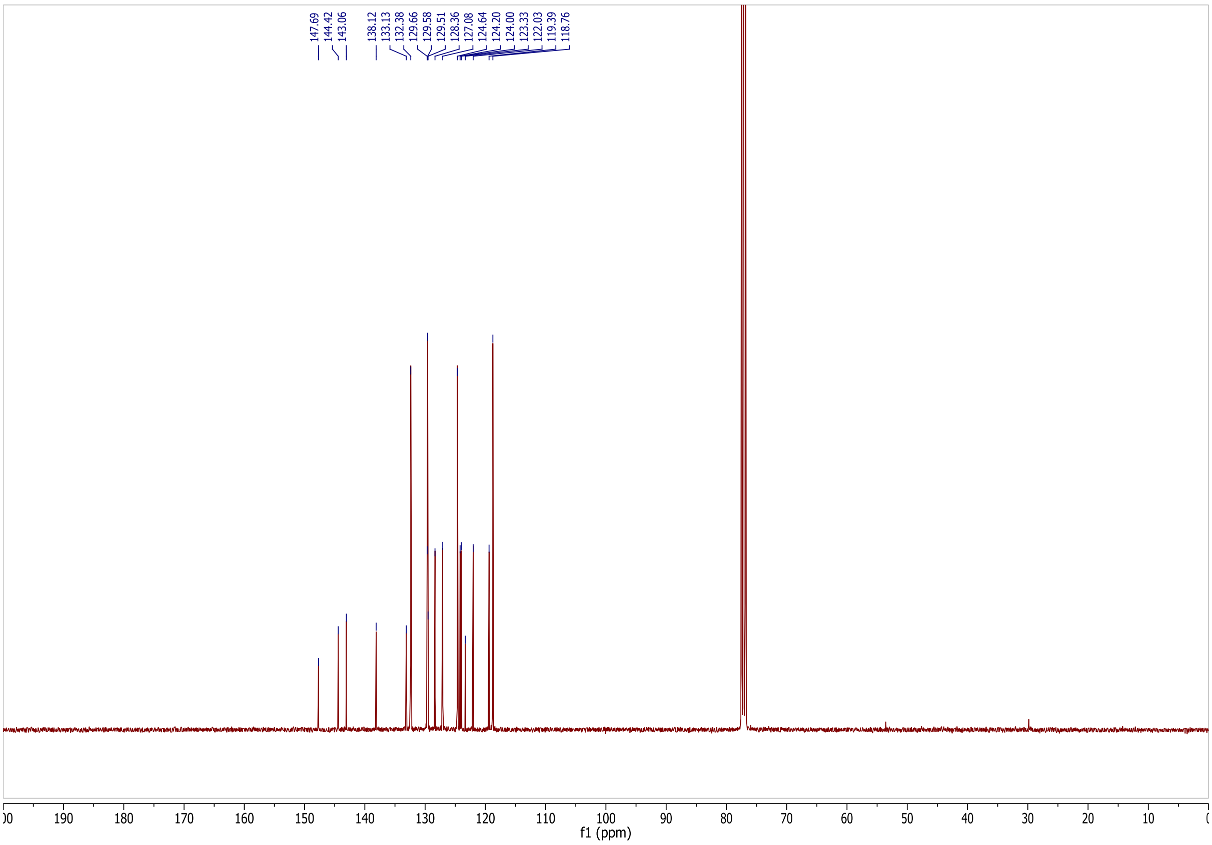

[1]H and [13]C Spectra ($CDCl_3$, 400 and 101 MHz) for compound 4e''

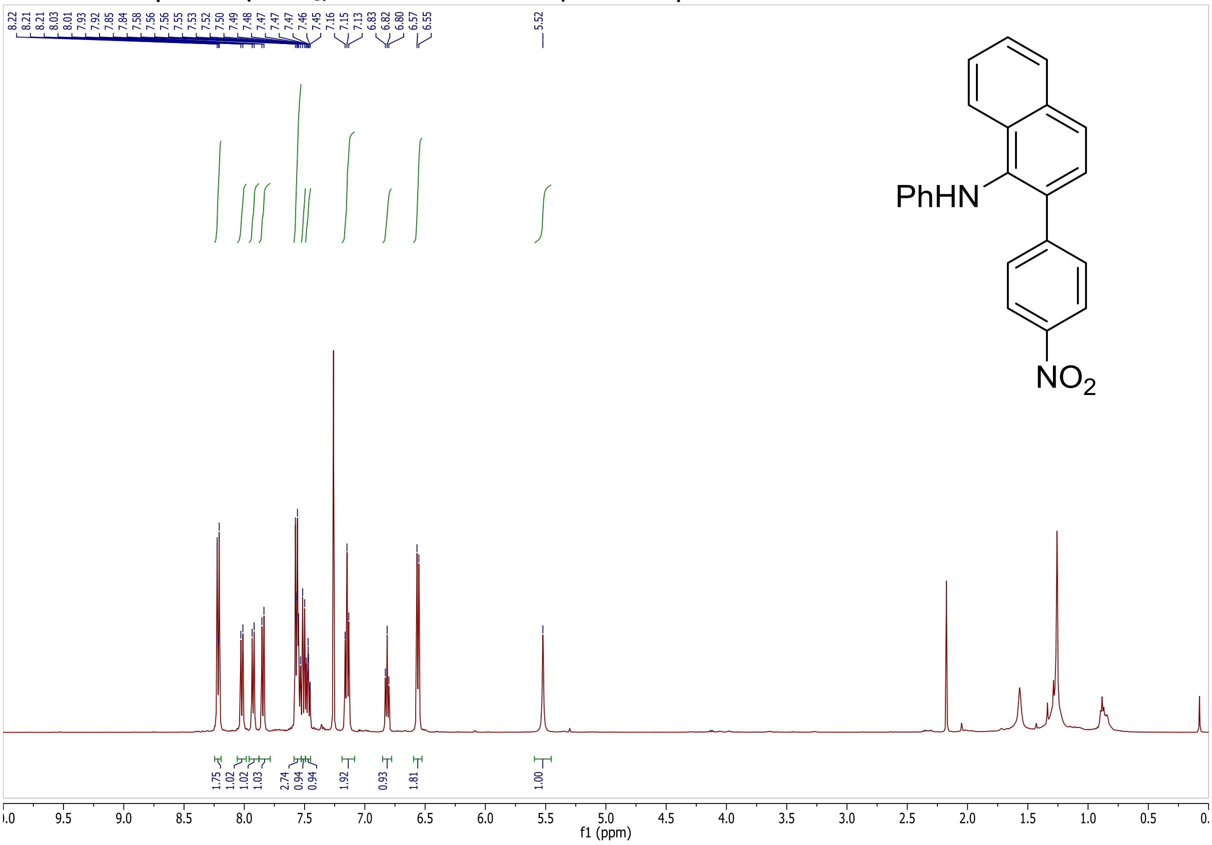

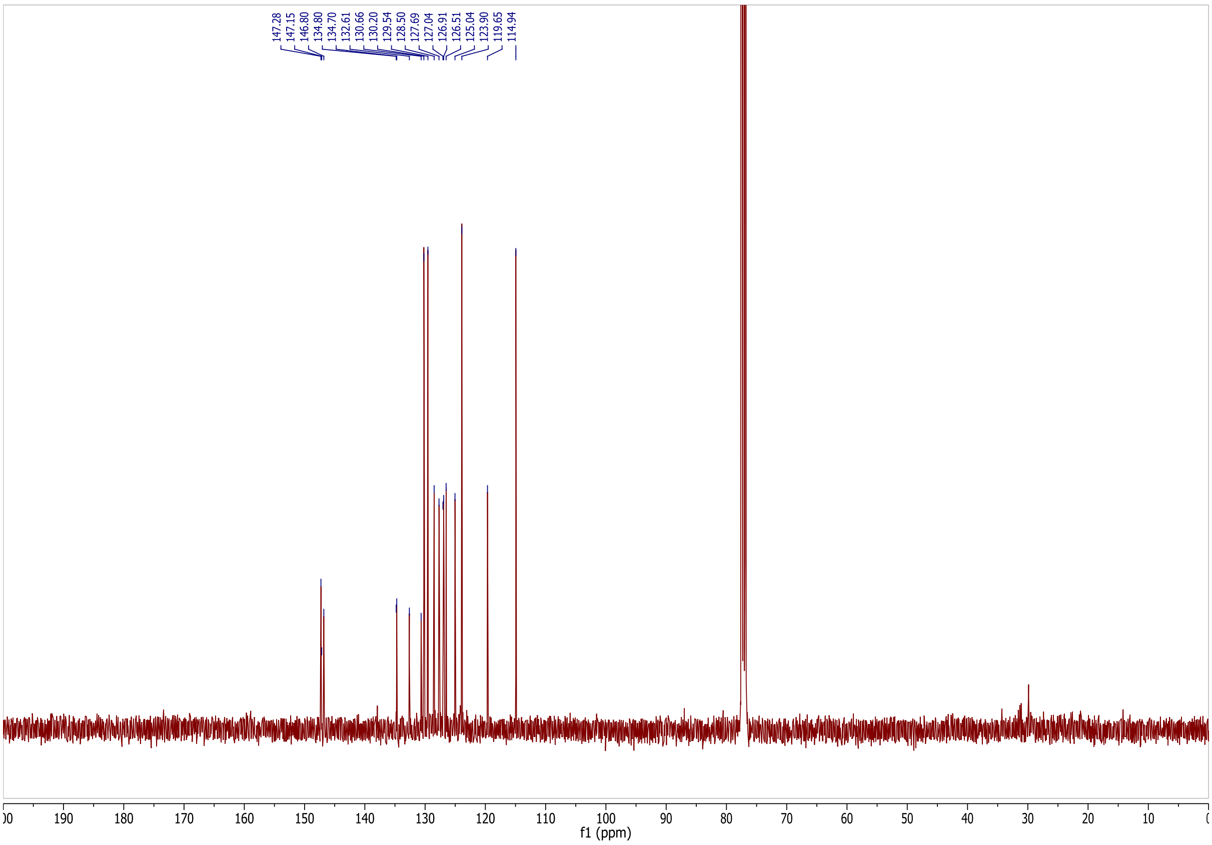

[1]H and [13]C Spectra ($CDCl_3$, 400 and 101 MHz) for compounds 4f

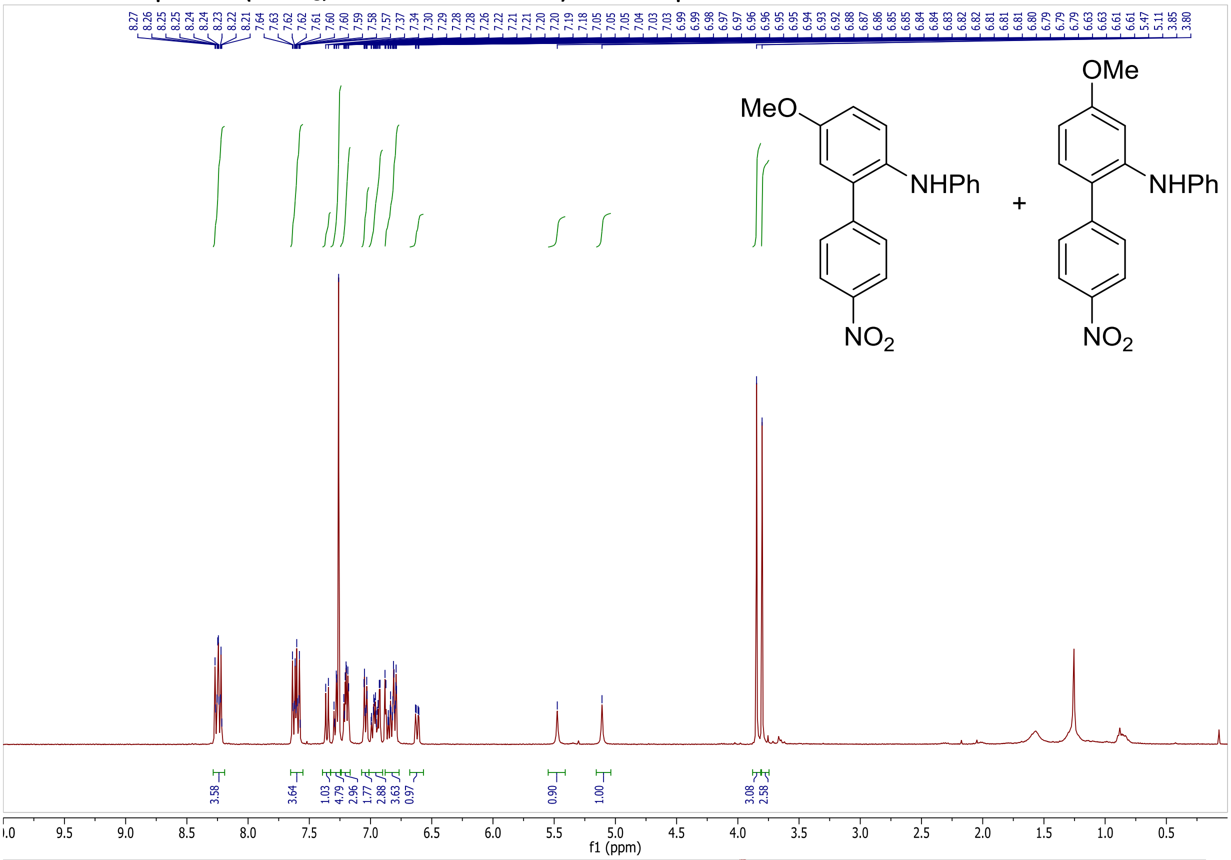

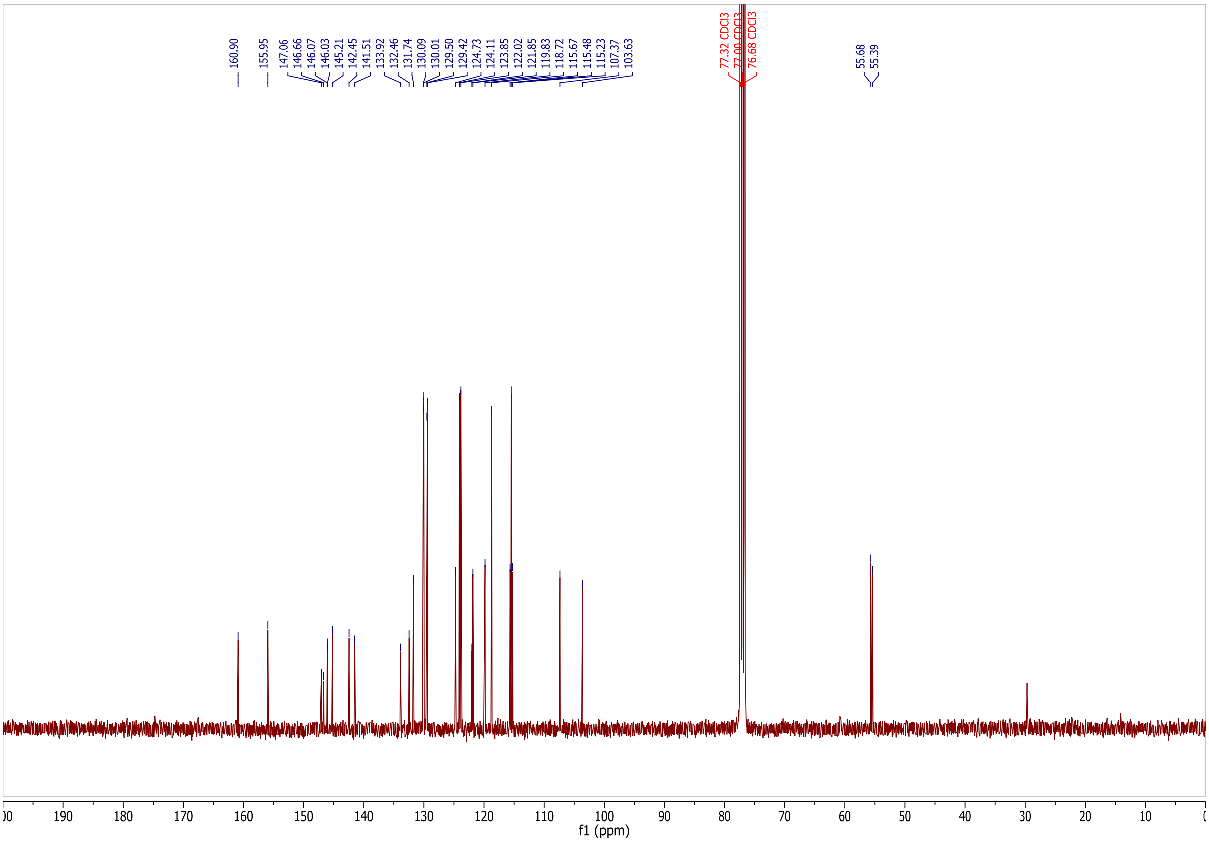

[1]H and [13]C Spectra ($CDCl_3$, 400 and 101 MHz) for compounds 4g

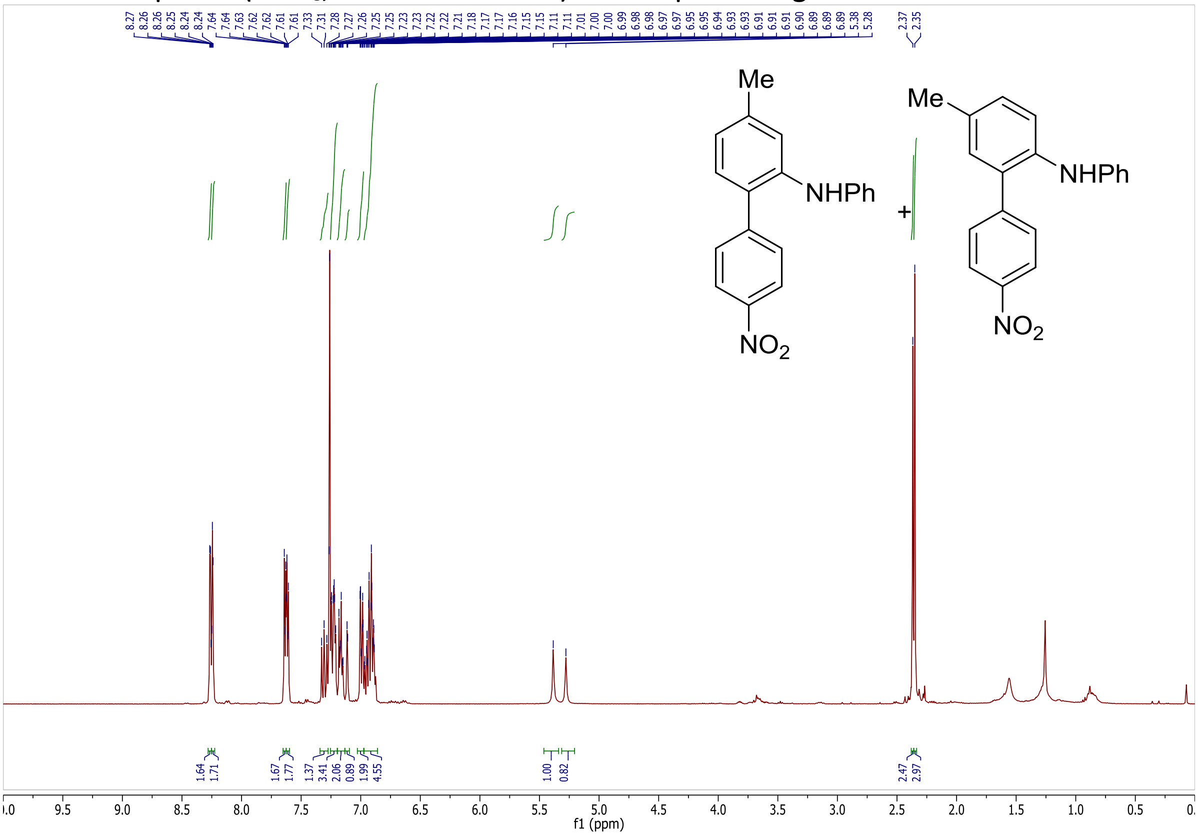

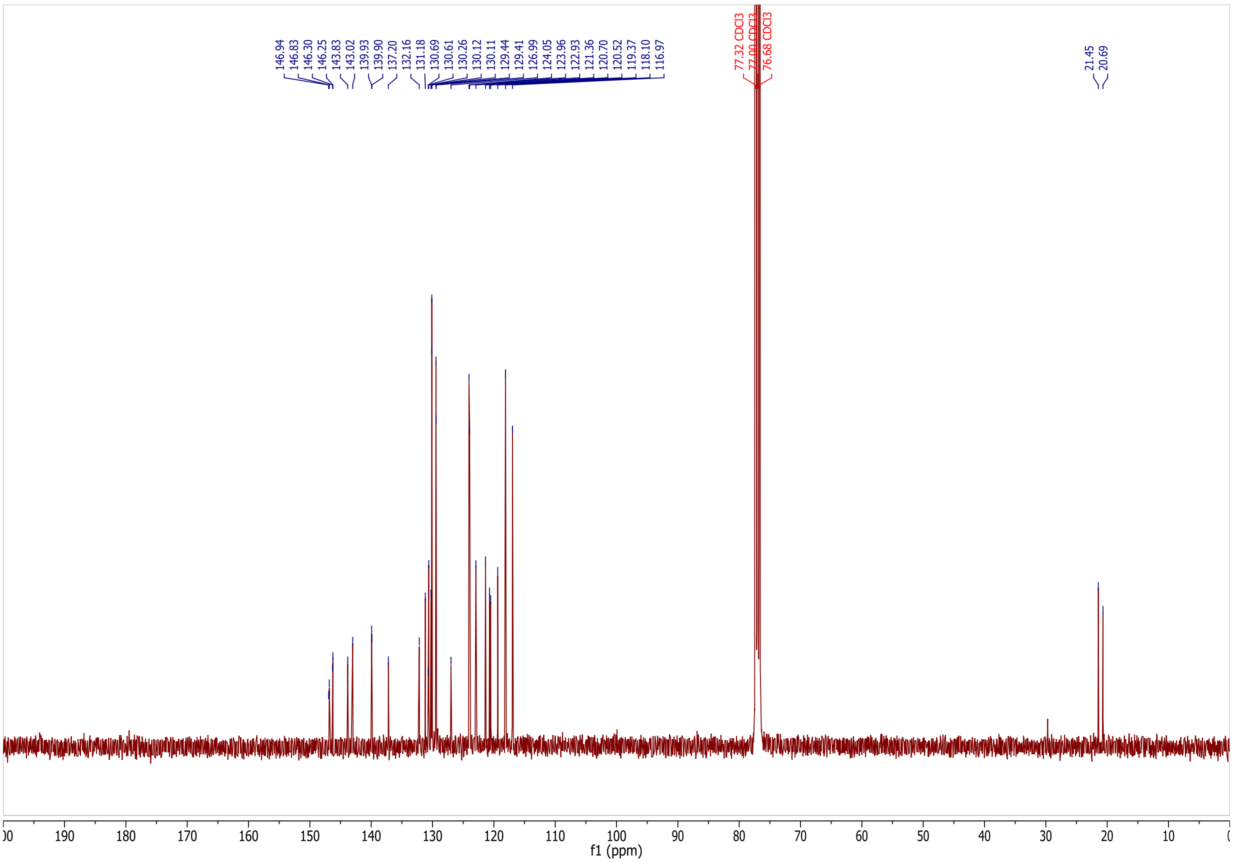

^{1}H and ^{13}C Spectra ($CDCl_3$, 400 and 101 MHz) for compounds 4i

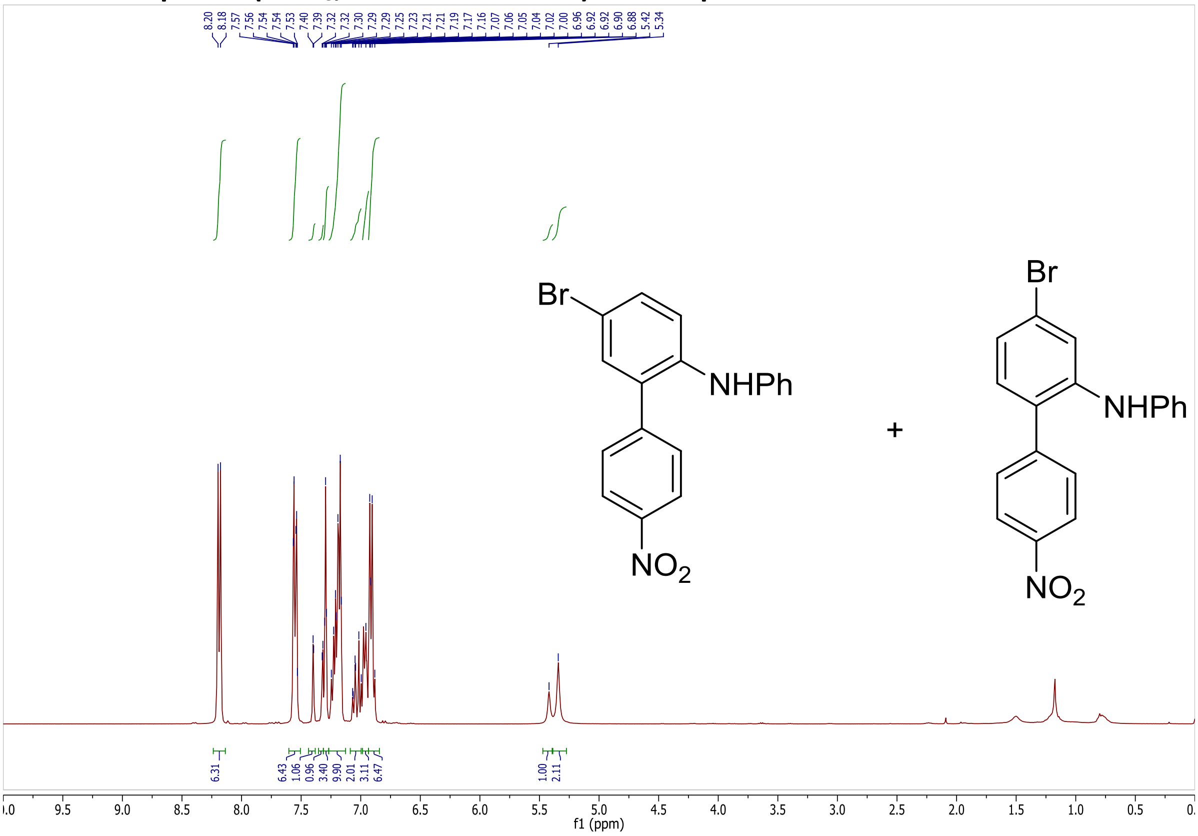

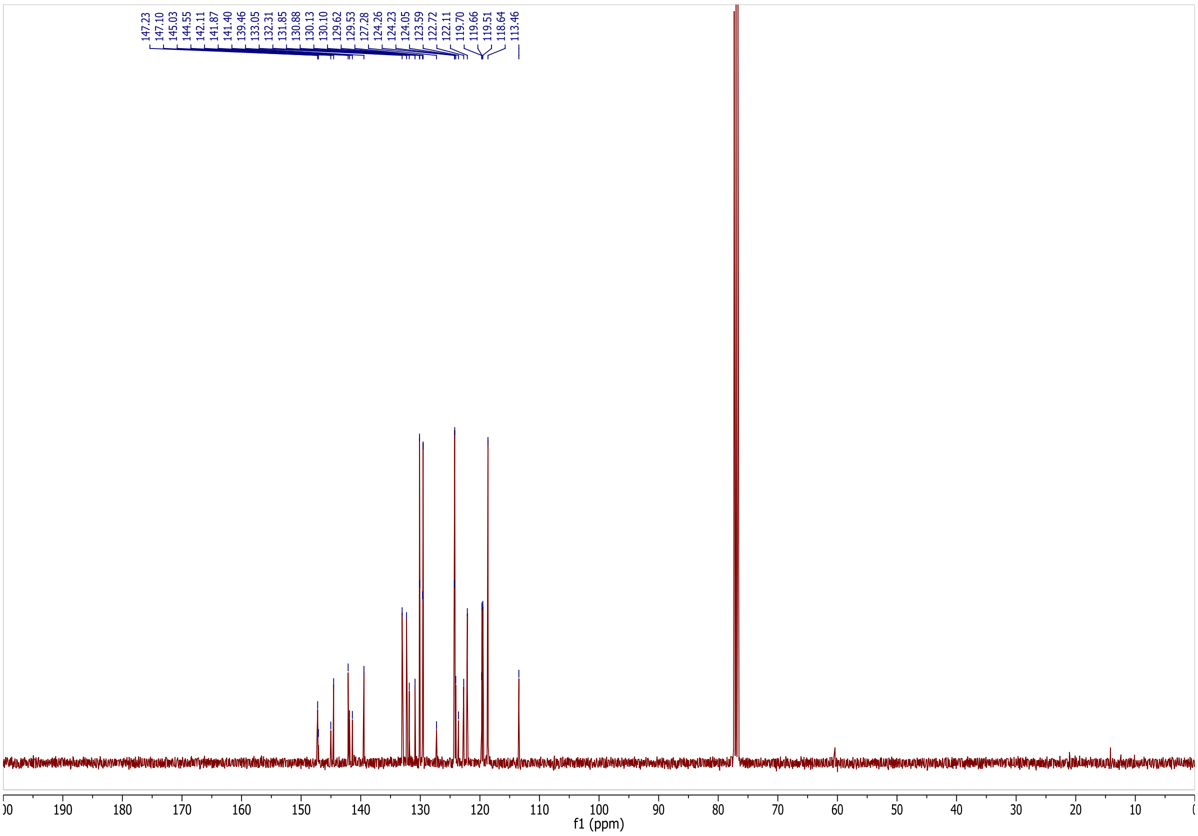

^{1}H and ^{13}C Spectra ($CDCl_3$, 400 and 101 MHz) for compound 4j

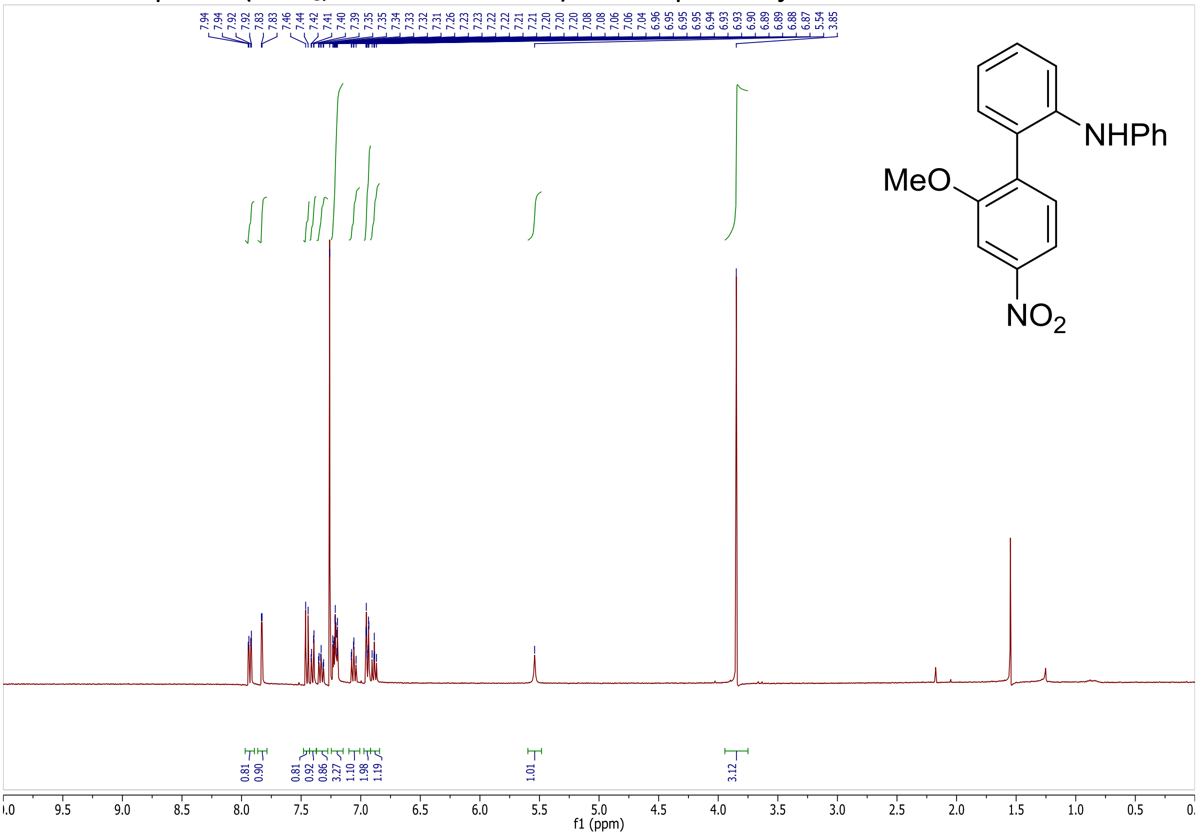

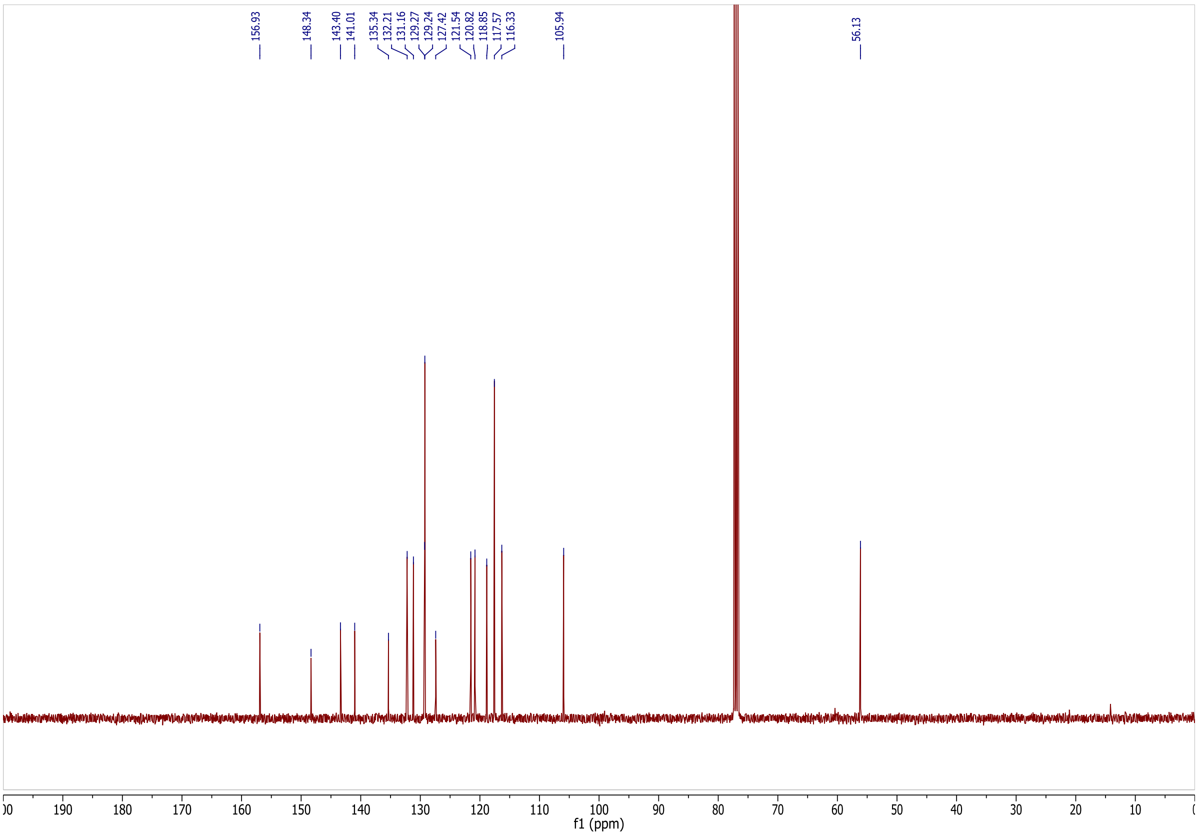

[1]H and [13]C Spectra ($CDCl_3$, 400 and 101 MHz) for compound 4k

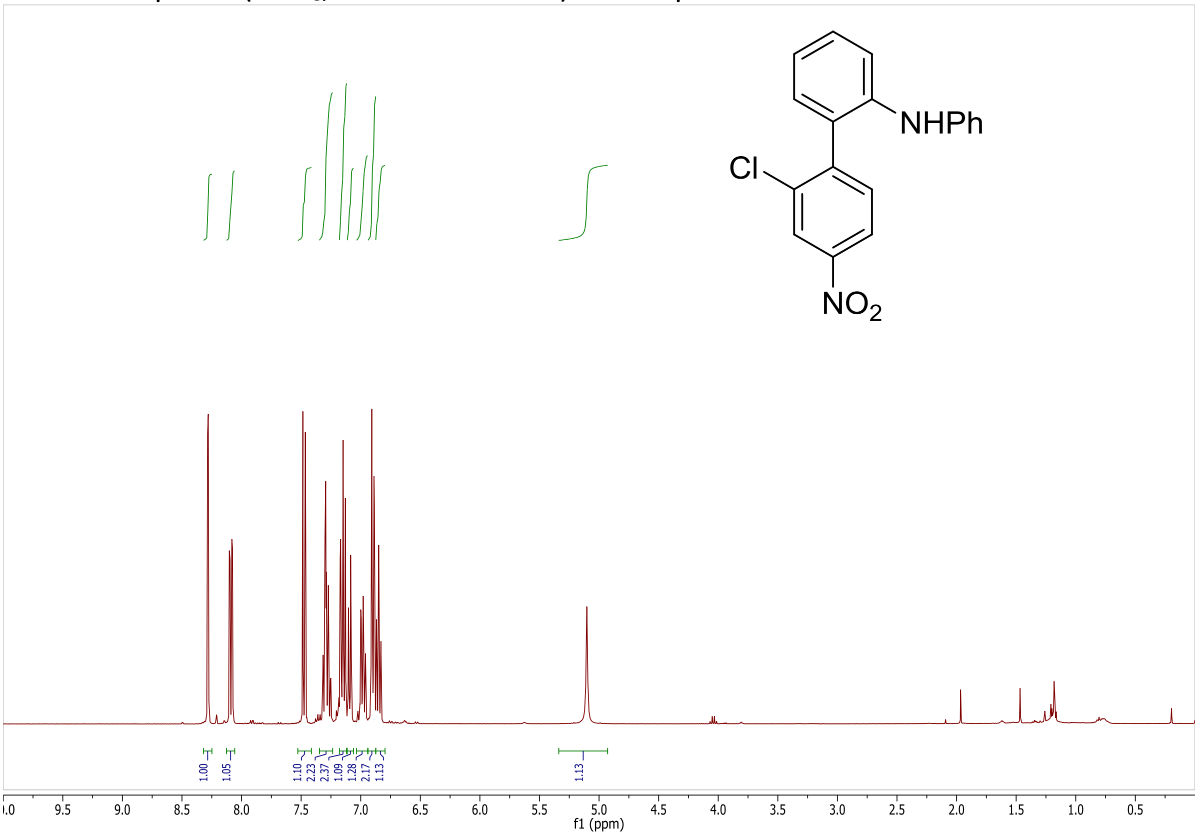

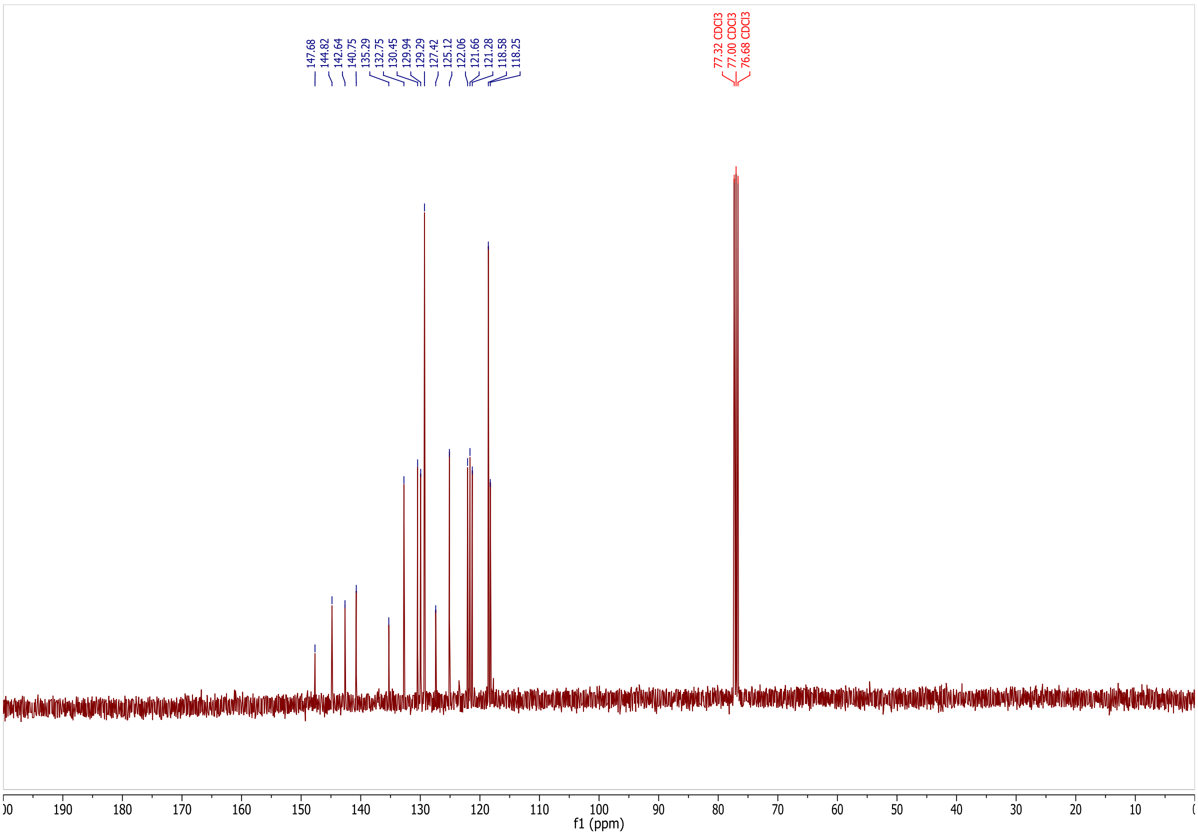

[1]H and [13]C Spectra (DMSO-*d6*, 400 and 101 MHz) for compound 4l

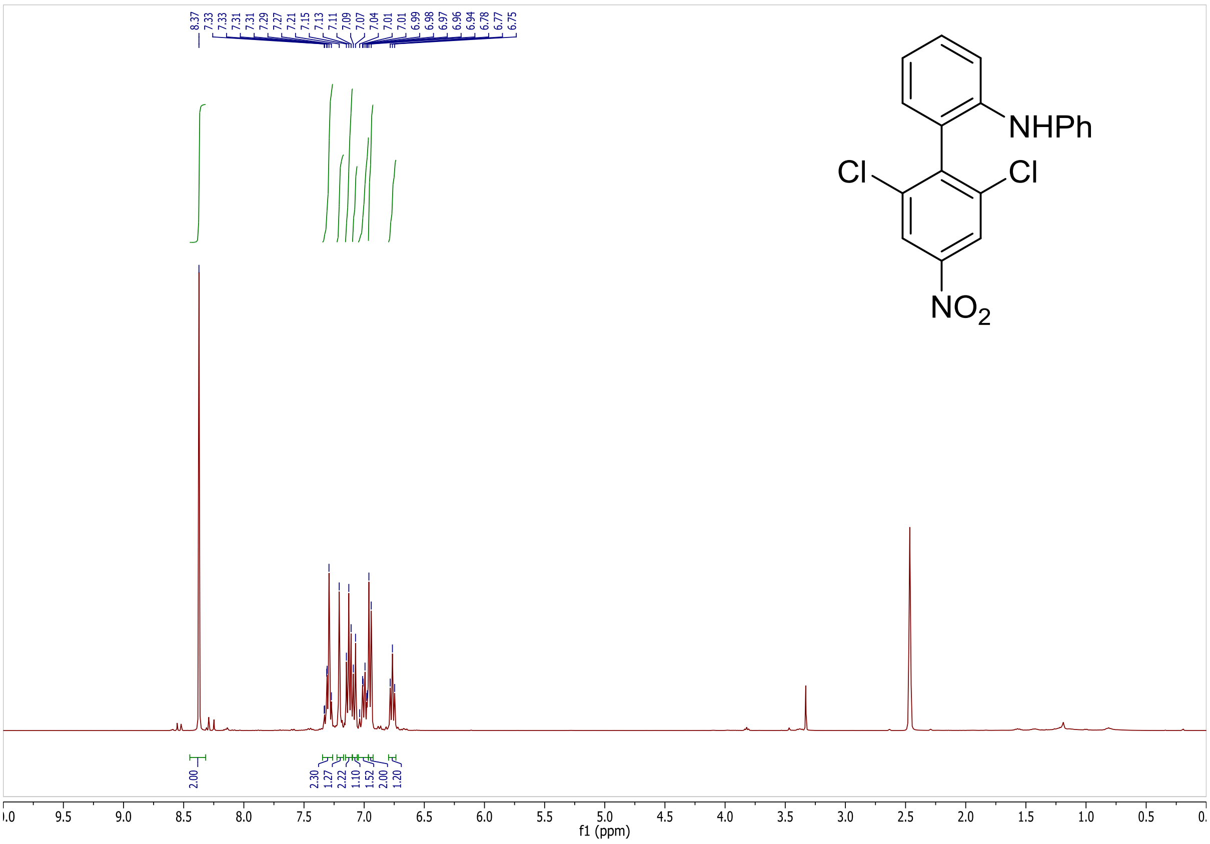

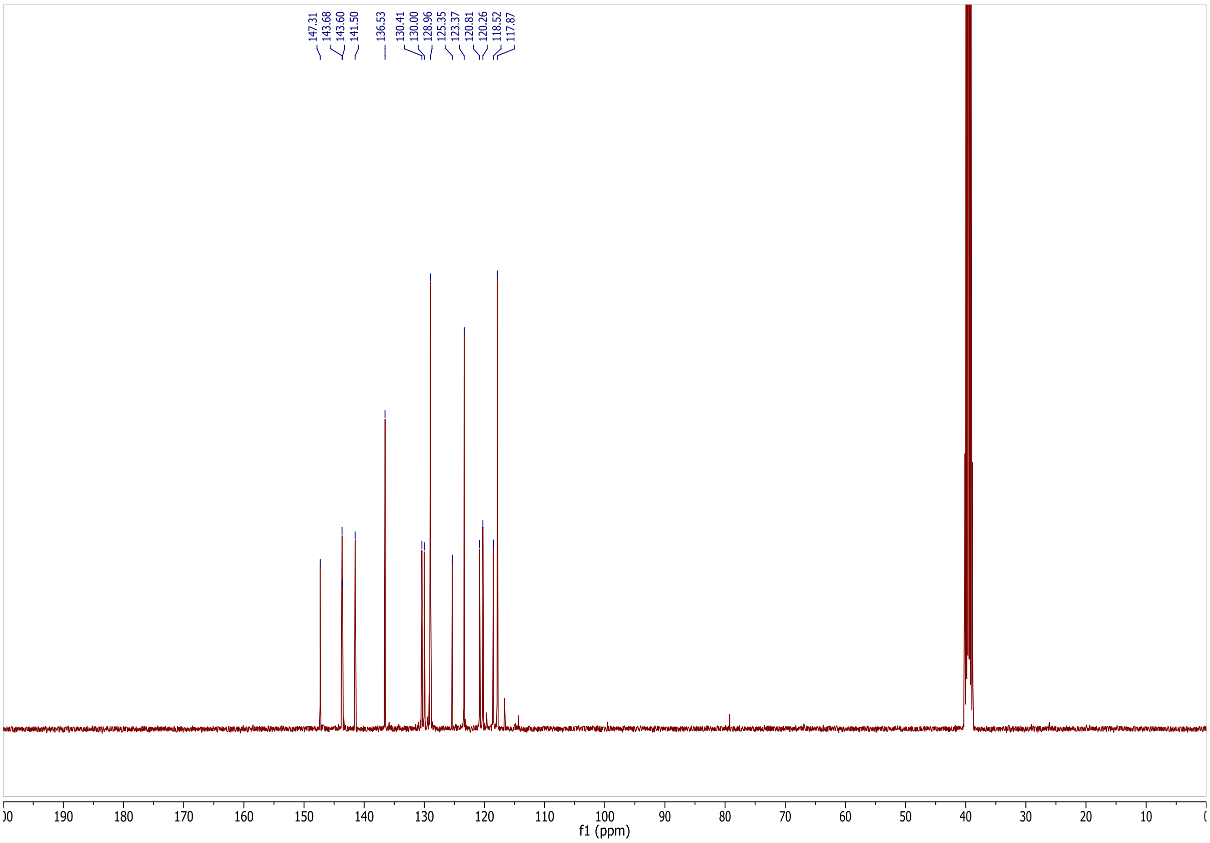

^{1}H and ^{13}C Spectra ($CDCl_3$, 400 and 126 MHz) for compound 4m

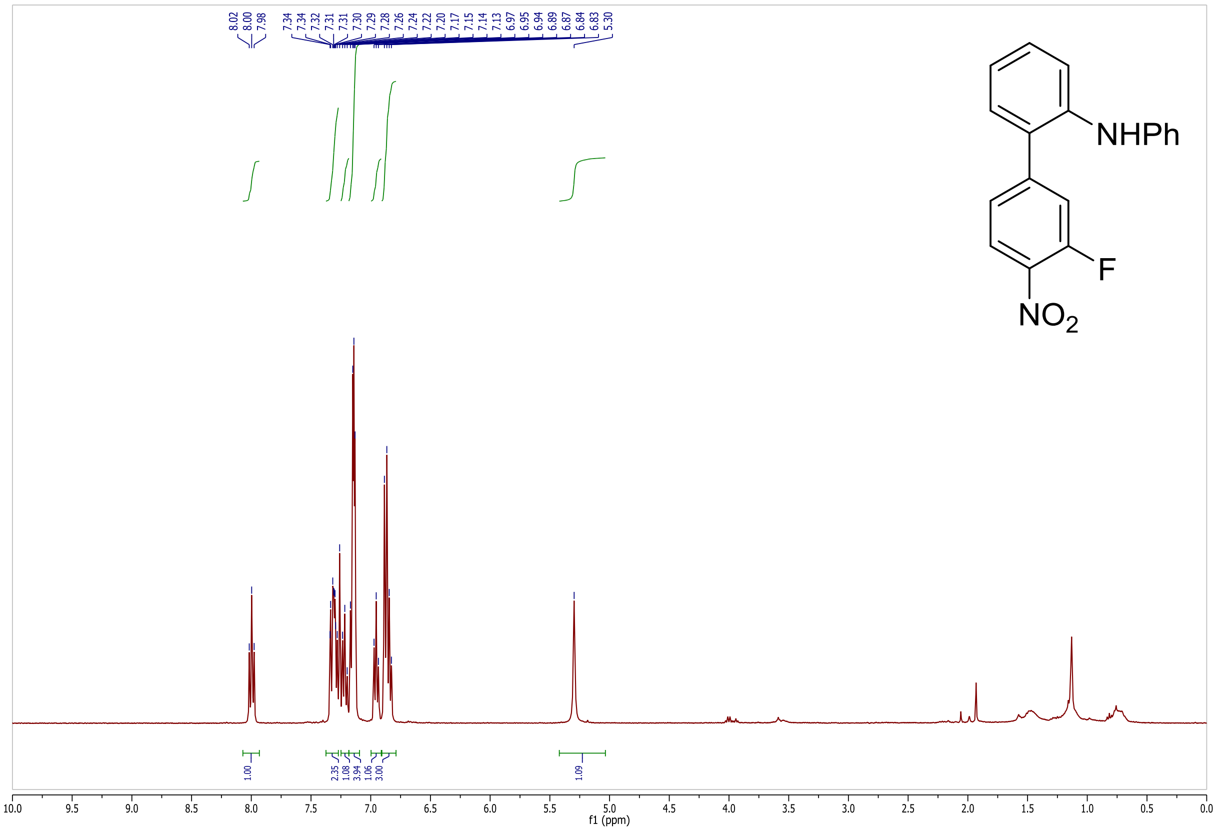

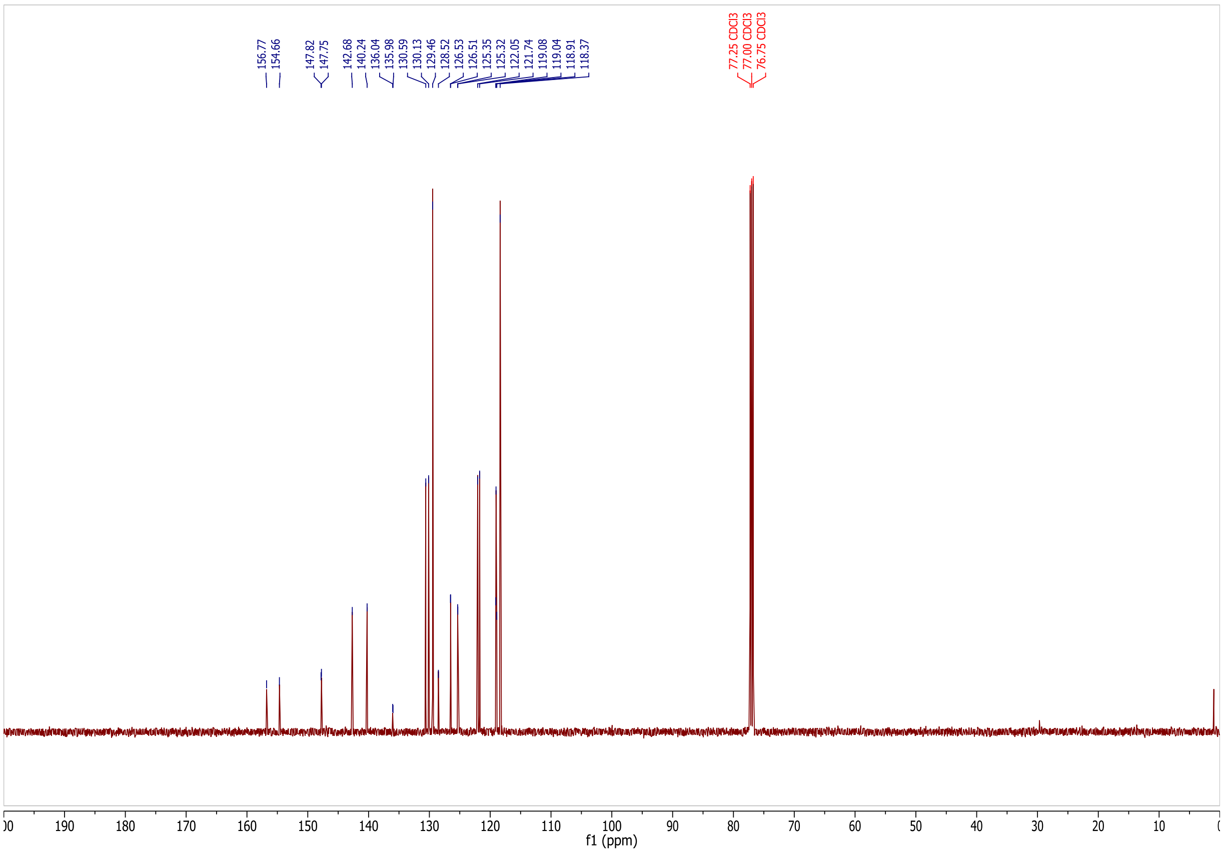

[1]H and [13]C Spectra ($CDCl_3$, 400 and 101 MHz) for compound 4n

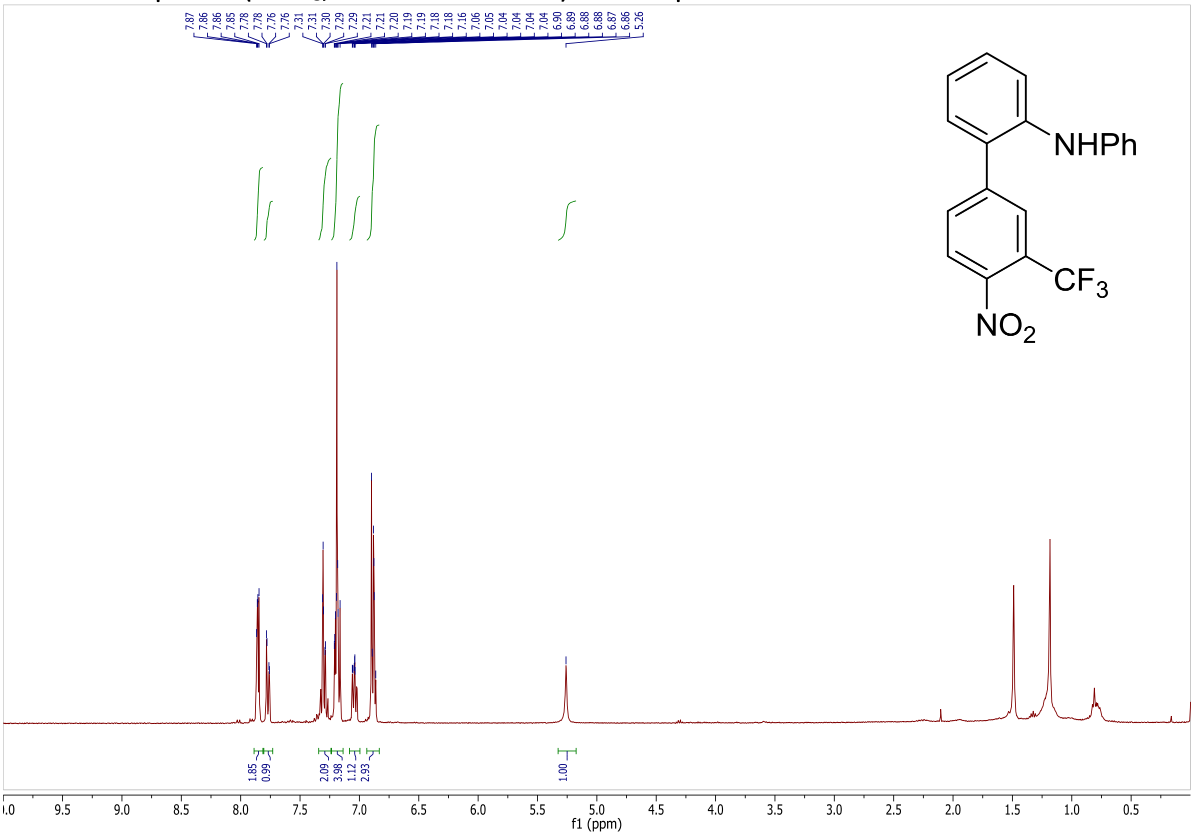

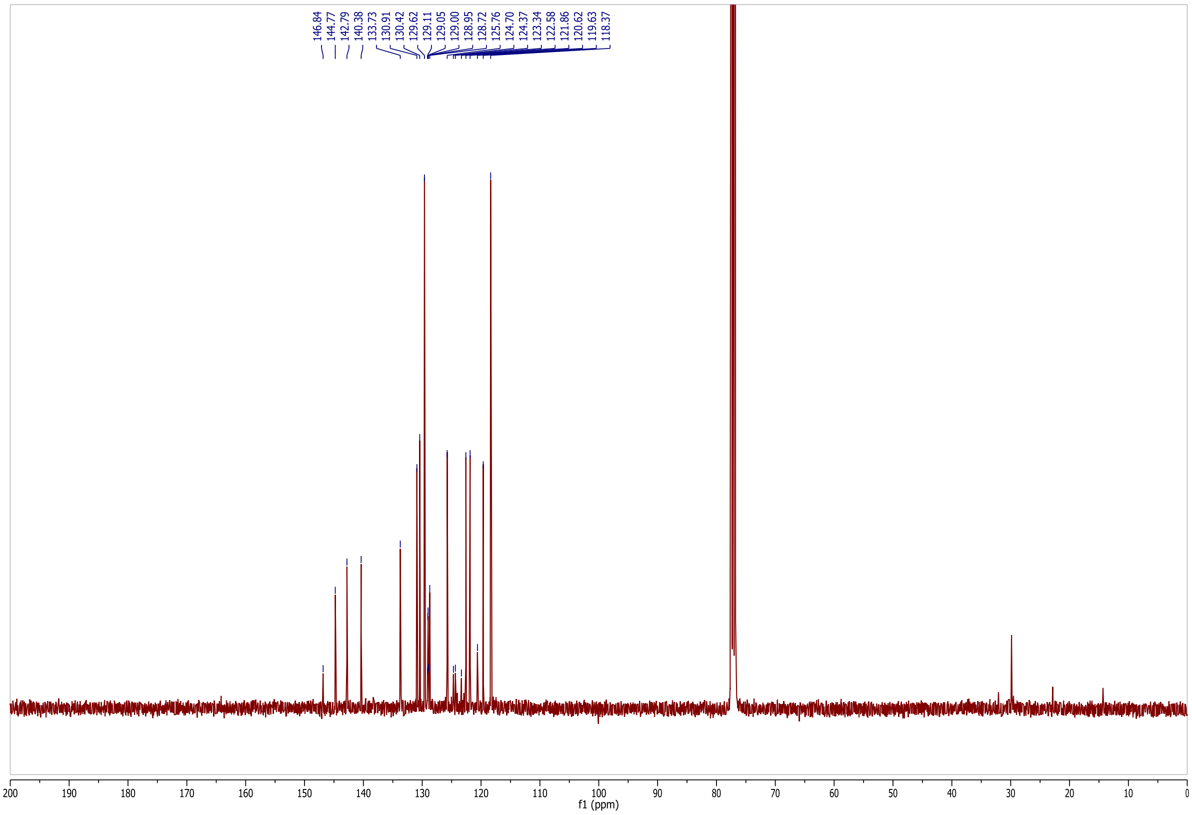

^{1}H and ^{13}C Spectra ($CDCl_3$, 500 and 126 MHz) for compound 4o

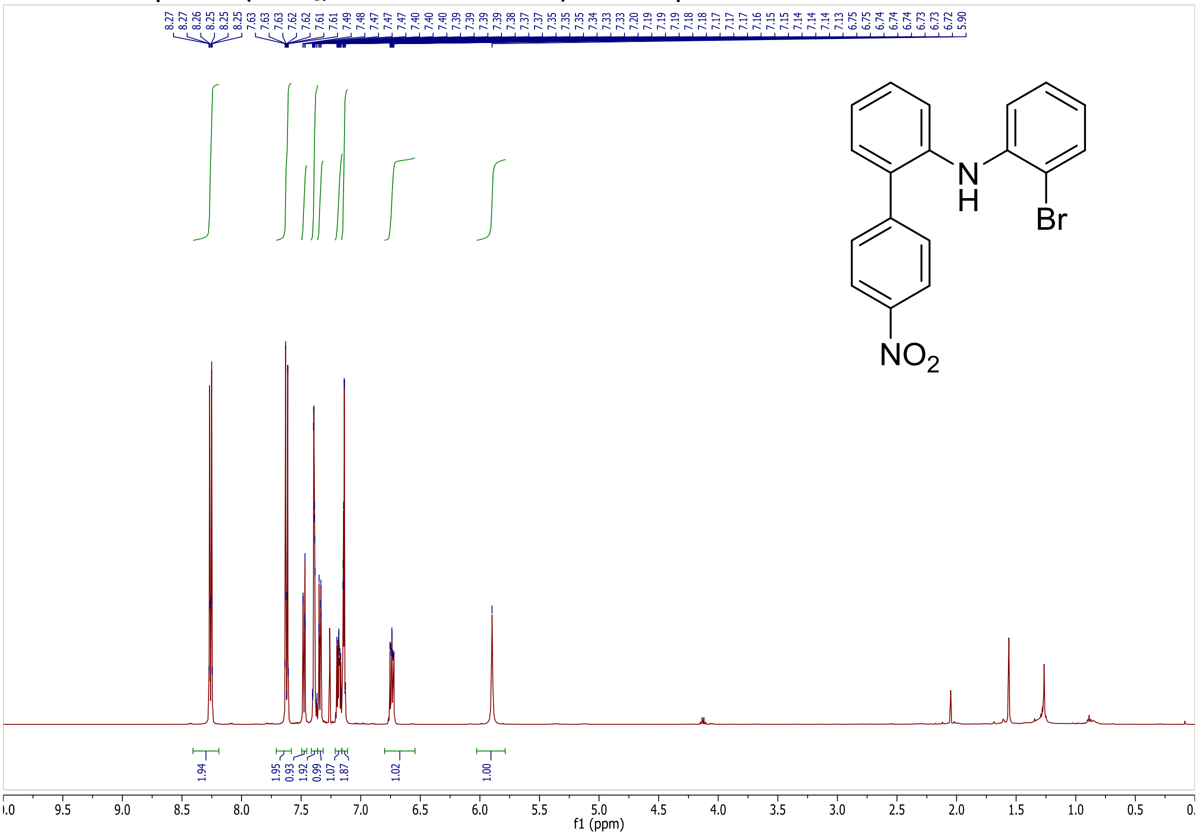

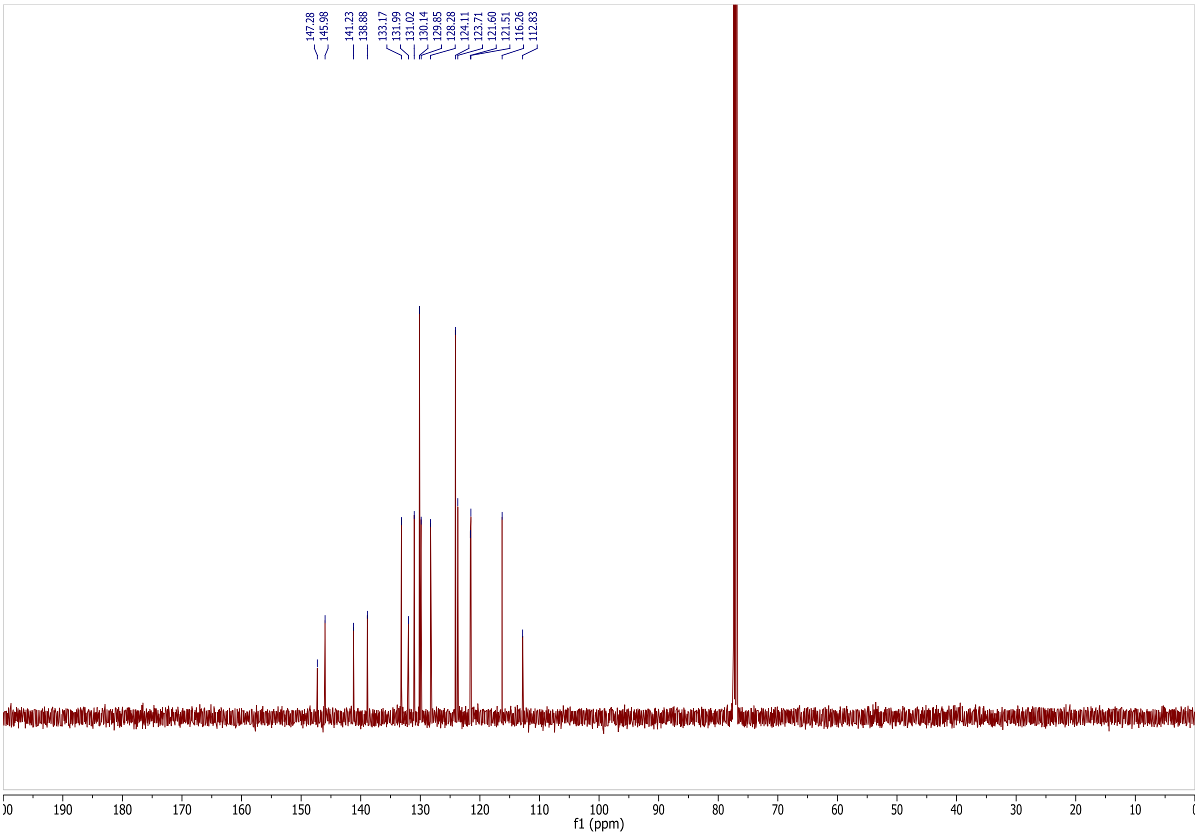

[1]H and [13]C Spectra ($CDCl_3$, 400 and 101 MHz) for compound 4p

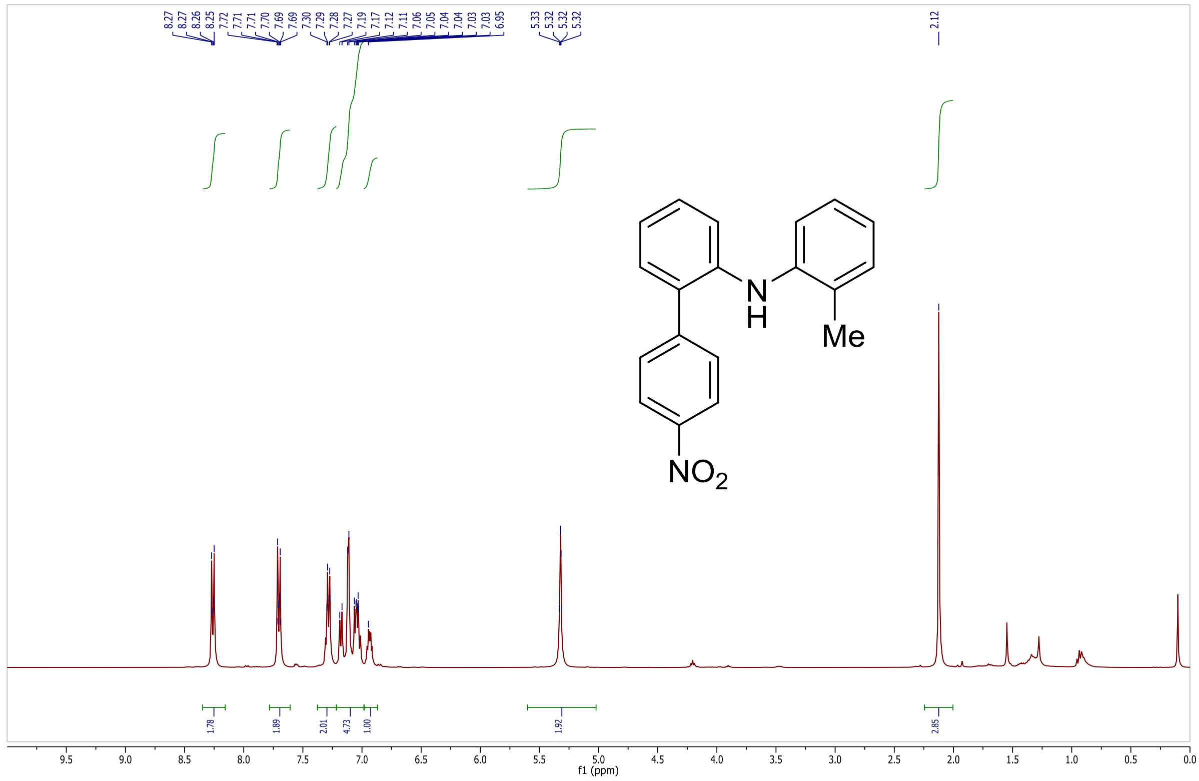

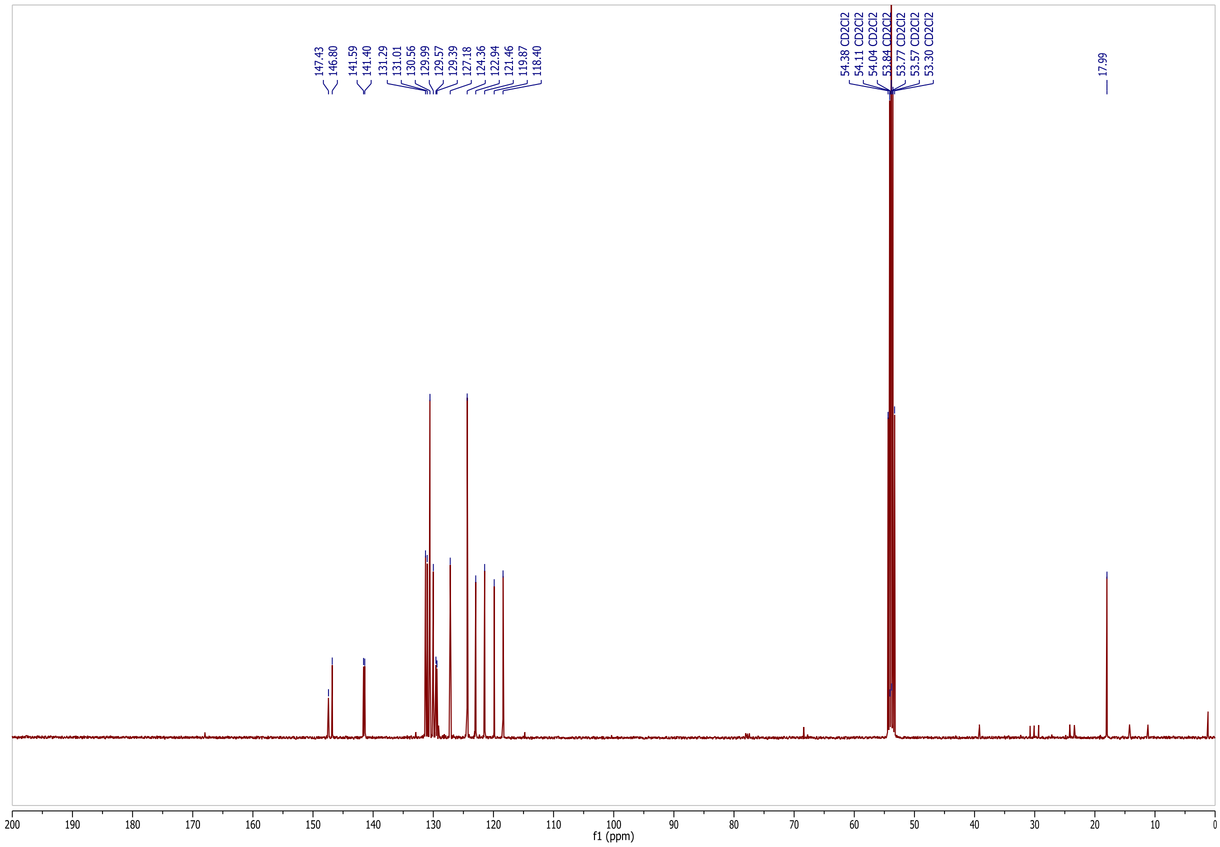

^{1}H and ^{13}C Spectra ($CDCl_3$, 400 and 101 MHz) for compound 4q

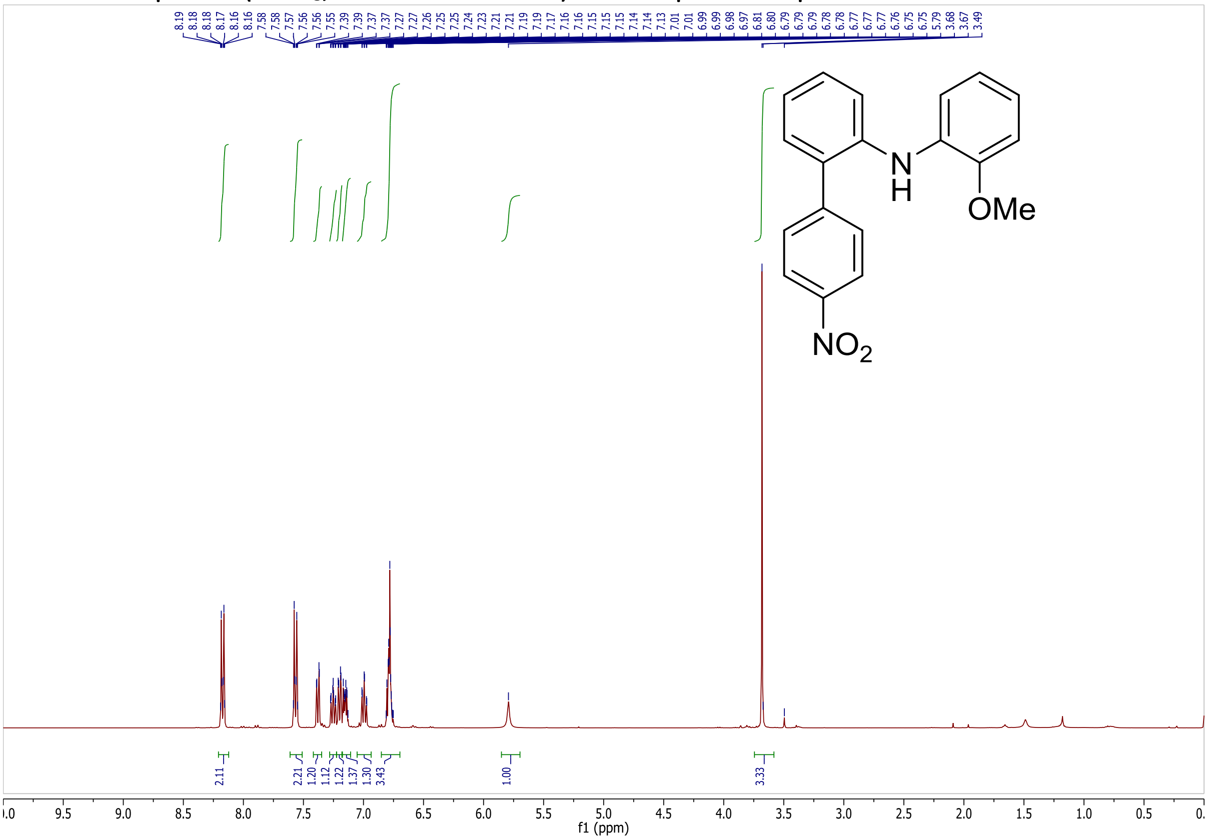

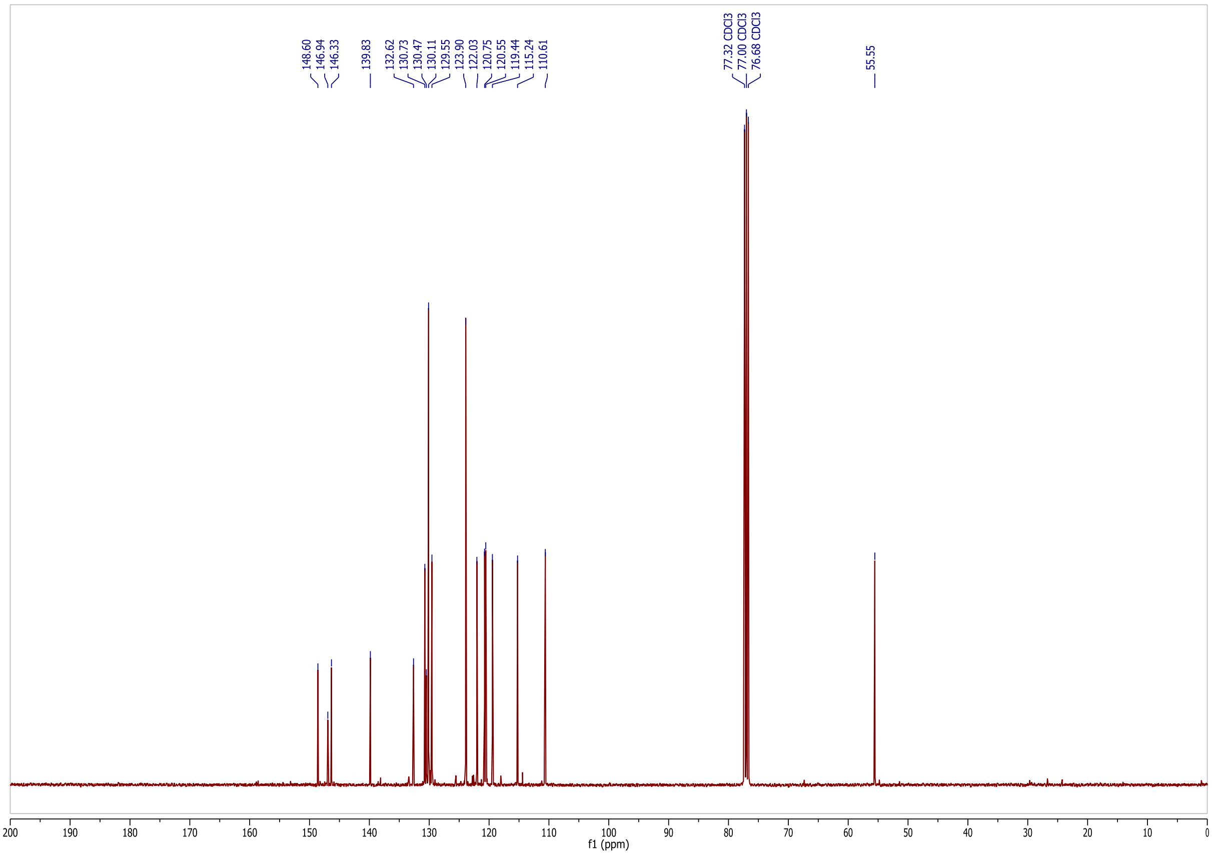

[1]H and [13]C Spectra ($CDCl_3$, 400 and 101 MHz) for compound 4r

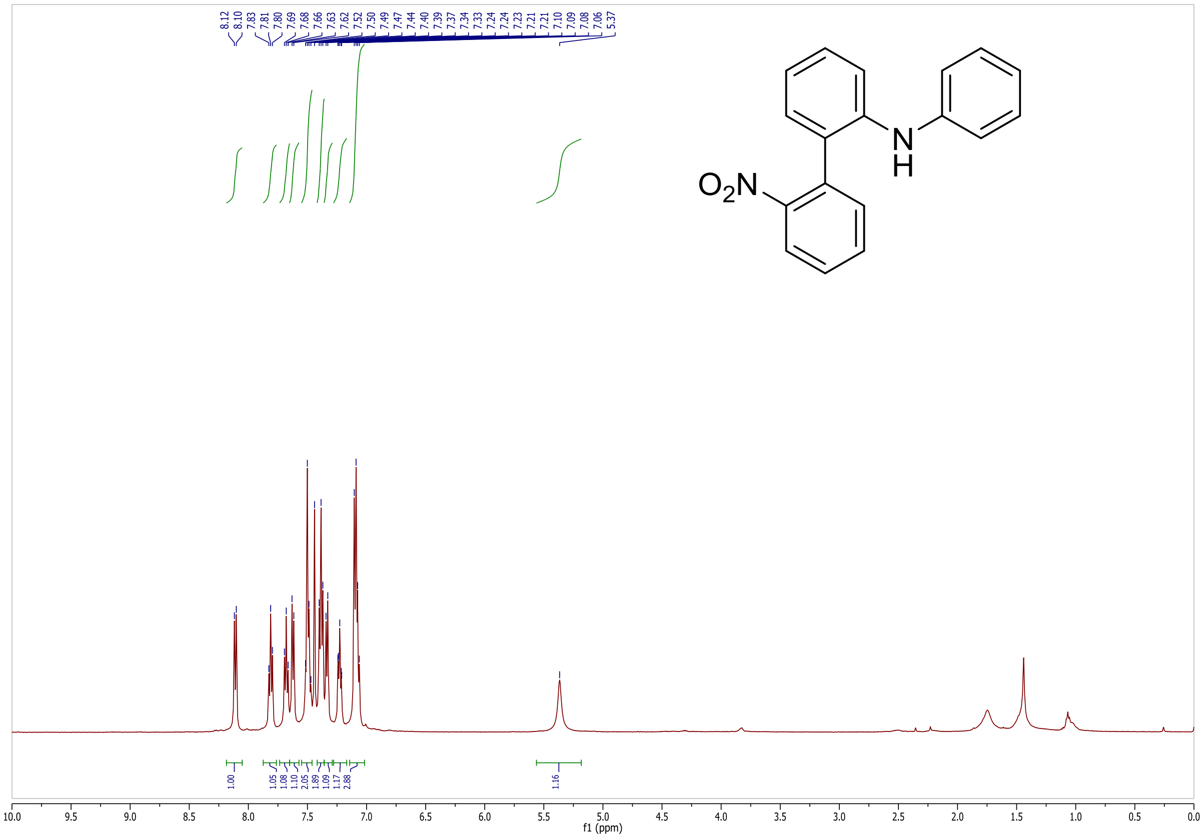

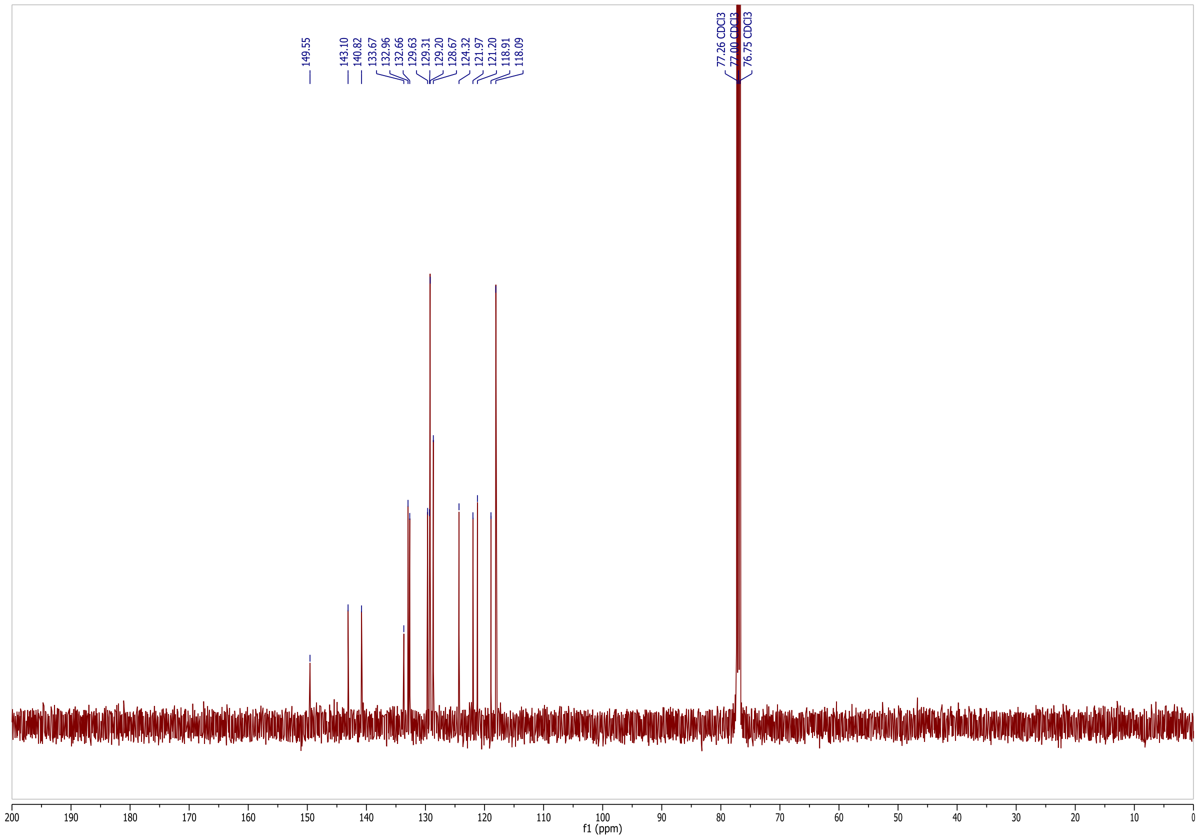

^{1}H and ^{13}C Spectra ($CDCl_3$, 400 and 101 MHz) for compound 4s

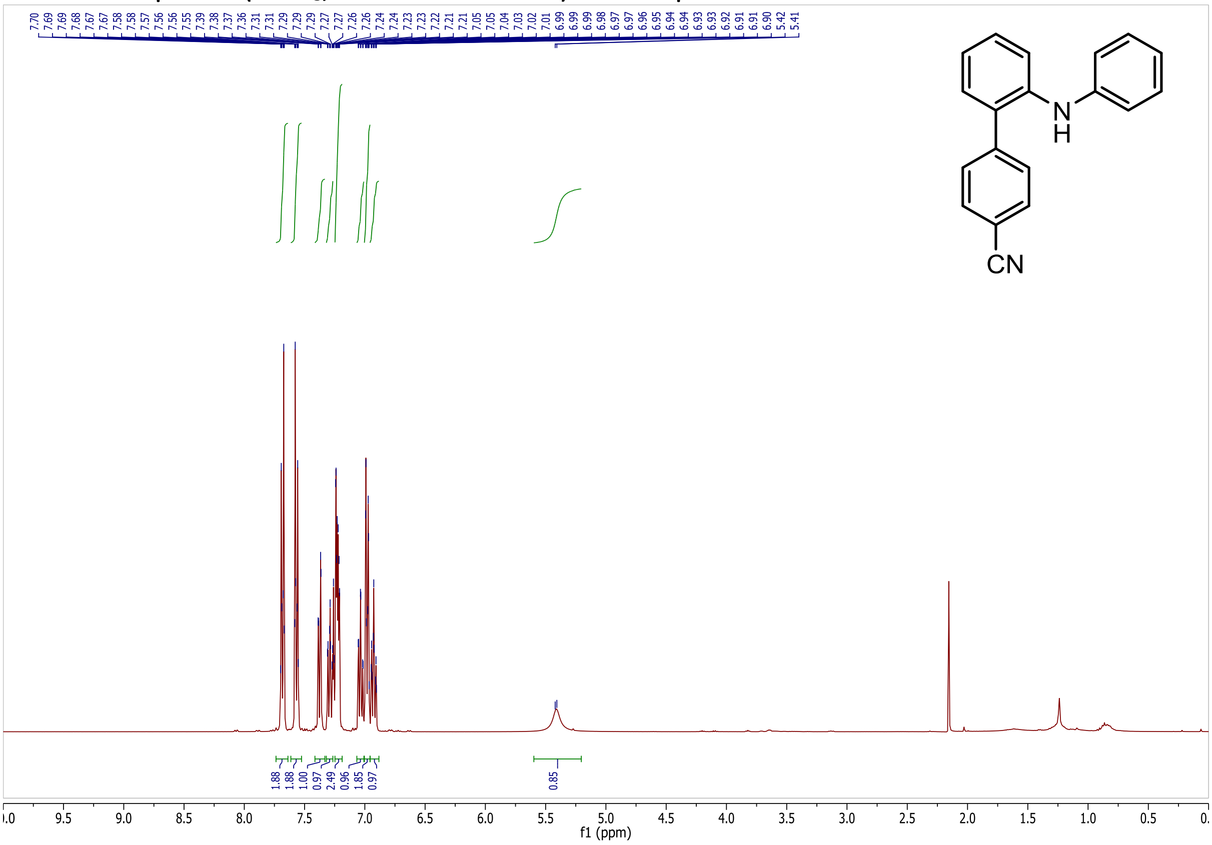

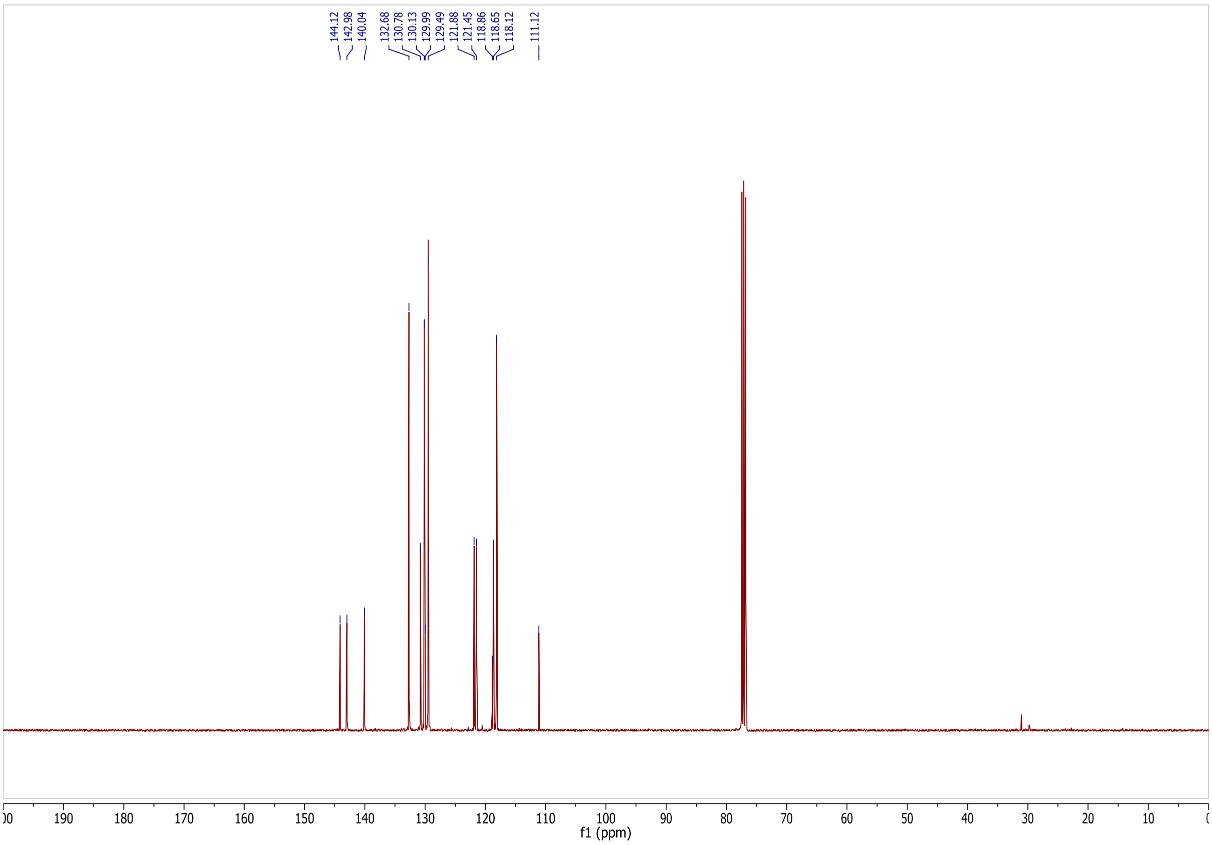

^{1}H and ^{13}C Spectra ($CDCl_3$, 400 and 101 MHz) for compound 4t

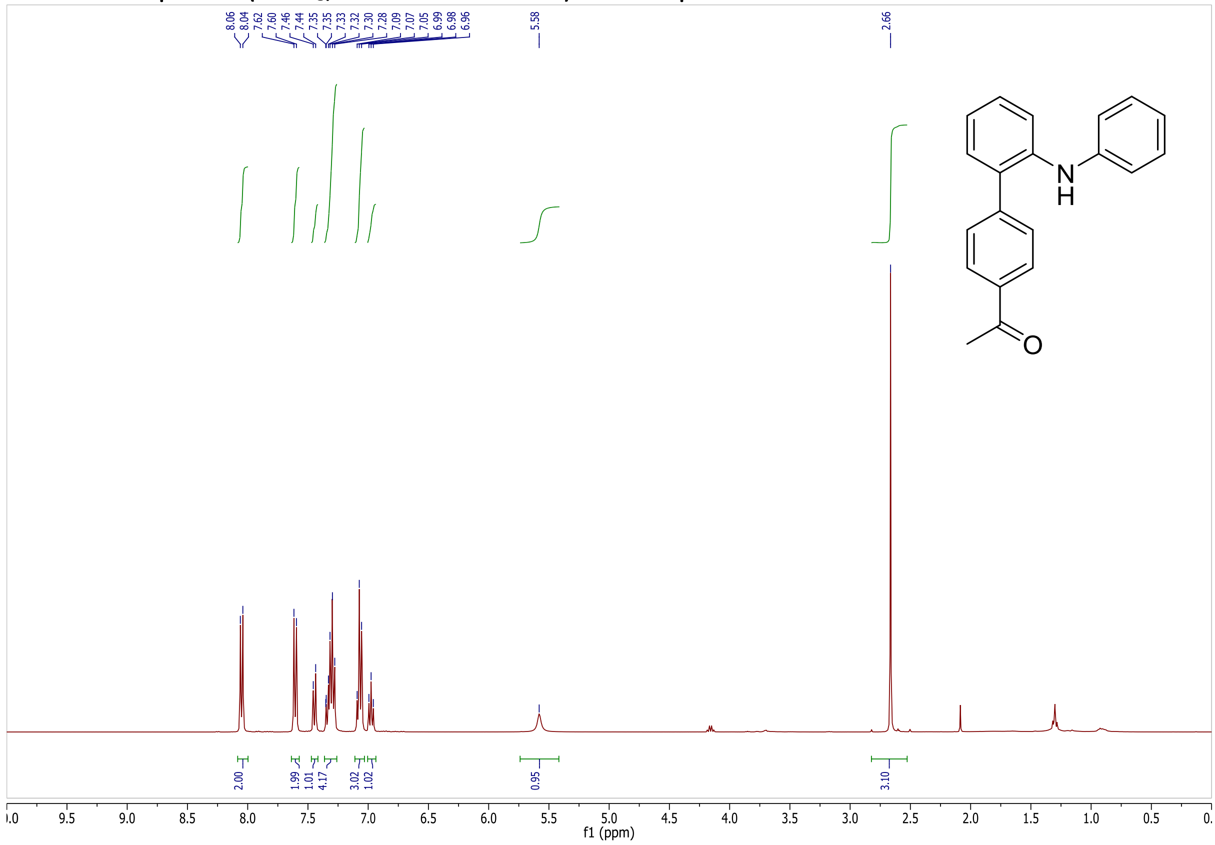

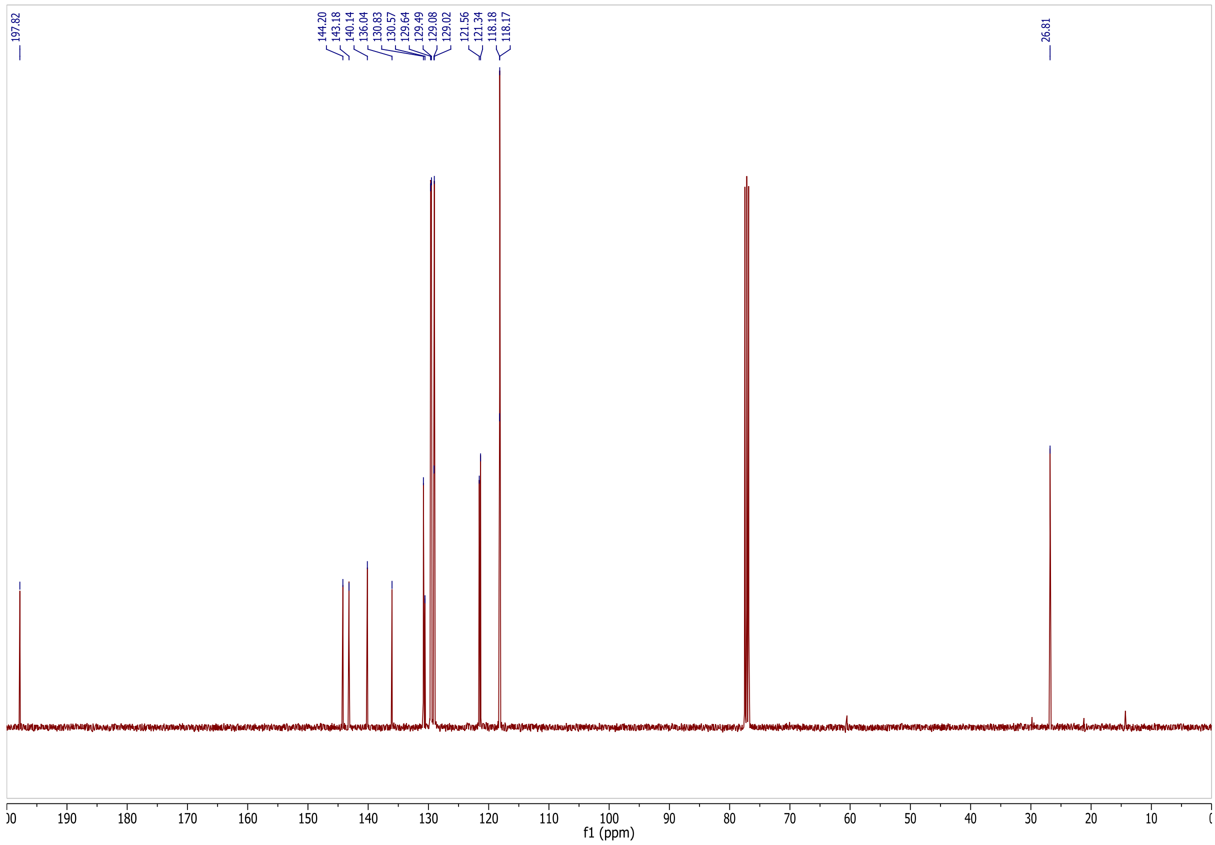

^1H and ^{13}C Spectra ($CDCl_3$, 500 and 126 MHz) for compound 4u

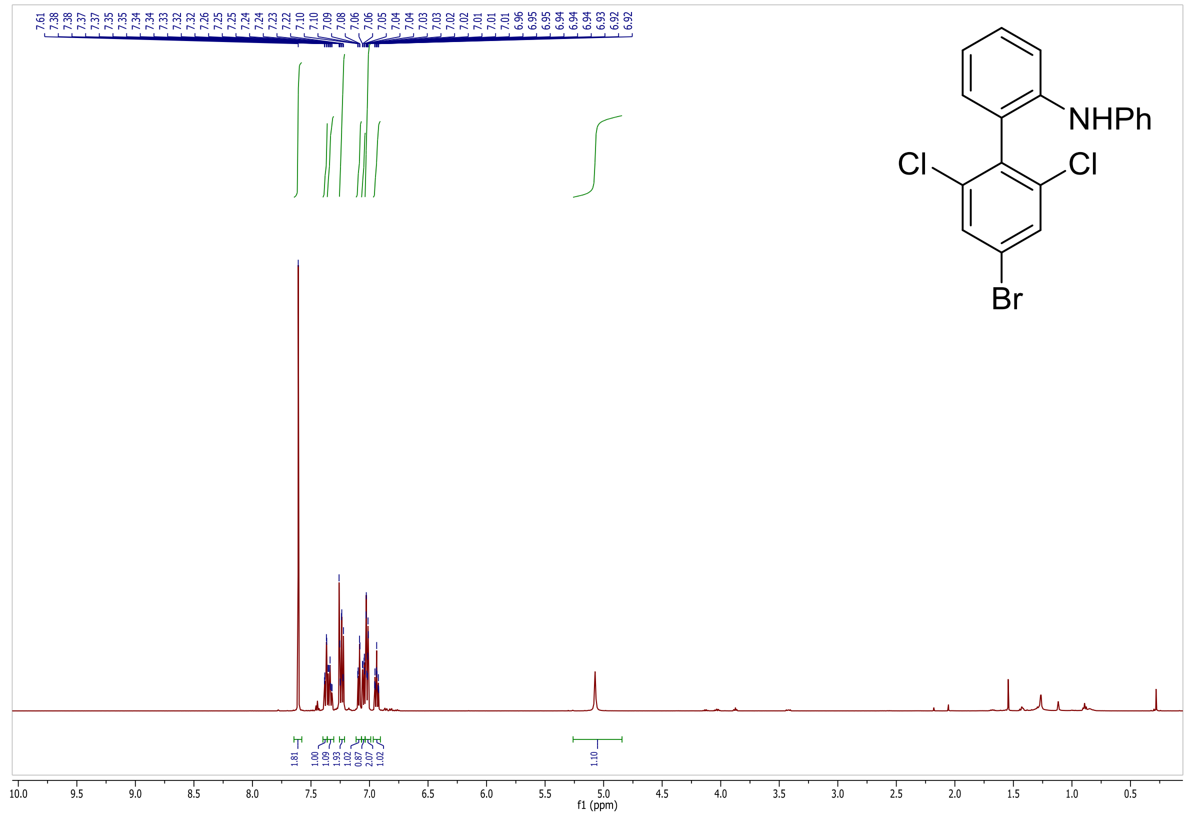

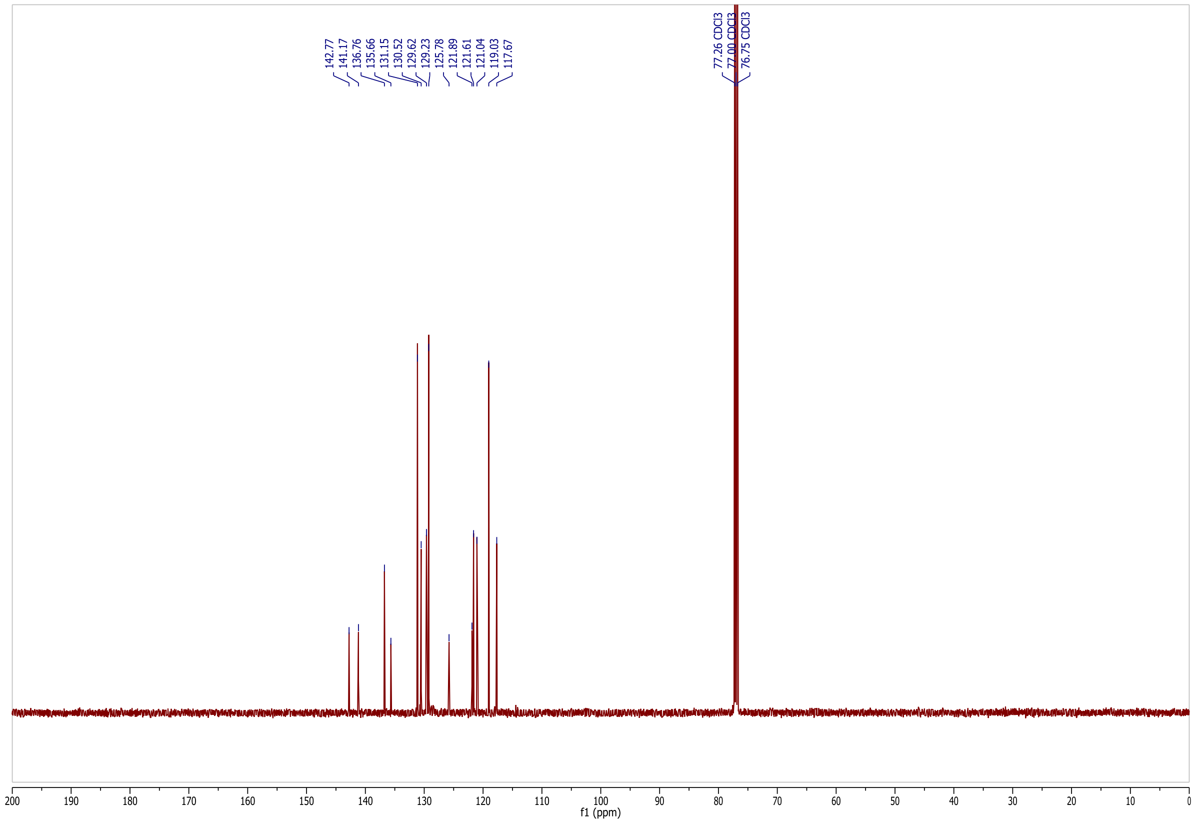

^1H and ^{13}C Spectra ($CDCl_3$, 400 and 101 MHz) for compound 4v

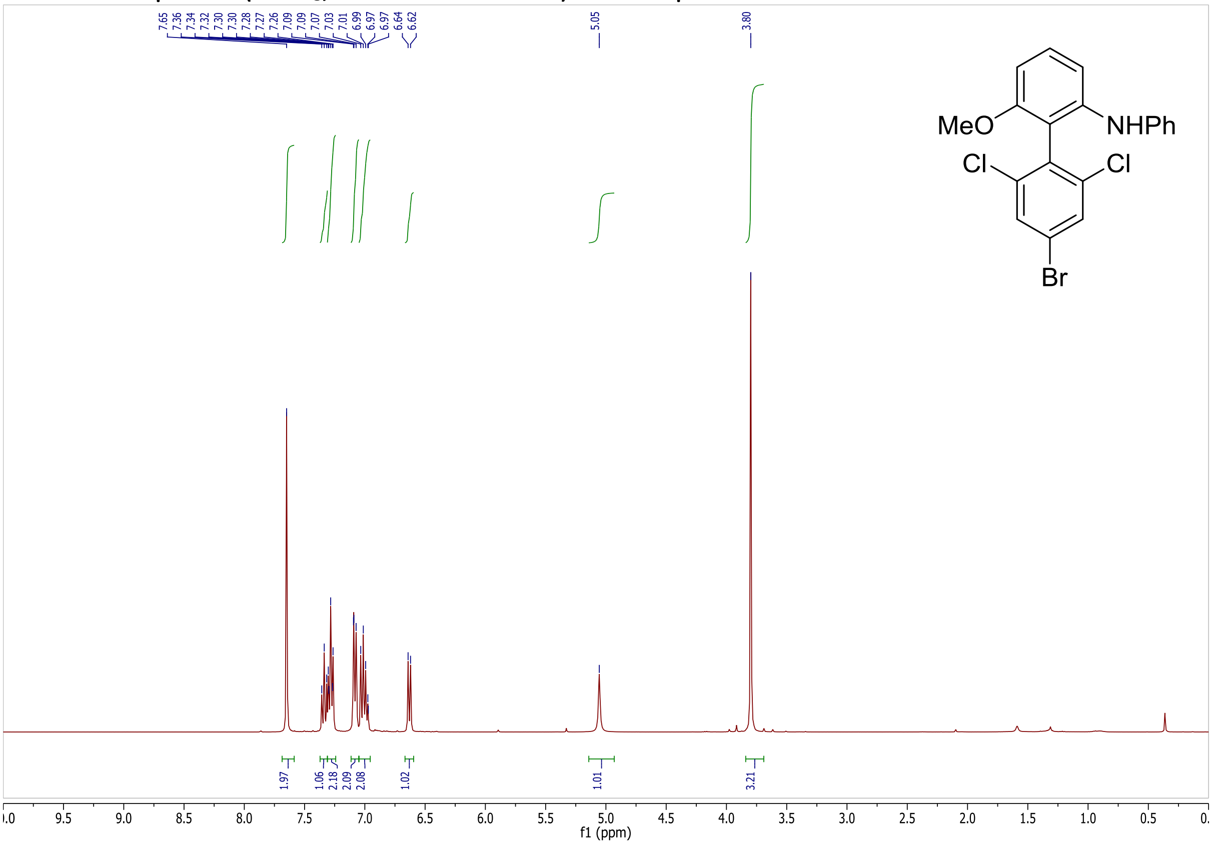

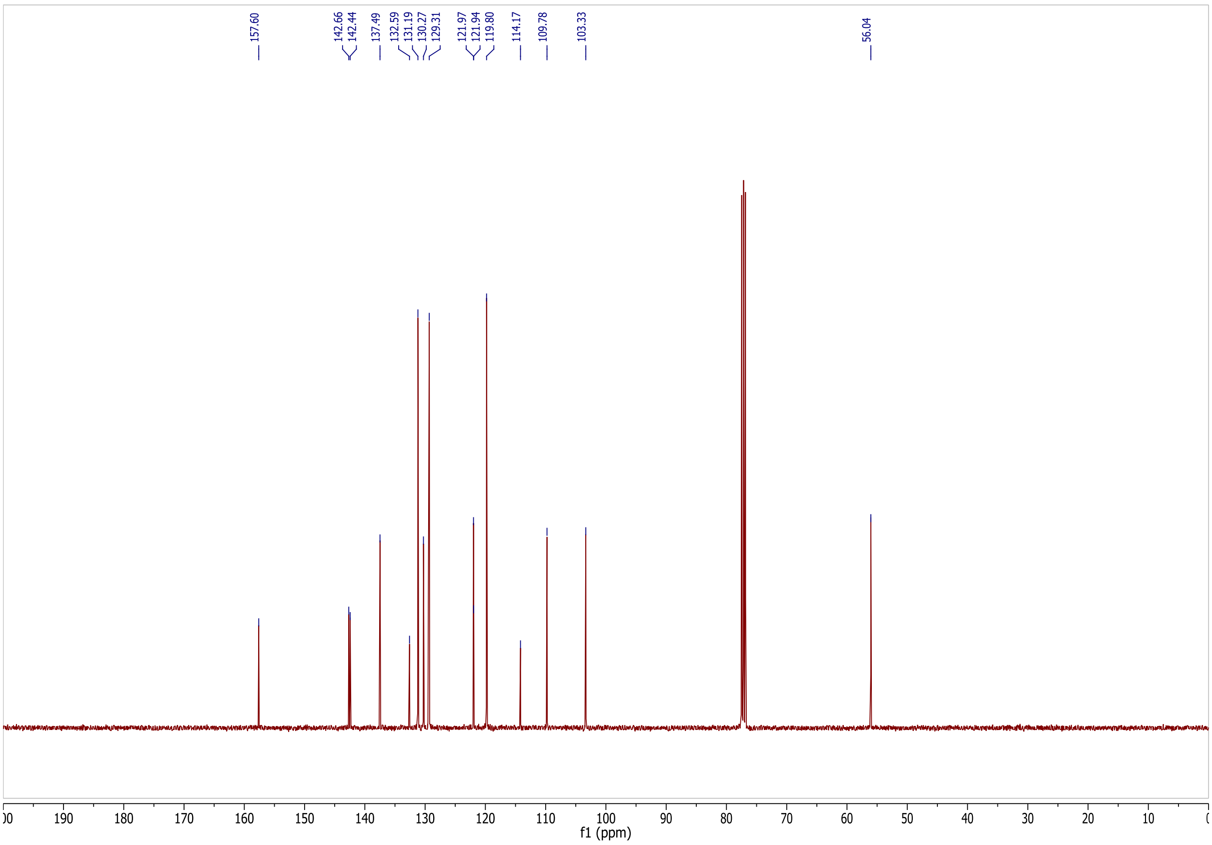

^{1}H and ^{13}C Spectra ($CDCl_3$, 400 and 101 MHz) for compounds 4w

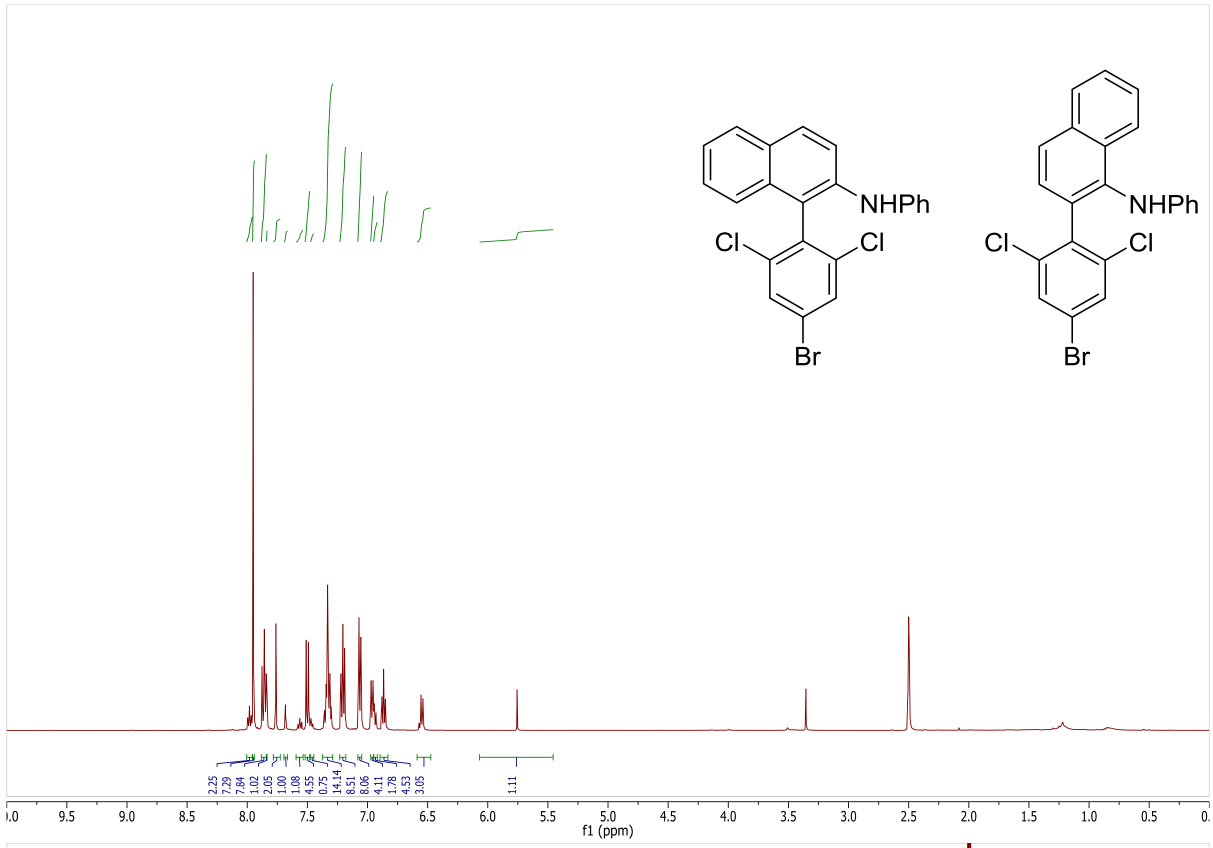

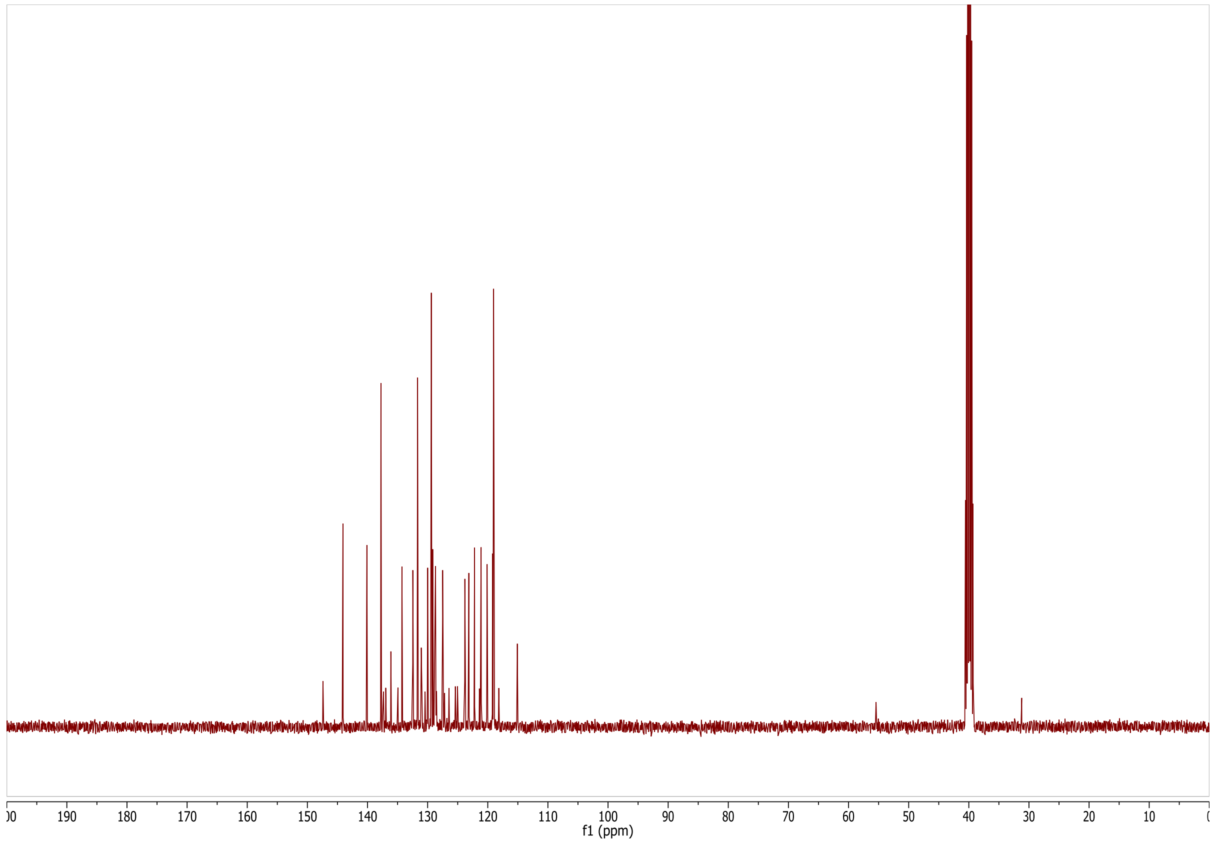

^1H and ^{13}C Spectra ($CDCl_3$, 400 and 101 MHz) for compound 4x

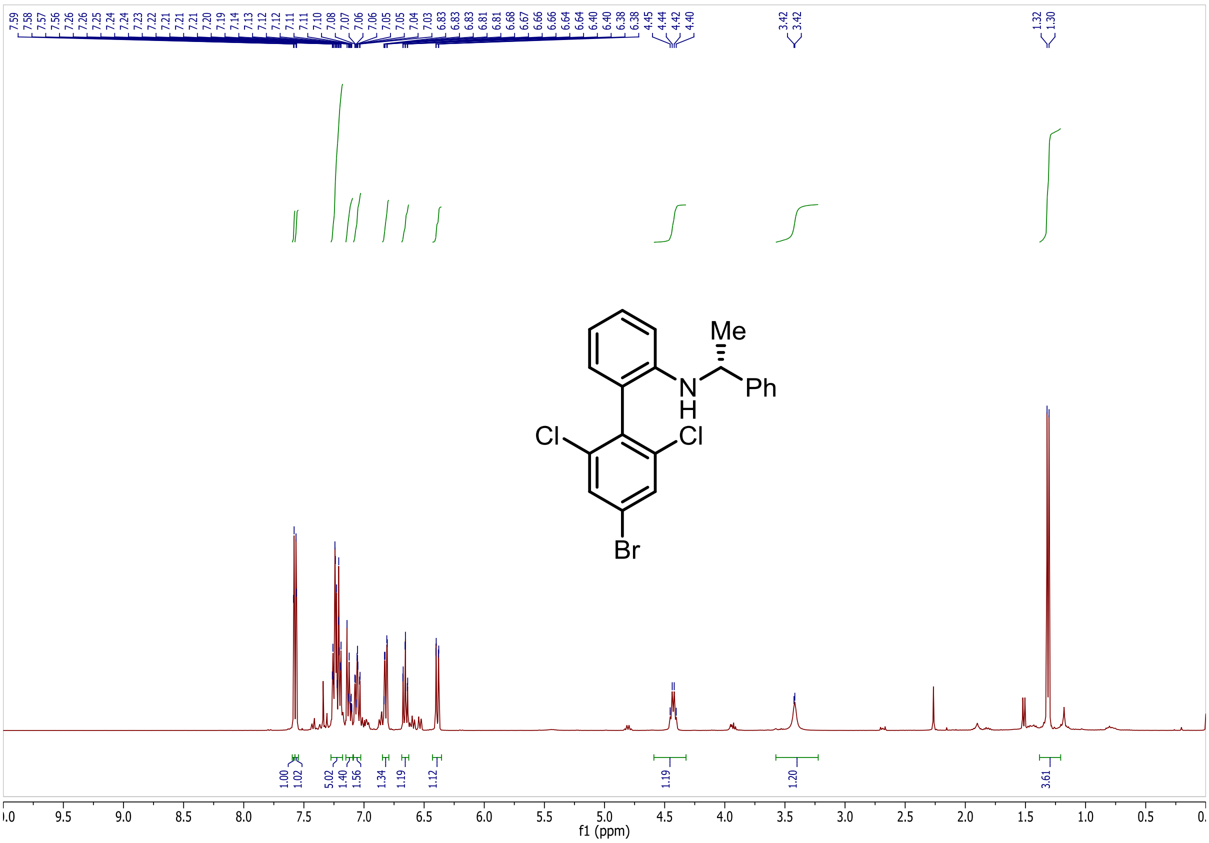

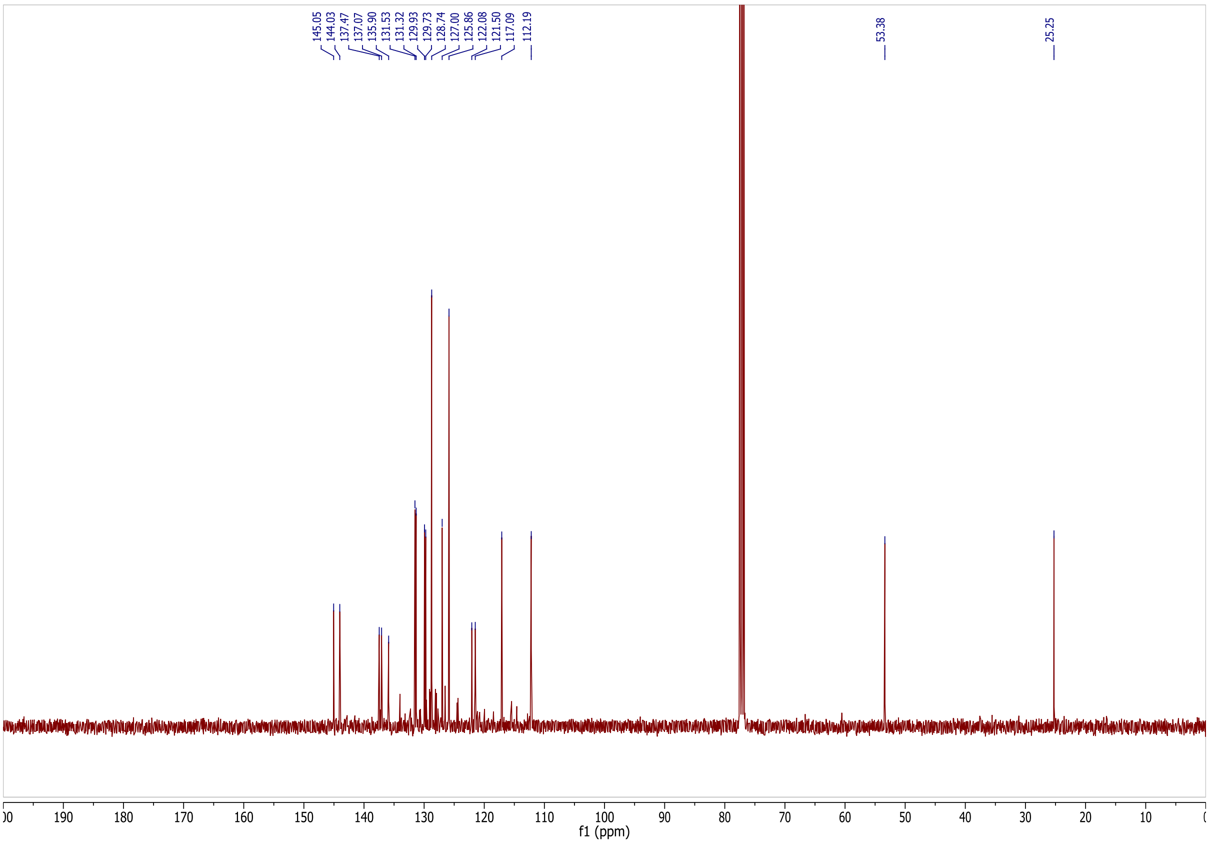

^{1}H and ^{13}C Spectra ($CDCl_3$, 500 and 126 MHz) for compound 4y

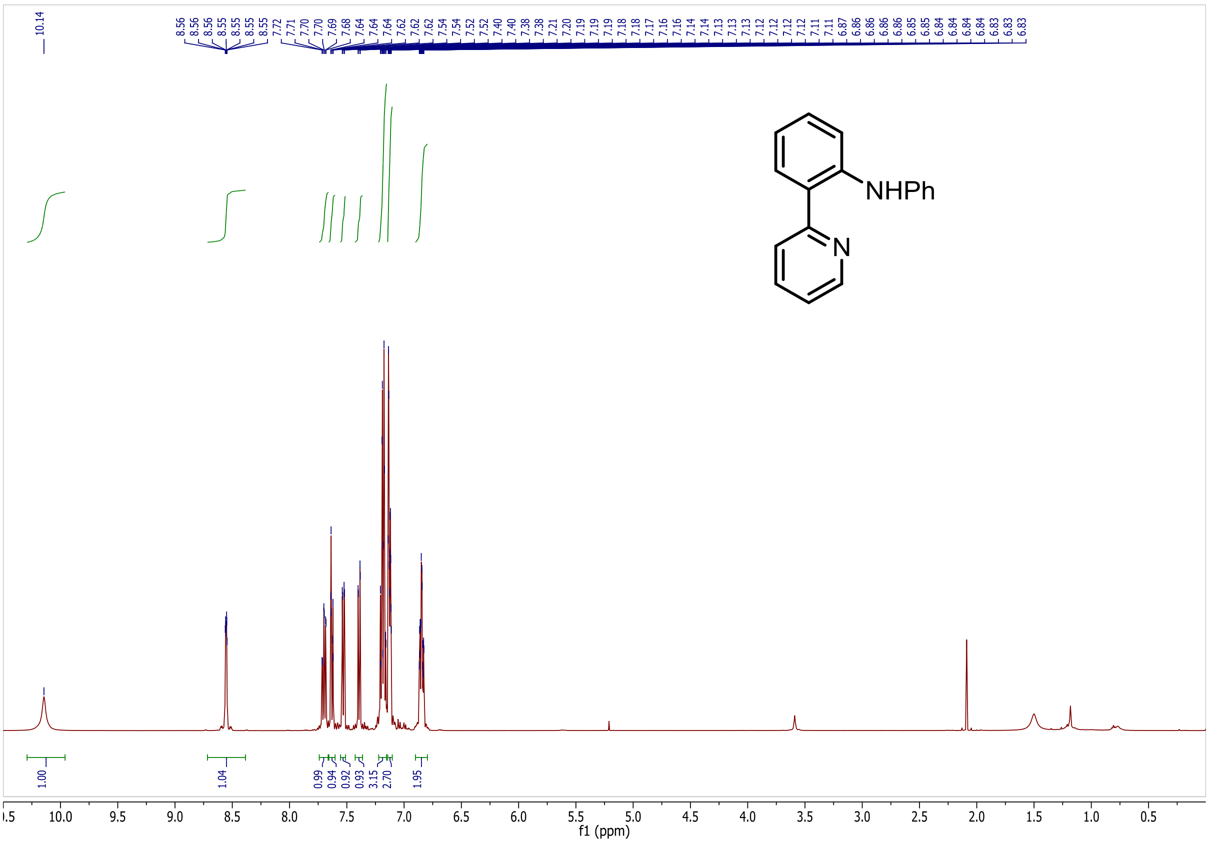

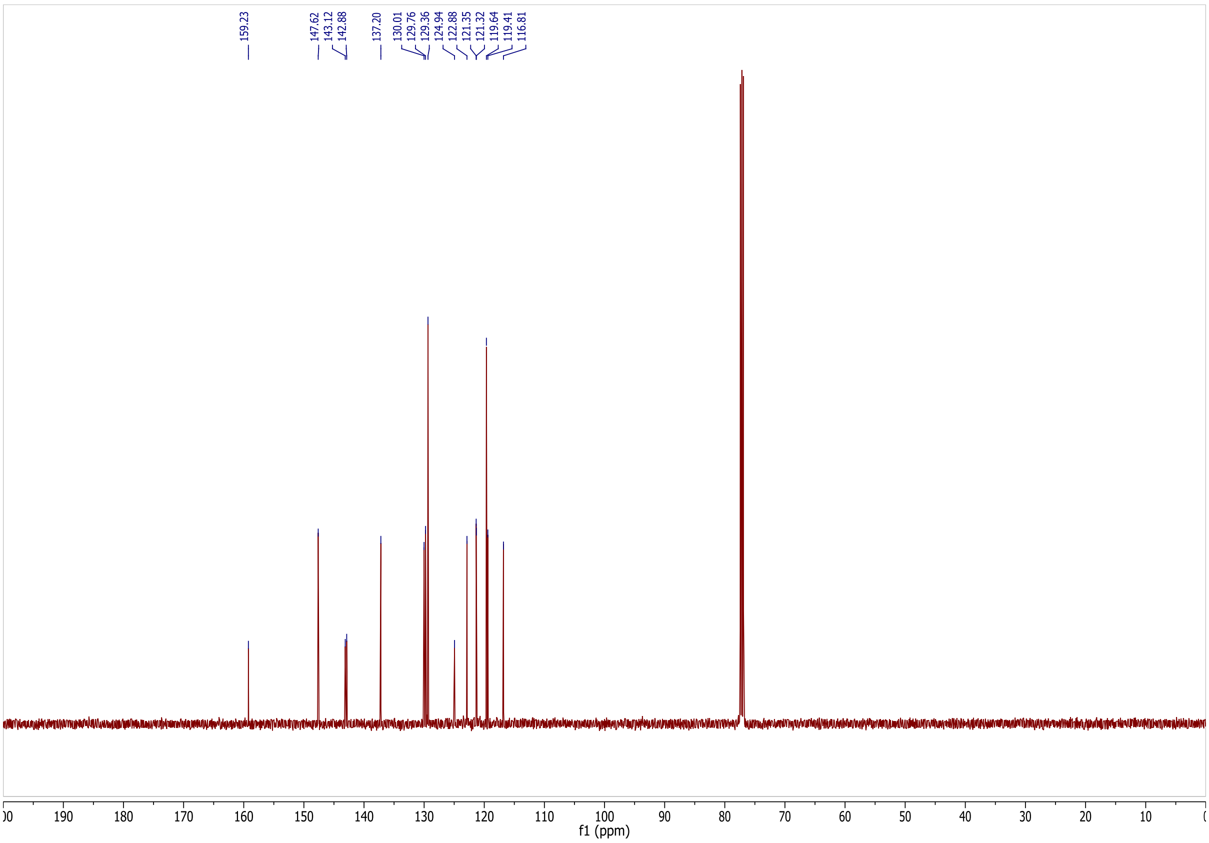

^{1}H and ^{13}C Spectra ($CDCl_3$, 400 and 101 MHz) for compound 4z

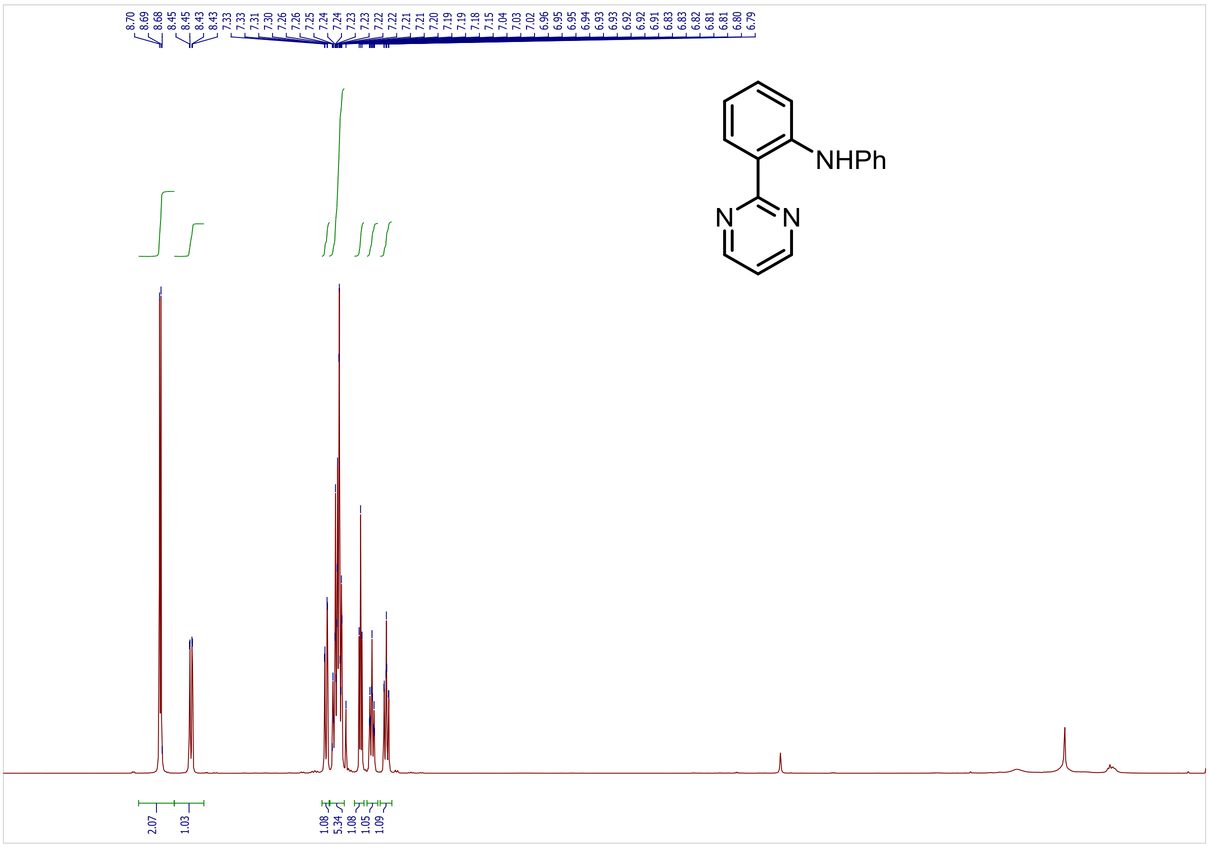

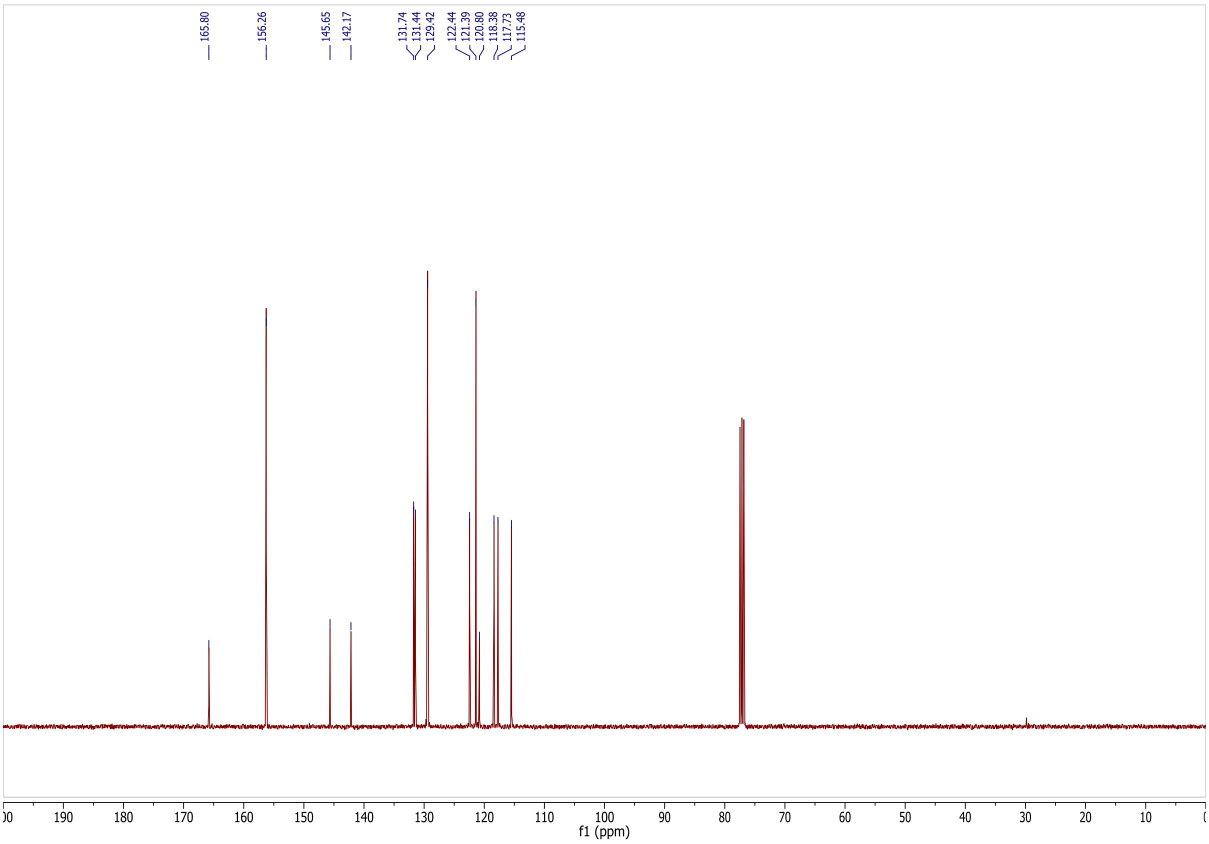

^1H and ^{13}C Spectra ($CDCl_3$, 400 and 101 MHz) for compound 4zz

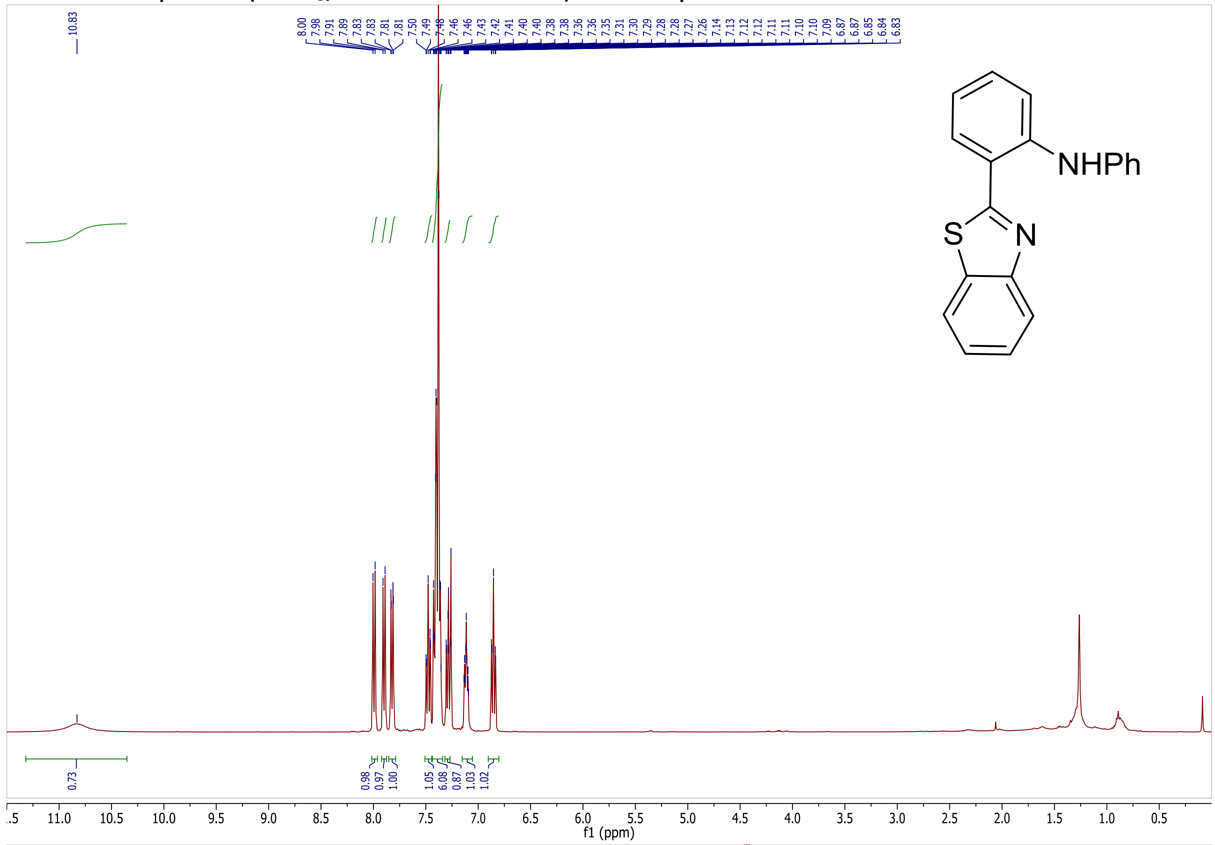

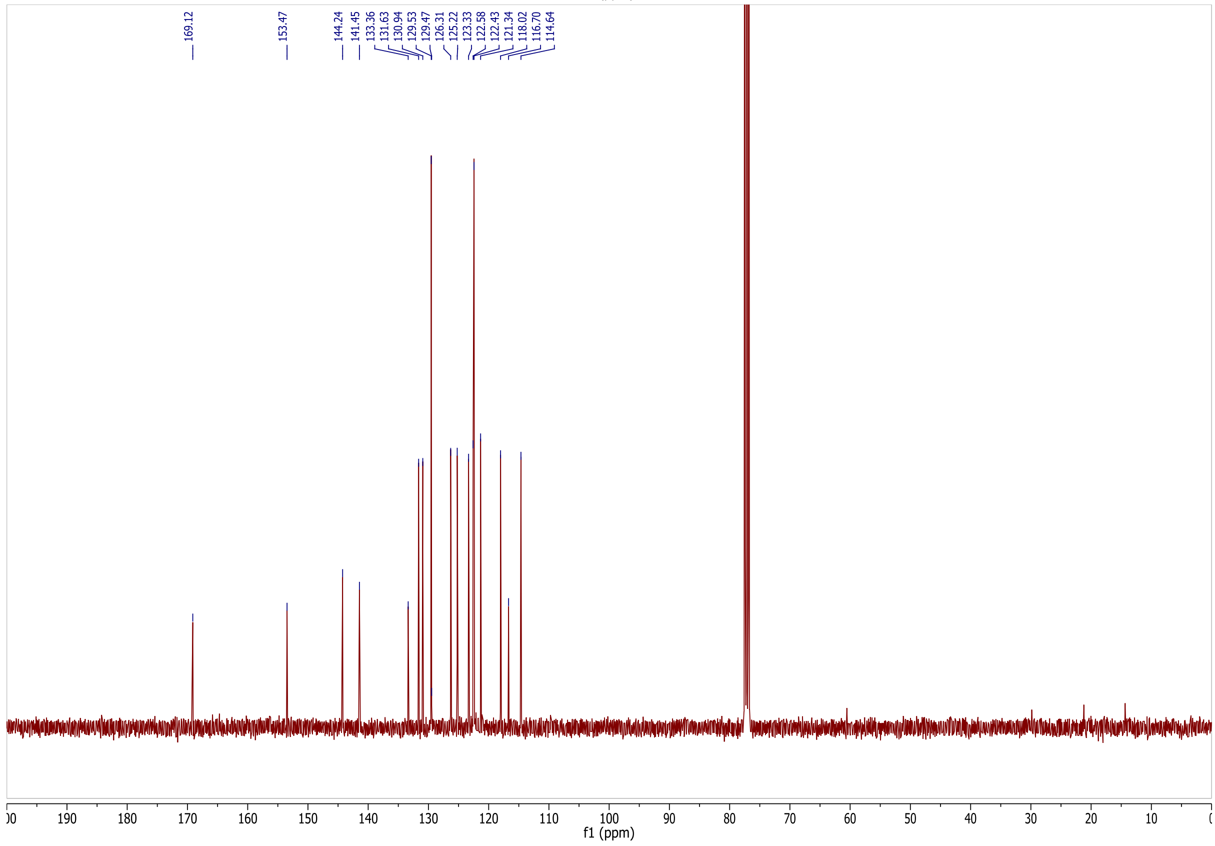

^{1}H and ^{13}C Spectra ($CDCl_3$, 400 and 101 MHz) for compound 7a

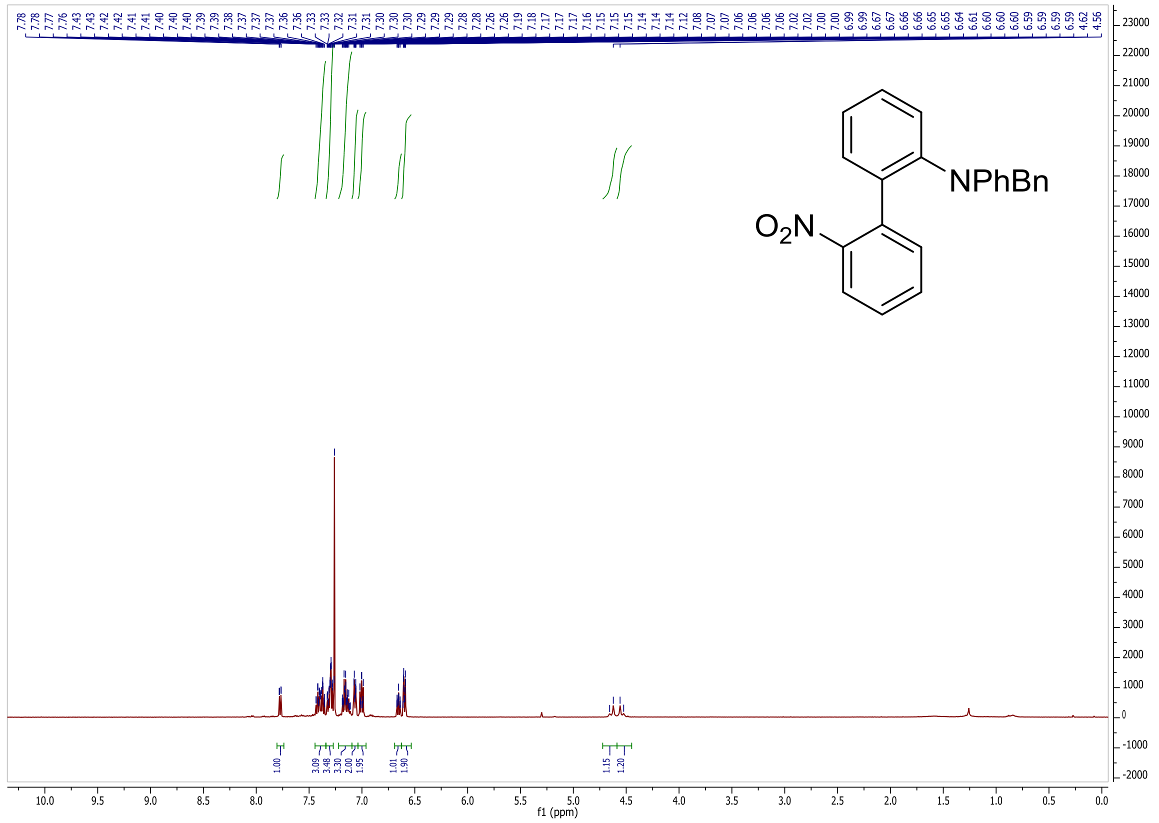

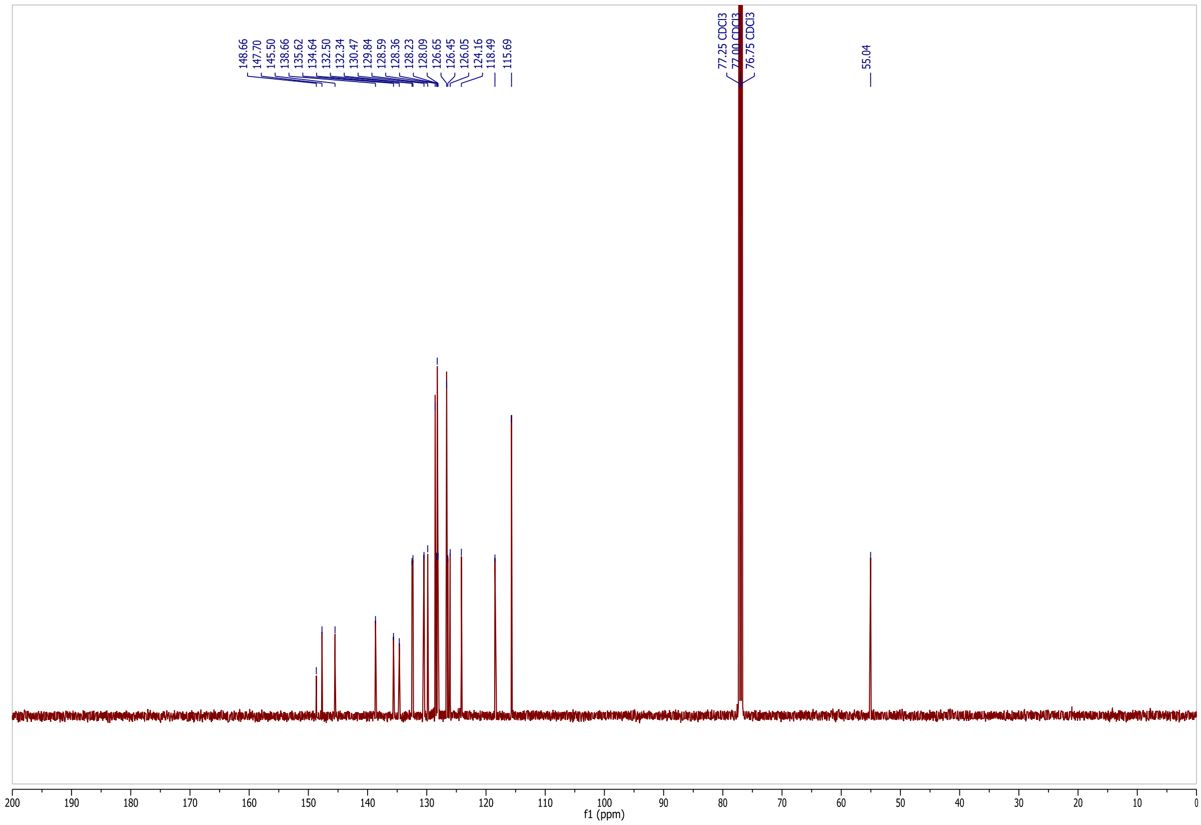

^{1}H and ^{13}C Spectra ($CDCl_3$, 400 and 101 MHz) for compound 7b

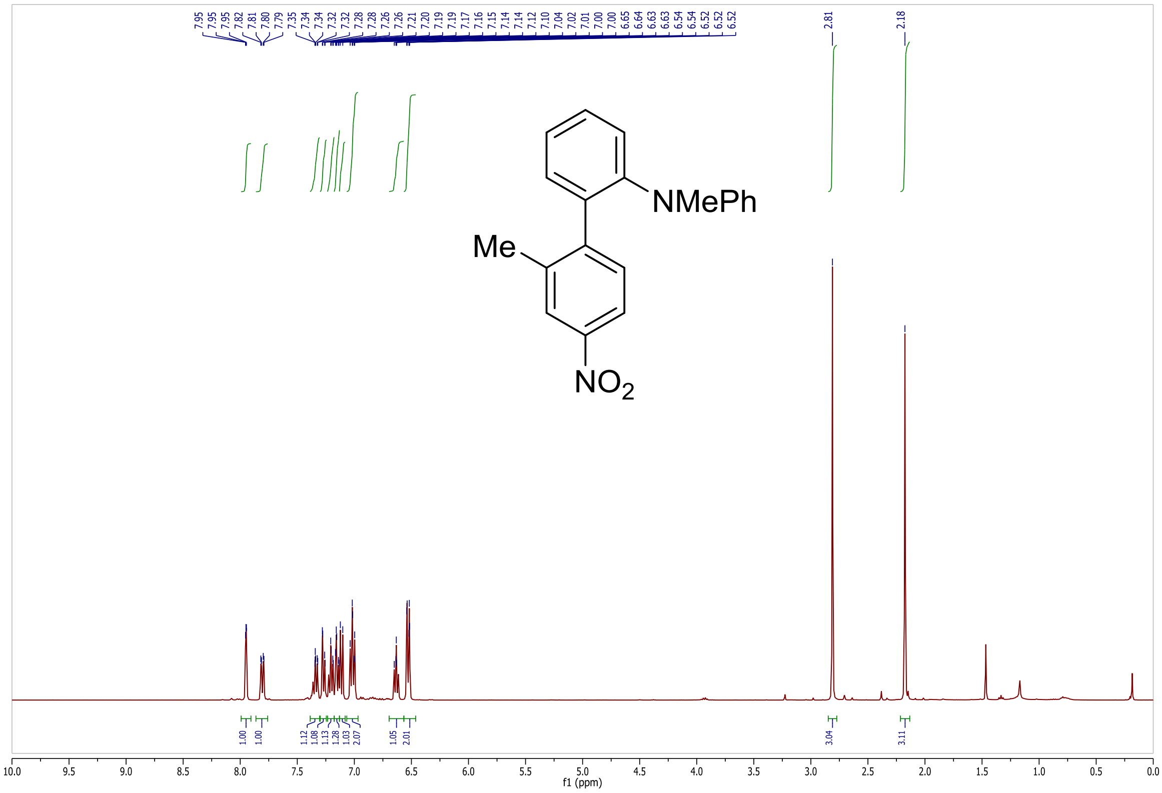

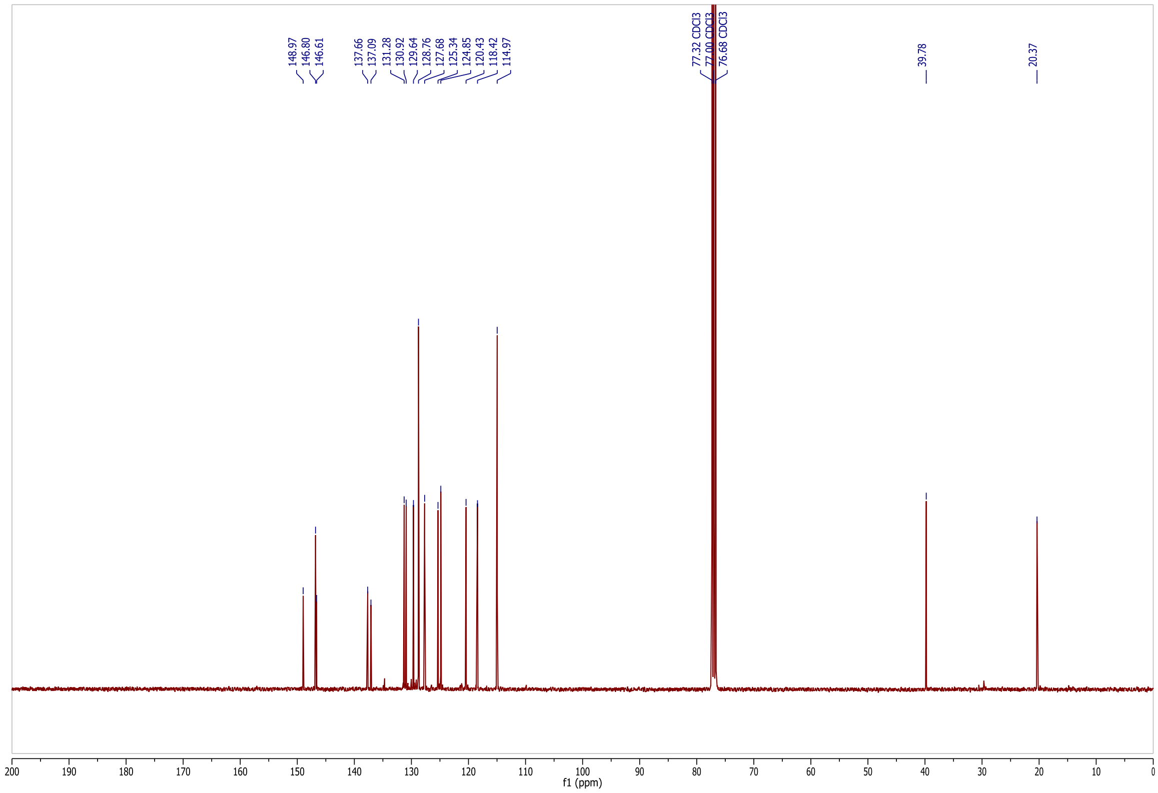

^1H and ^{13}C Spectra ($CDCl_3$, 400 and 101 MHz) for compound 7c

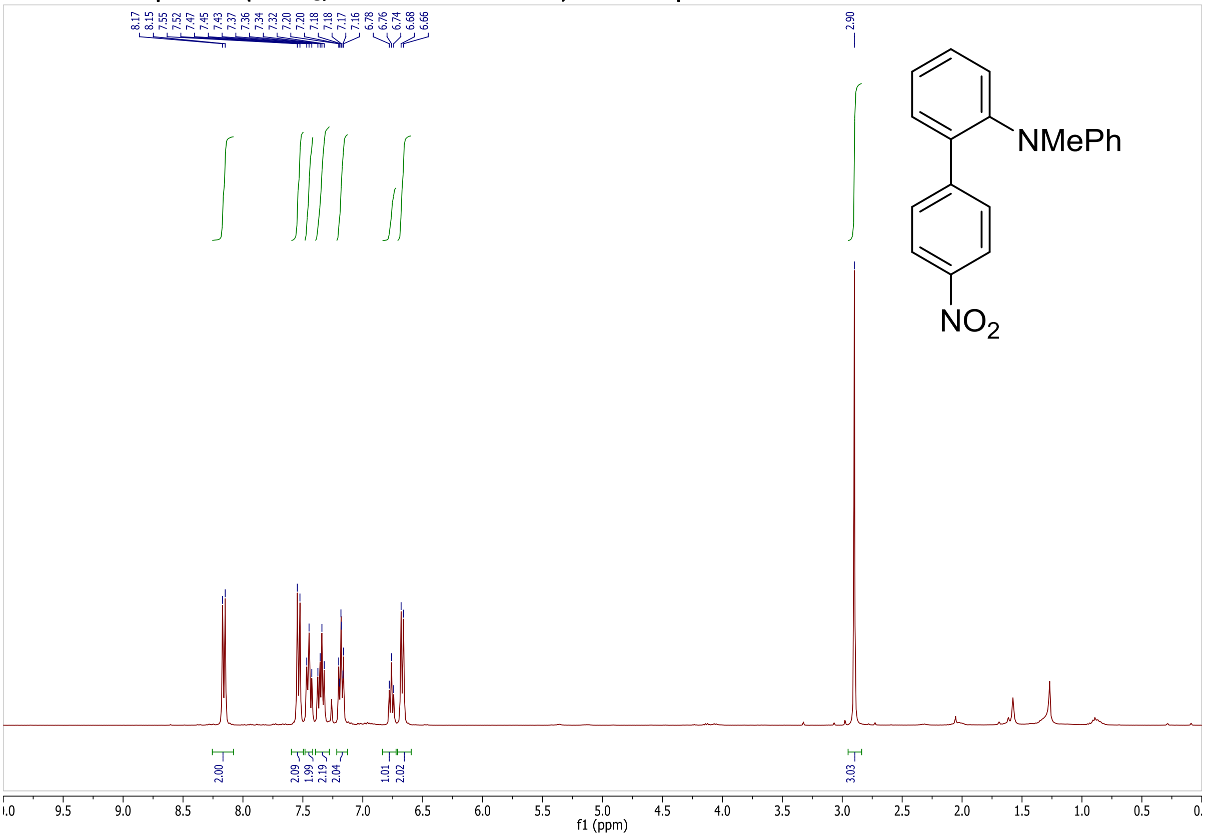

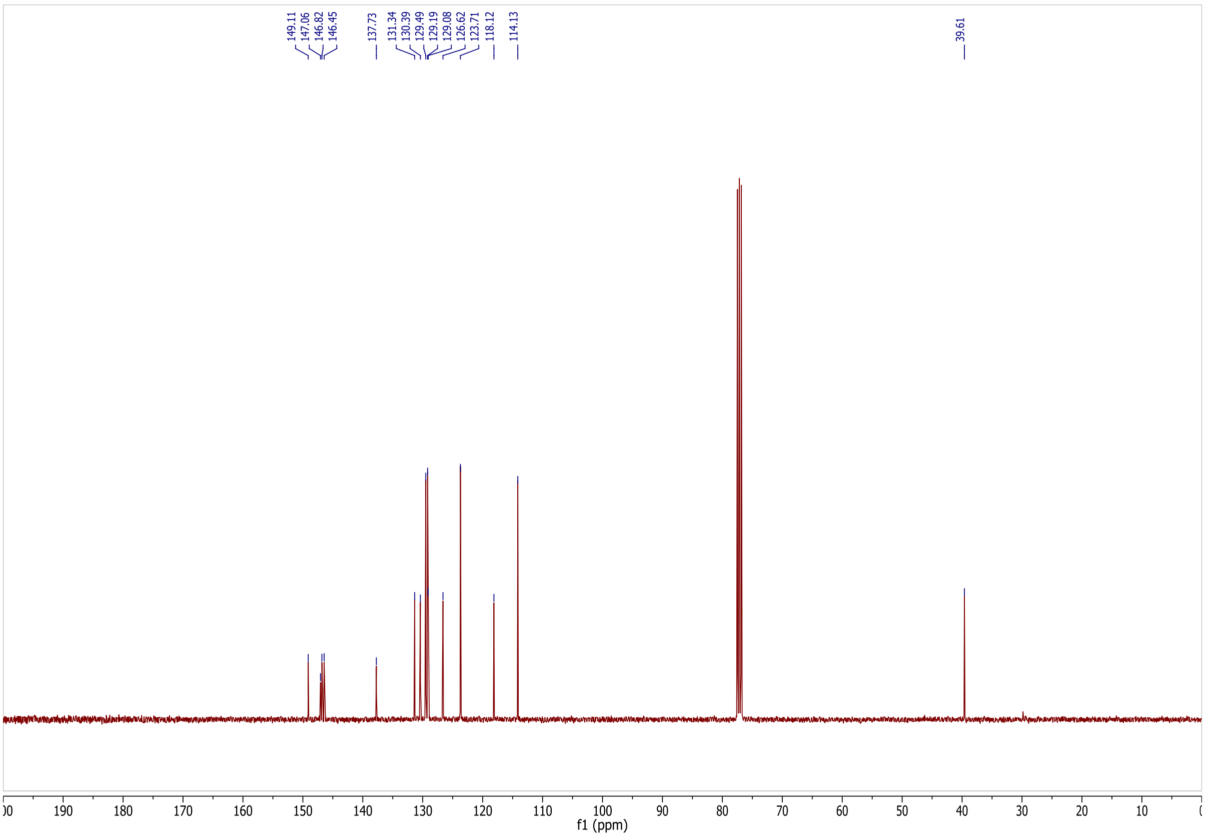

^1H and ^{13}C Spectra ($CDCl_3$, 400 and 101 MHz) for compound 7d

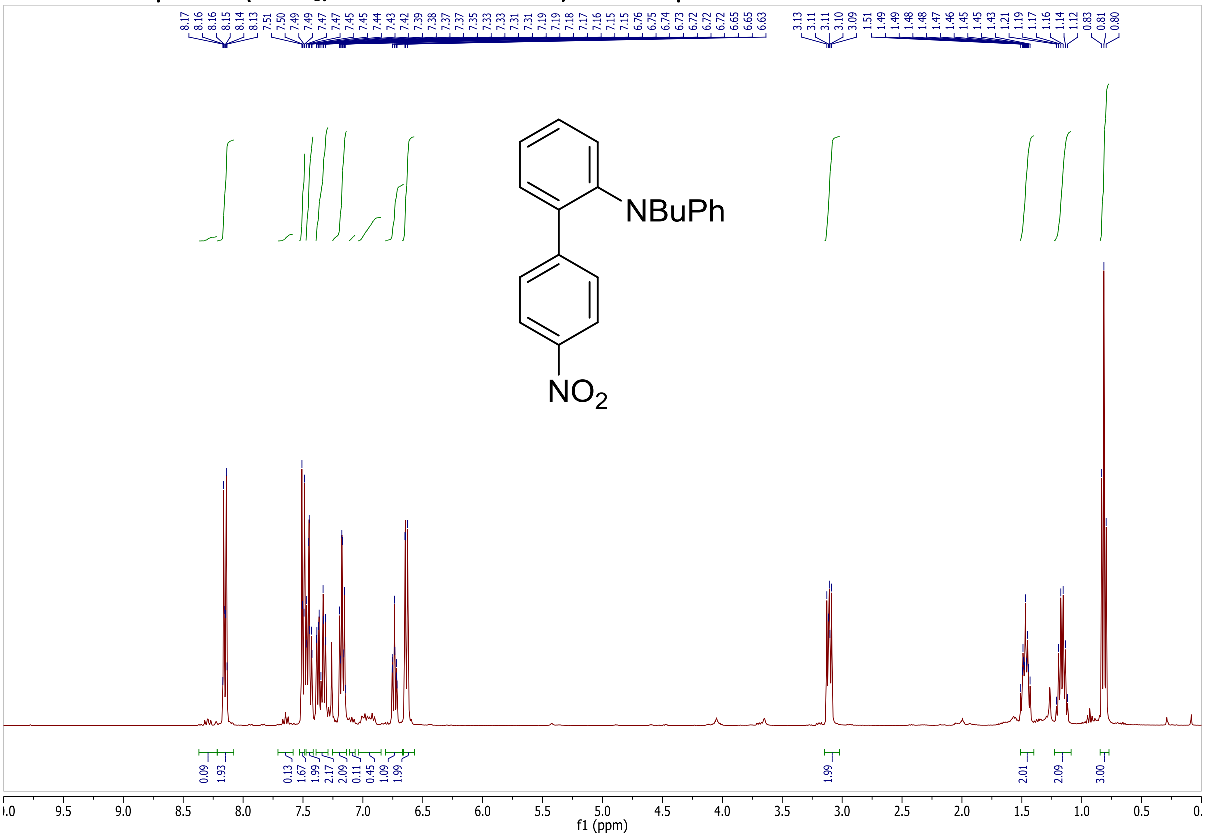

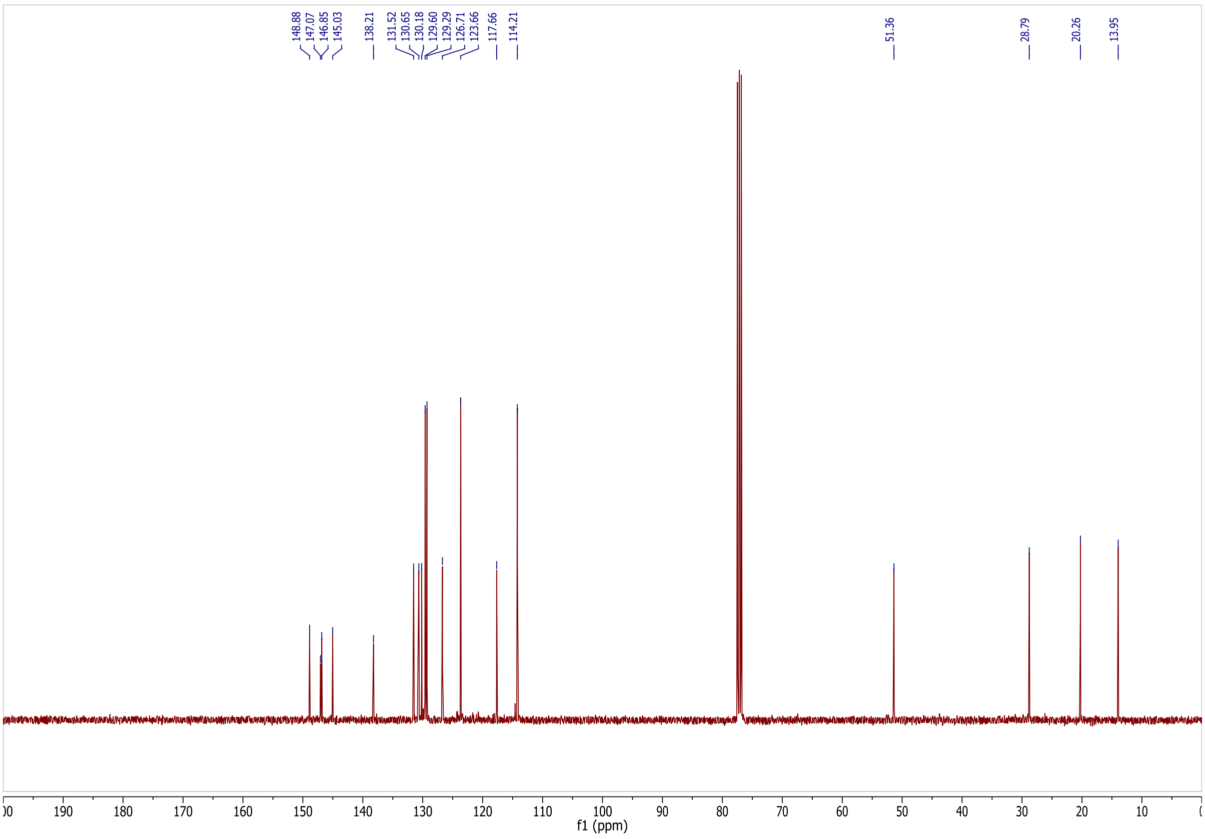

^1H and ^{13}C Spectra ($CDCl_3$, 500 and 126 MHz) for compound 7e

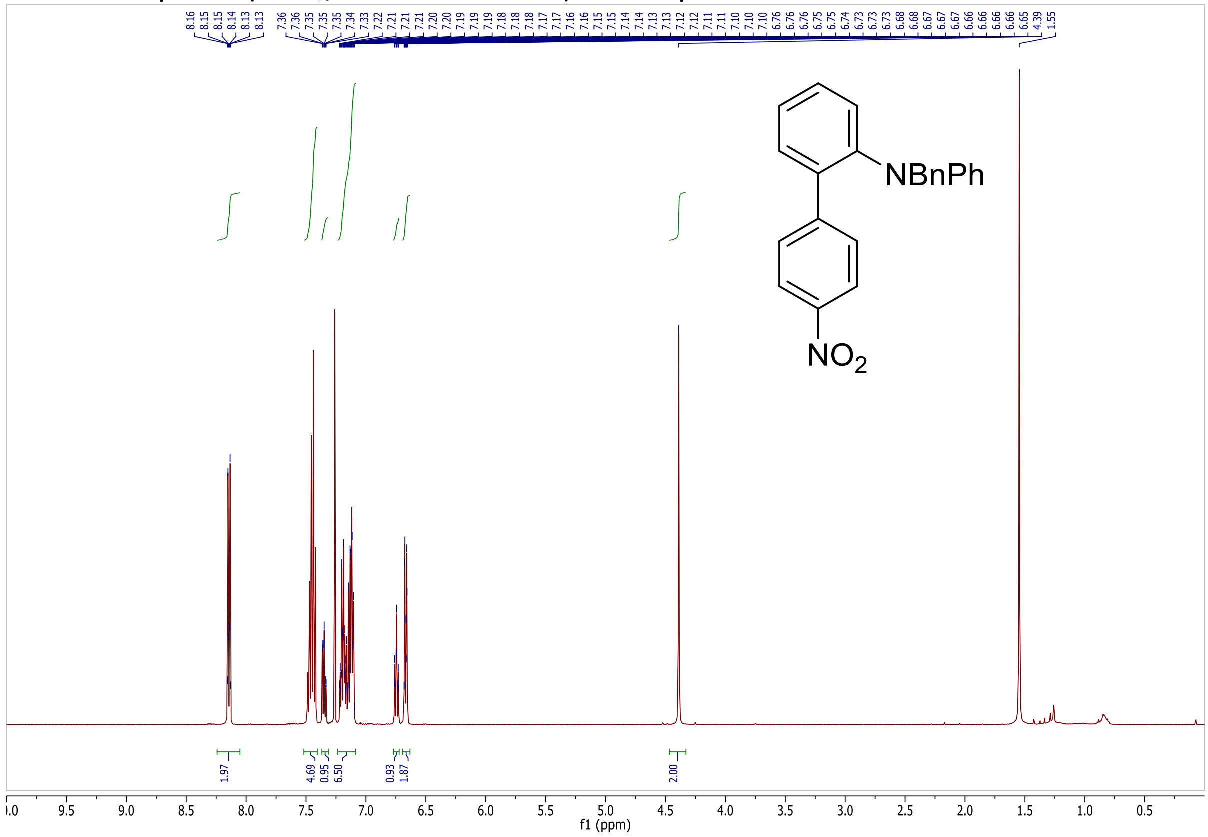

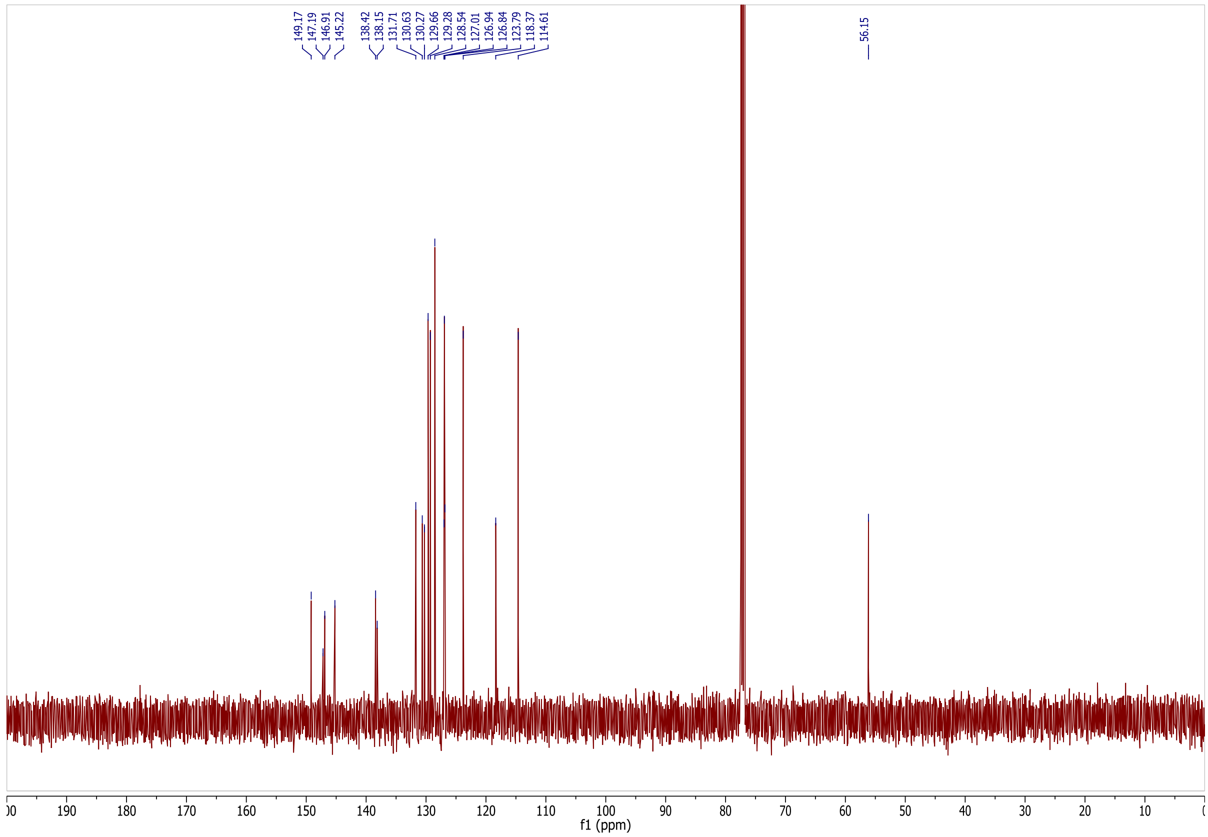

^{1}H and ^{13}C Spectra ($CDCl_3$, 400 and 101 MHz) for compound 7f

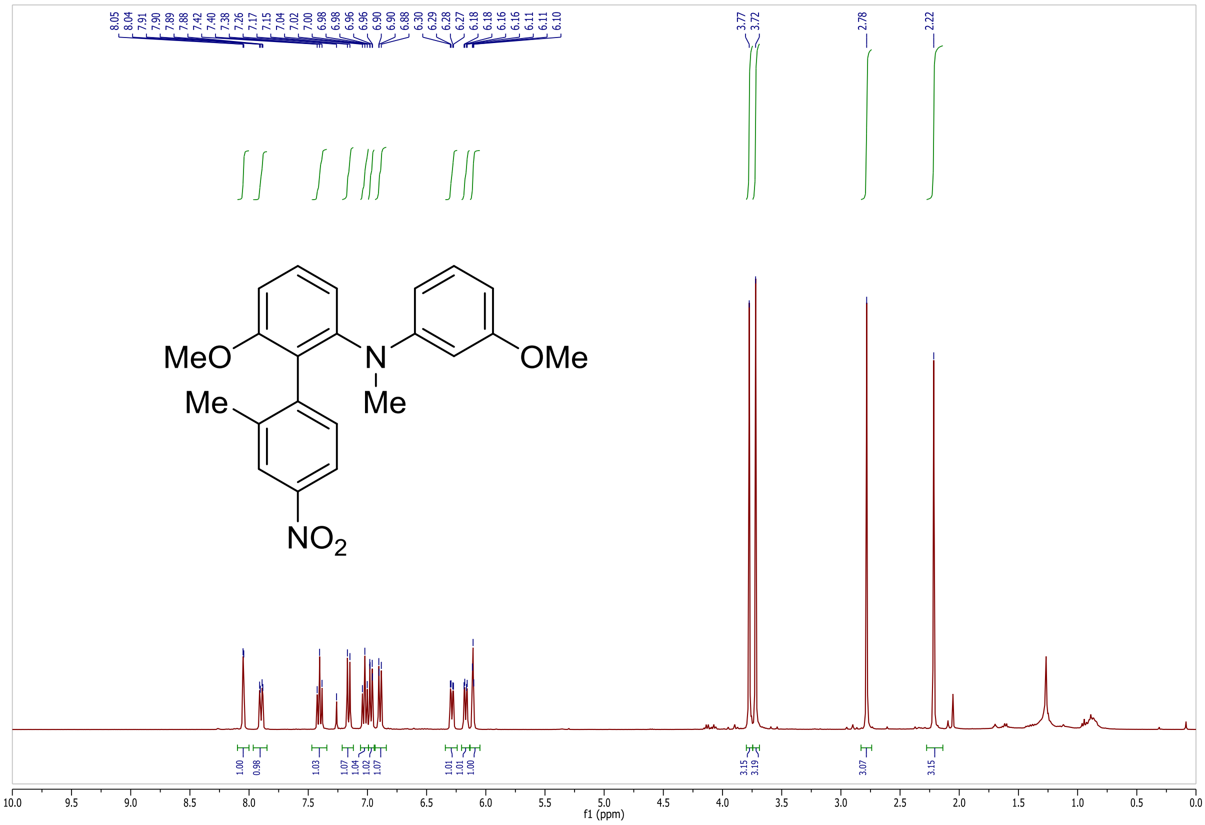

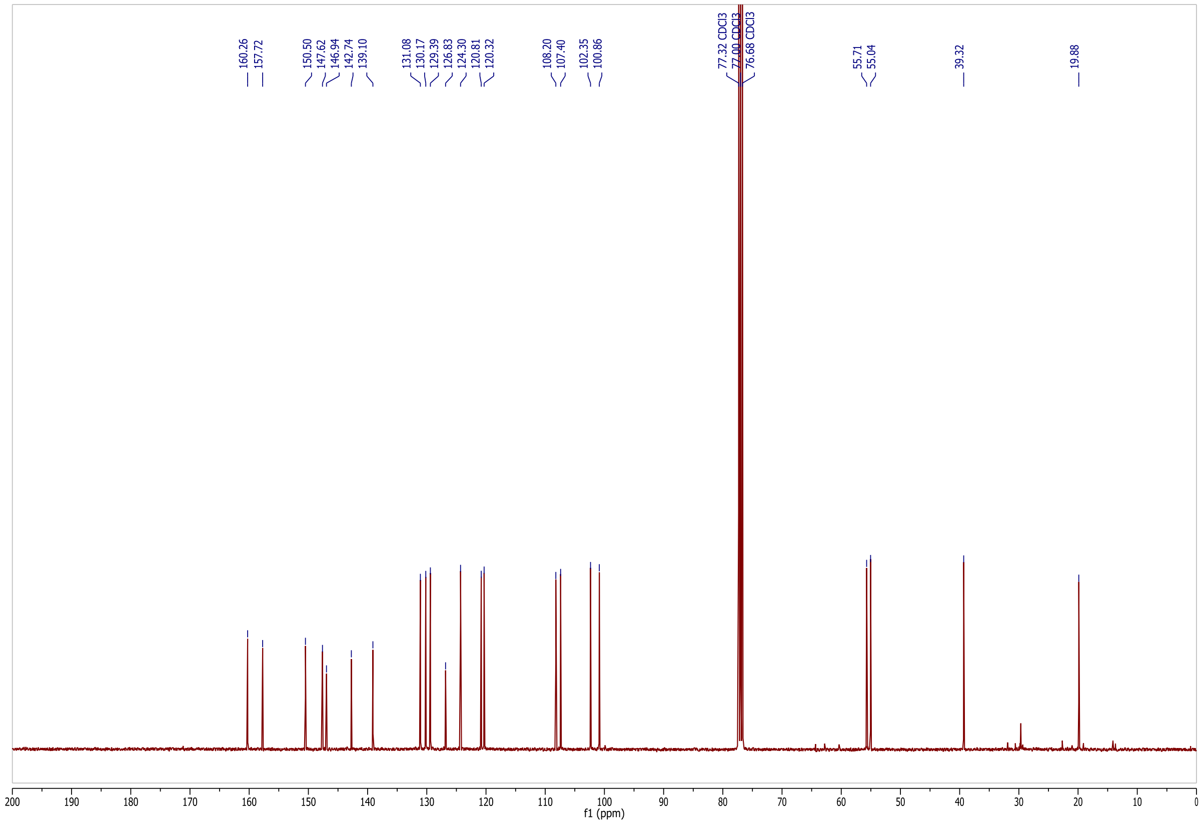

[1]H and [13]C Spectra (DMSO-*d6*, 400 and 101 MHz) for compound 8a

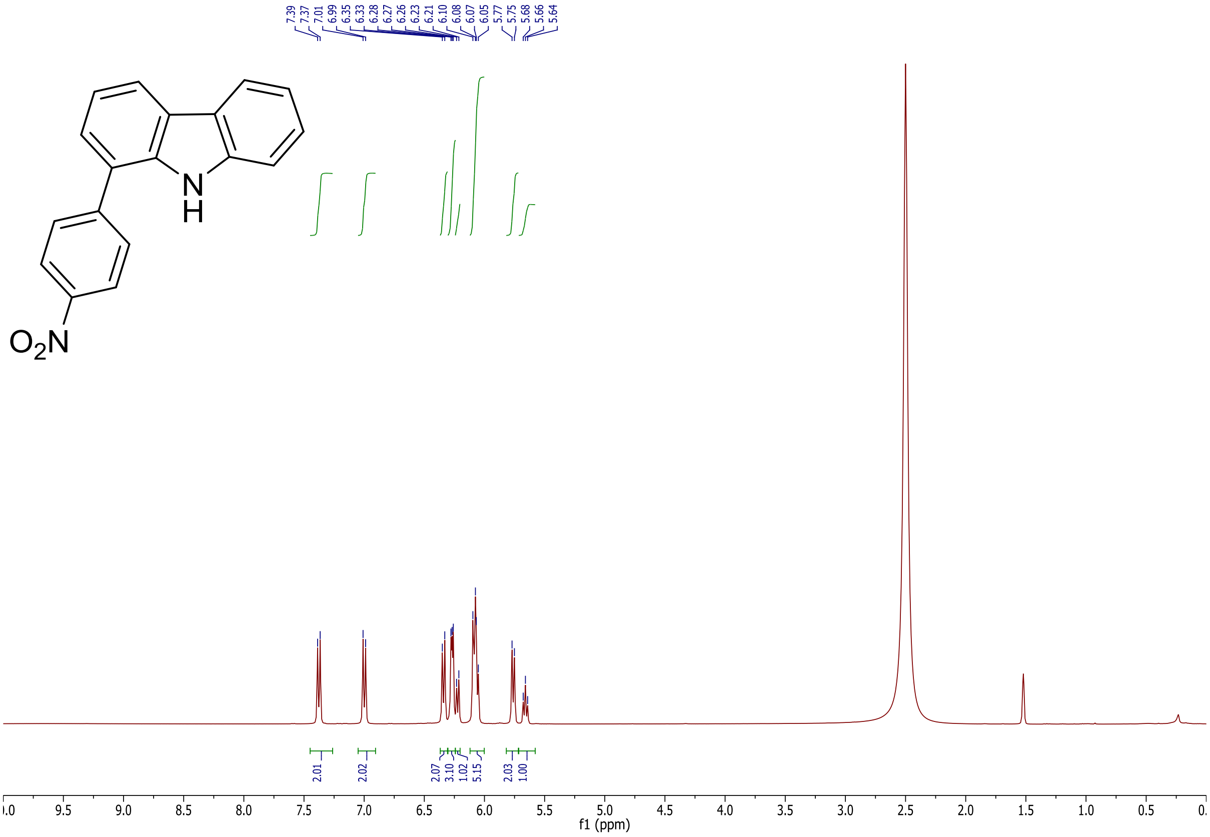

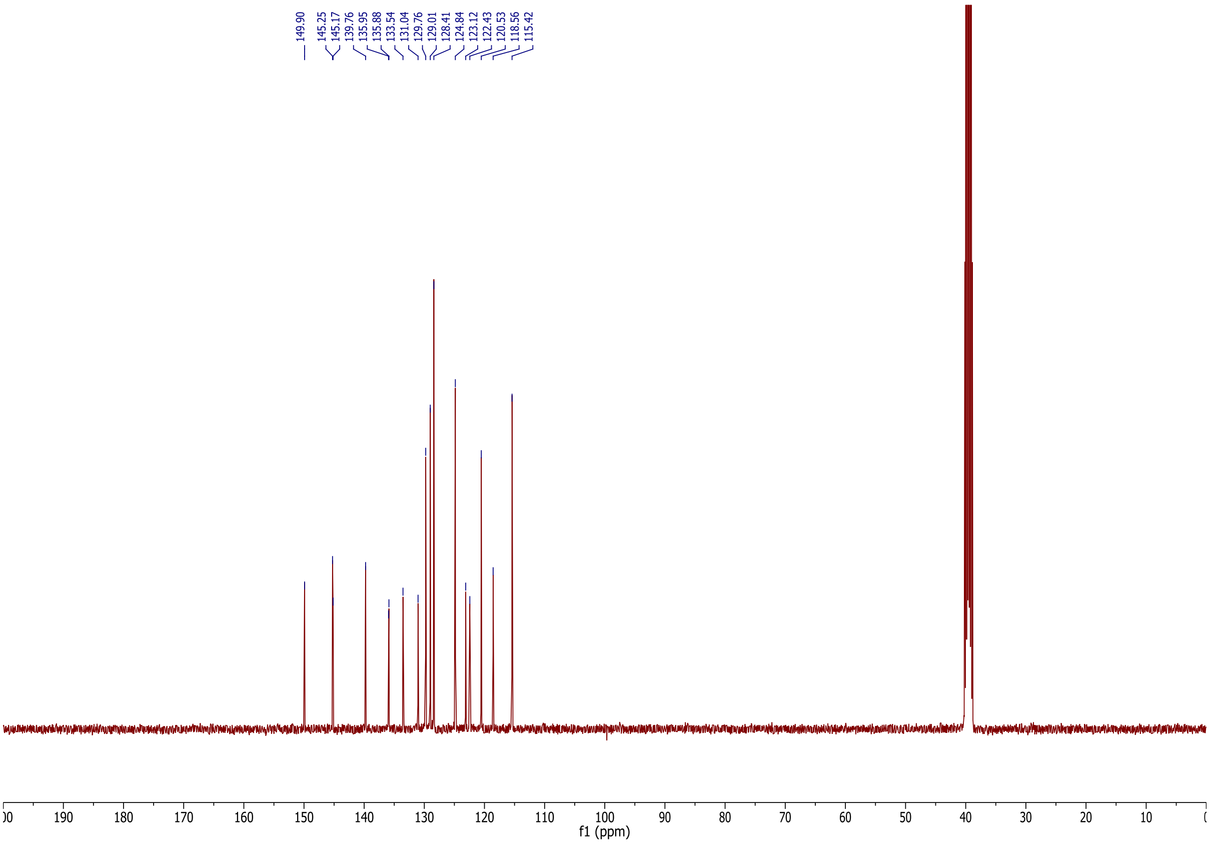

^{1}H and ^{13}C Spectra (CD_2Cl_2, 400 and 101 MHz) for compound 8b

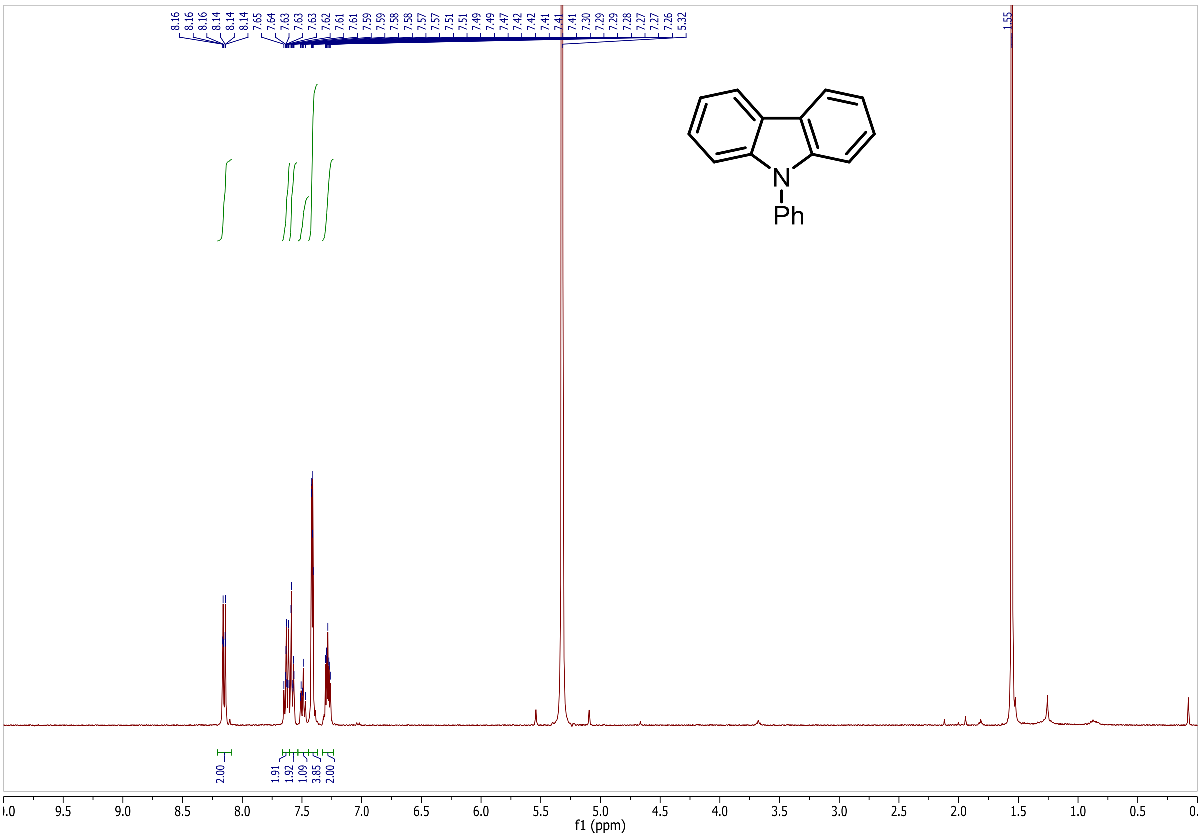

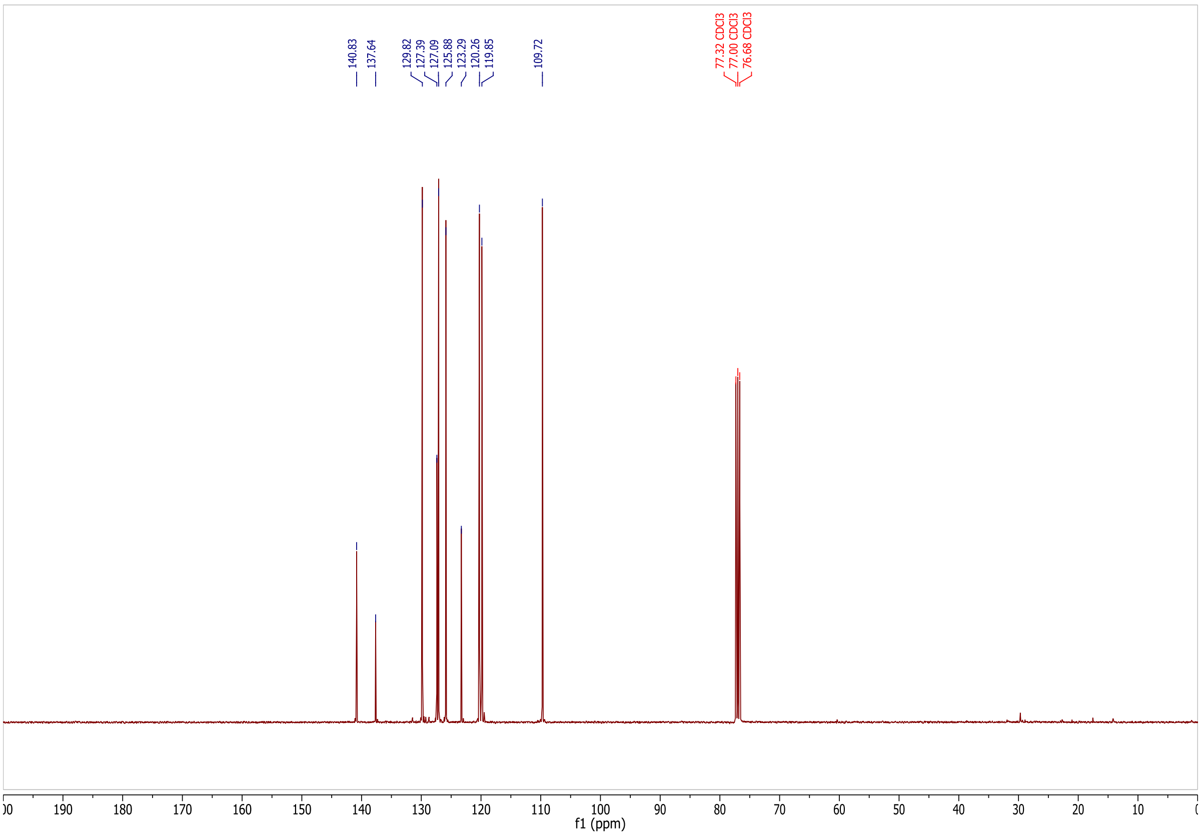

^{1}H and ^{13}C Spectra ($CDCl_3$, 400 and 101 MHz) for compound 12

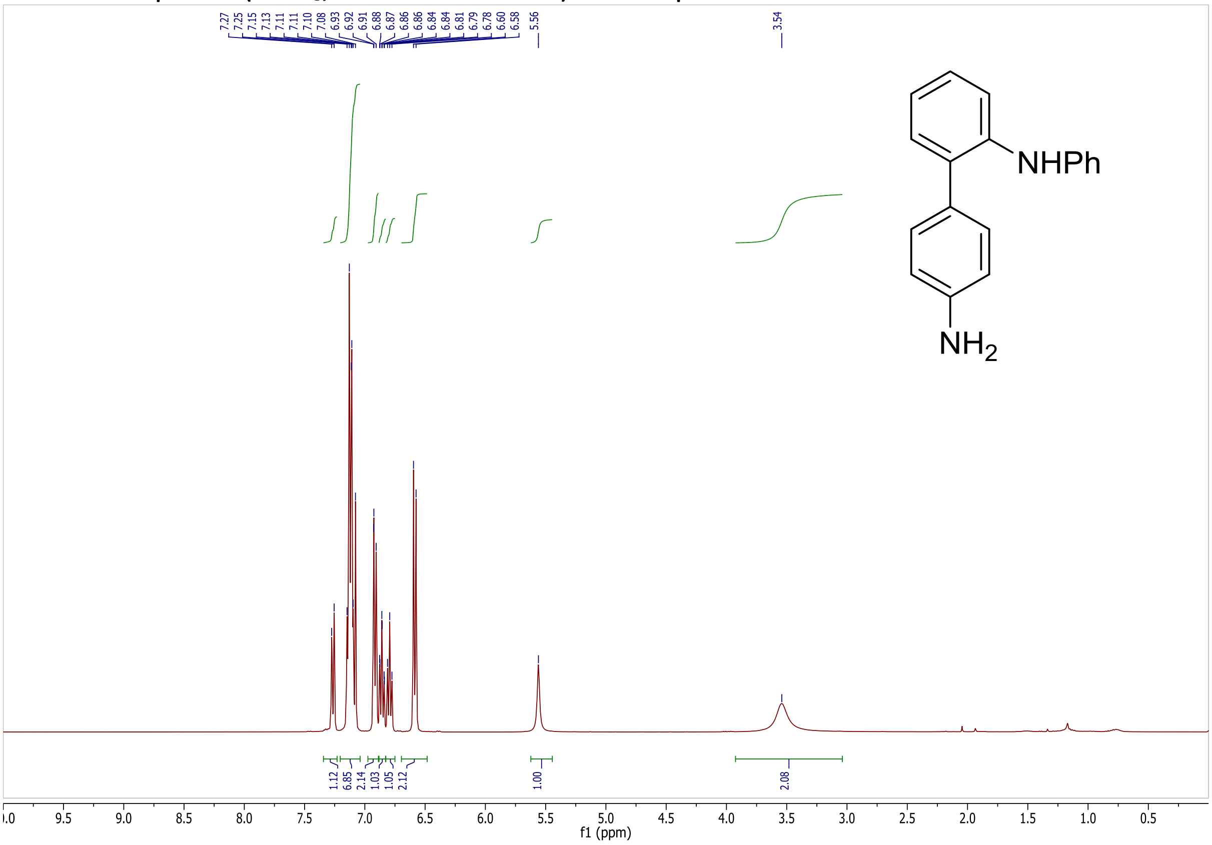

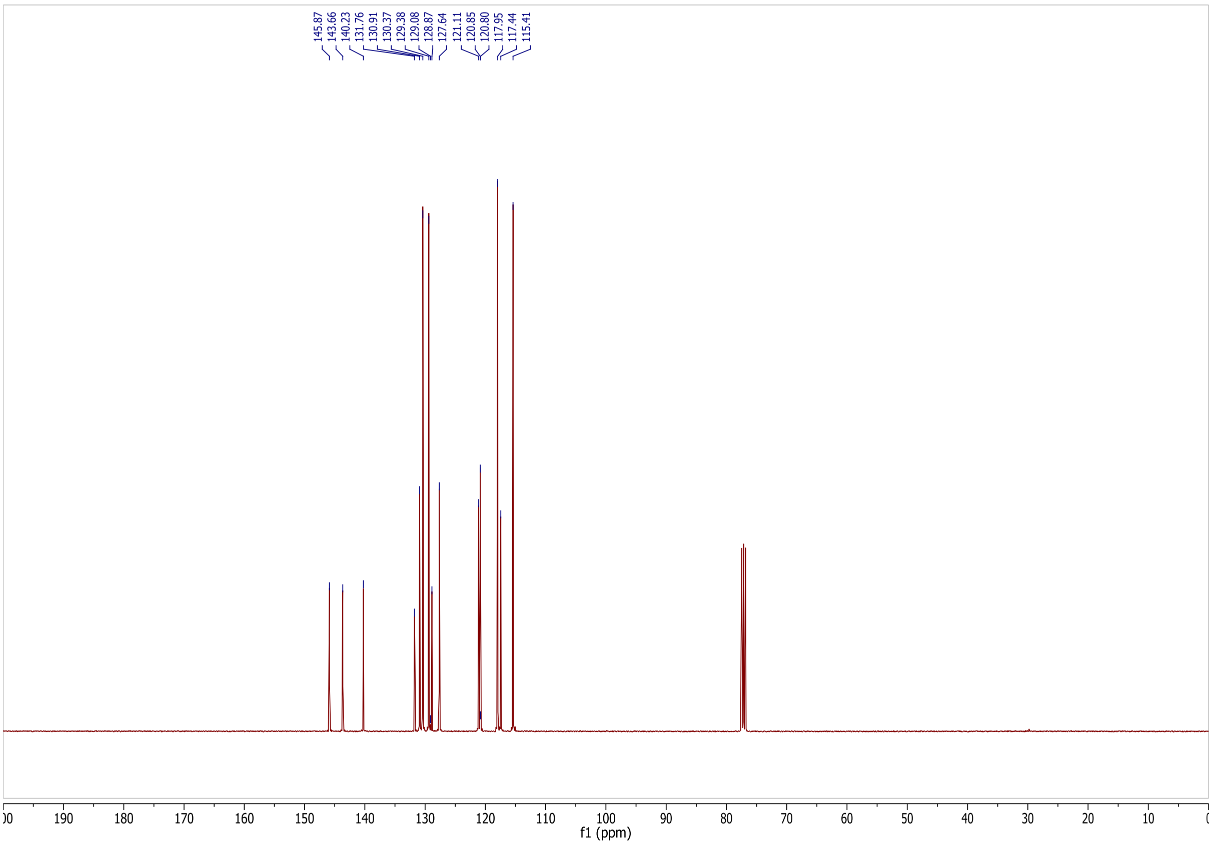

^{1}H and ^{13}C Spectra (DMSO-*d6*, 400 and 101 MHz) for compound 9

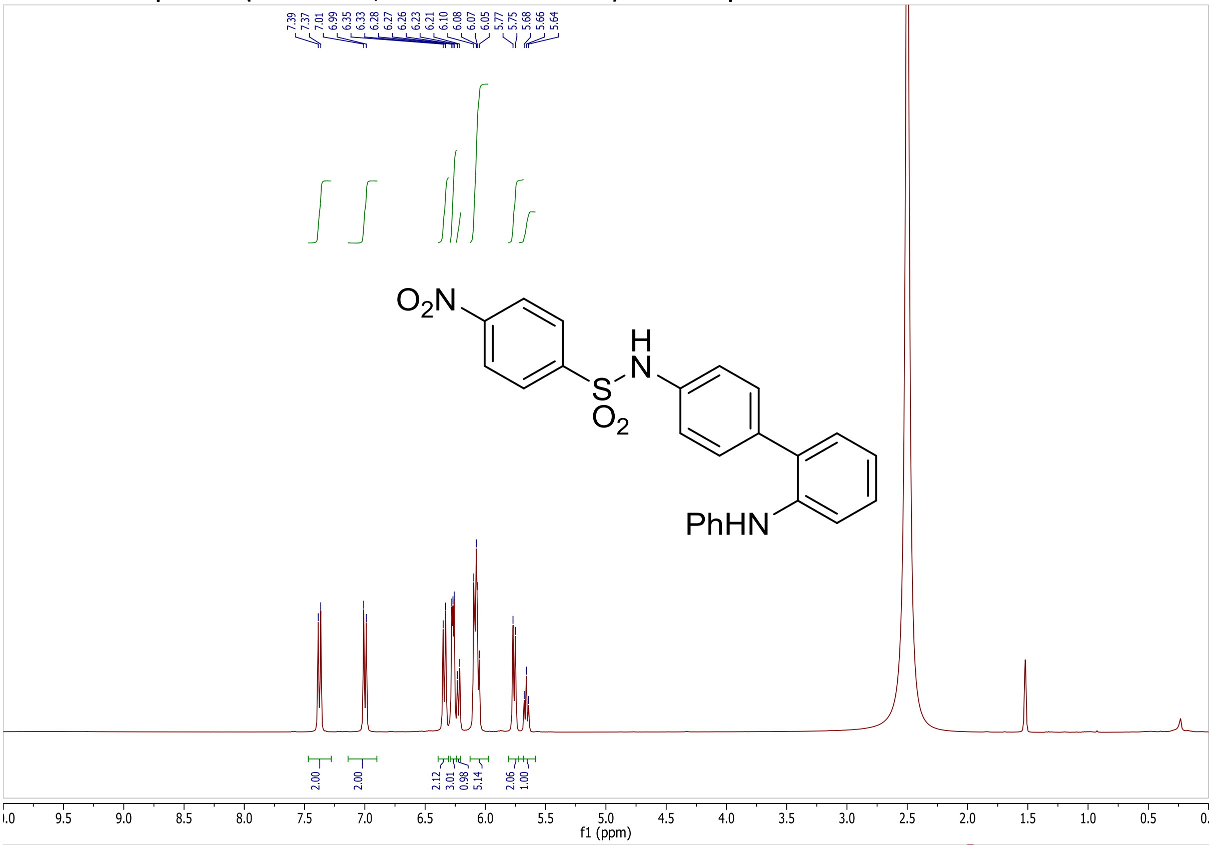

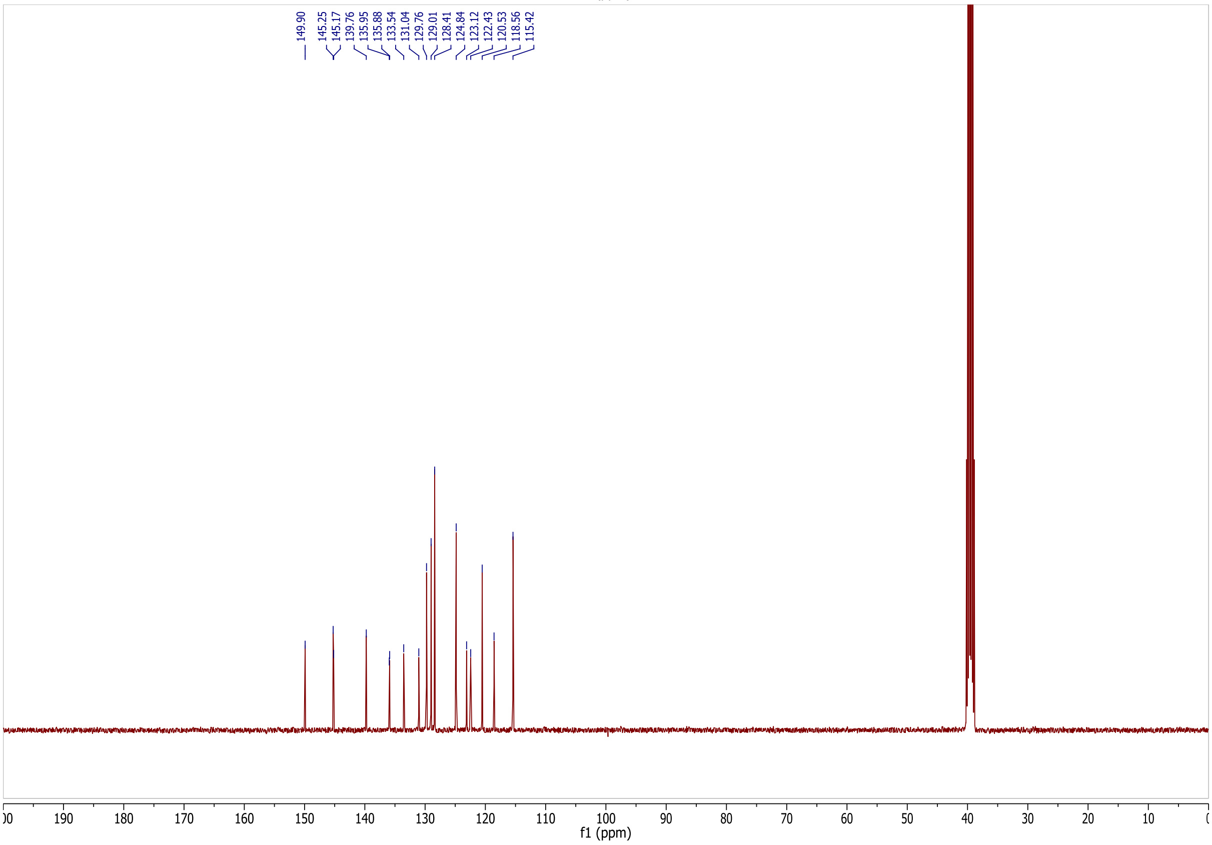

[1]H and [13]C Spectra ($CDCl_3$, 400 and 101 MHz) for compound 10

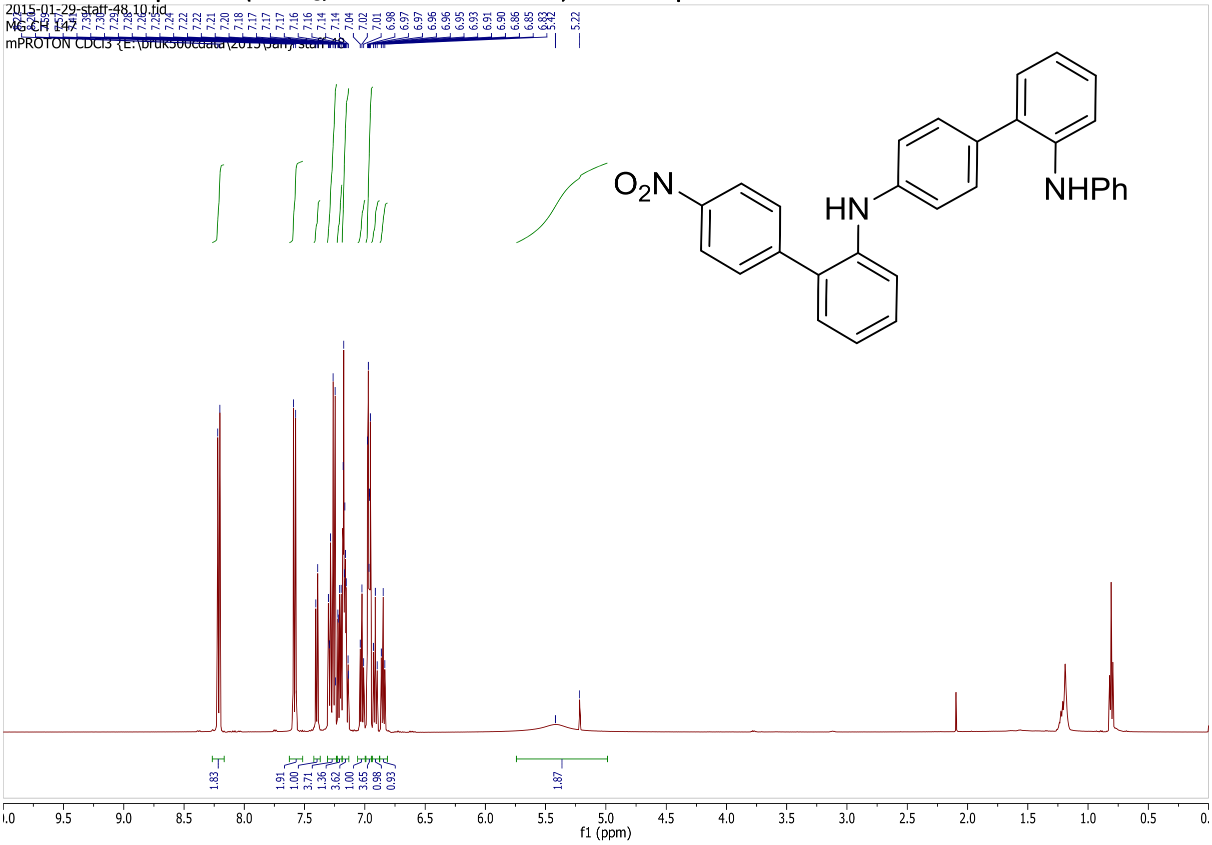

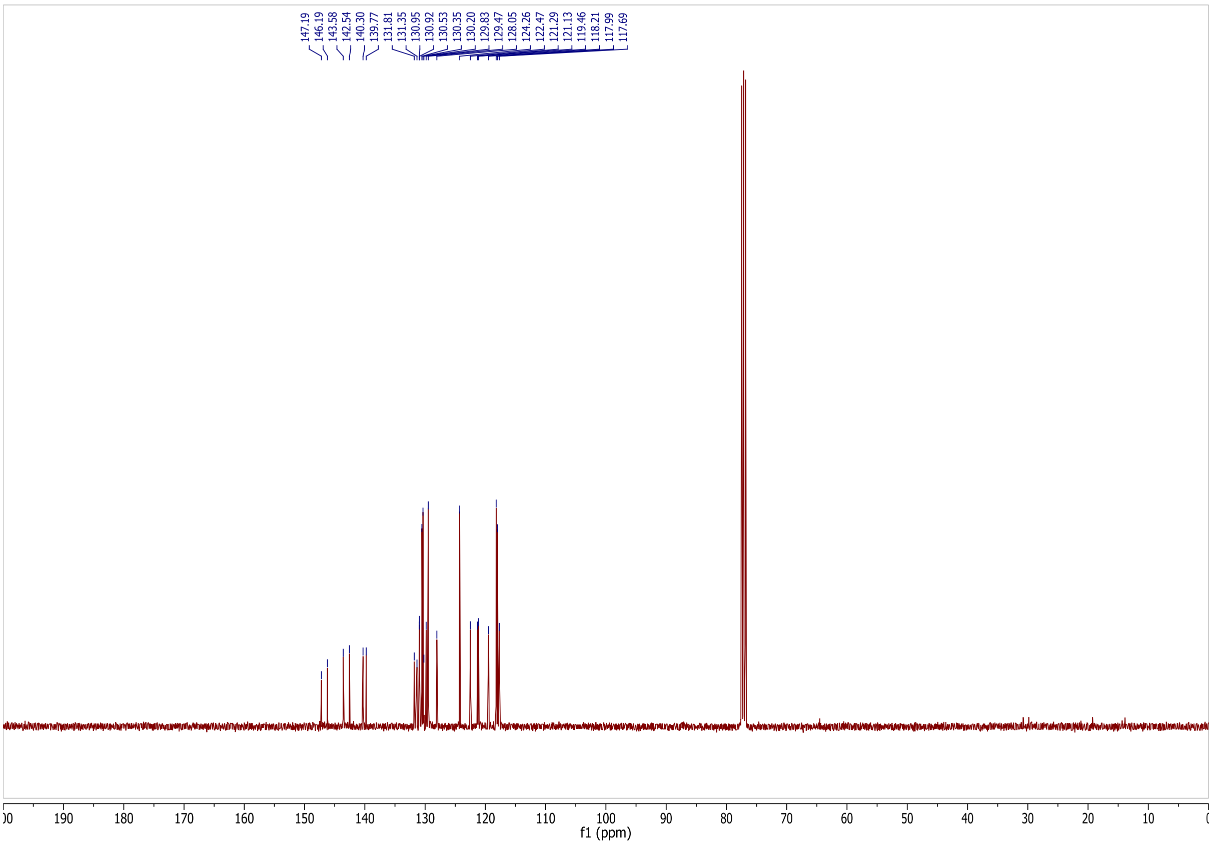

^1H and ^{13}C Spectra ($CDCl_3$, 400 and 101 MHz) for compound 11

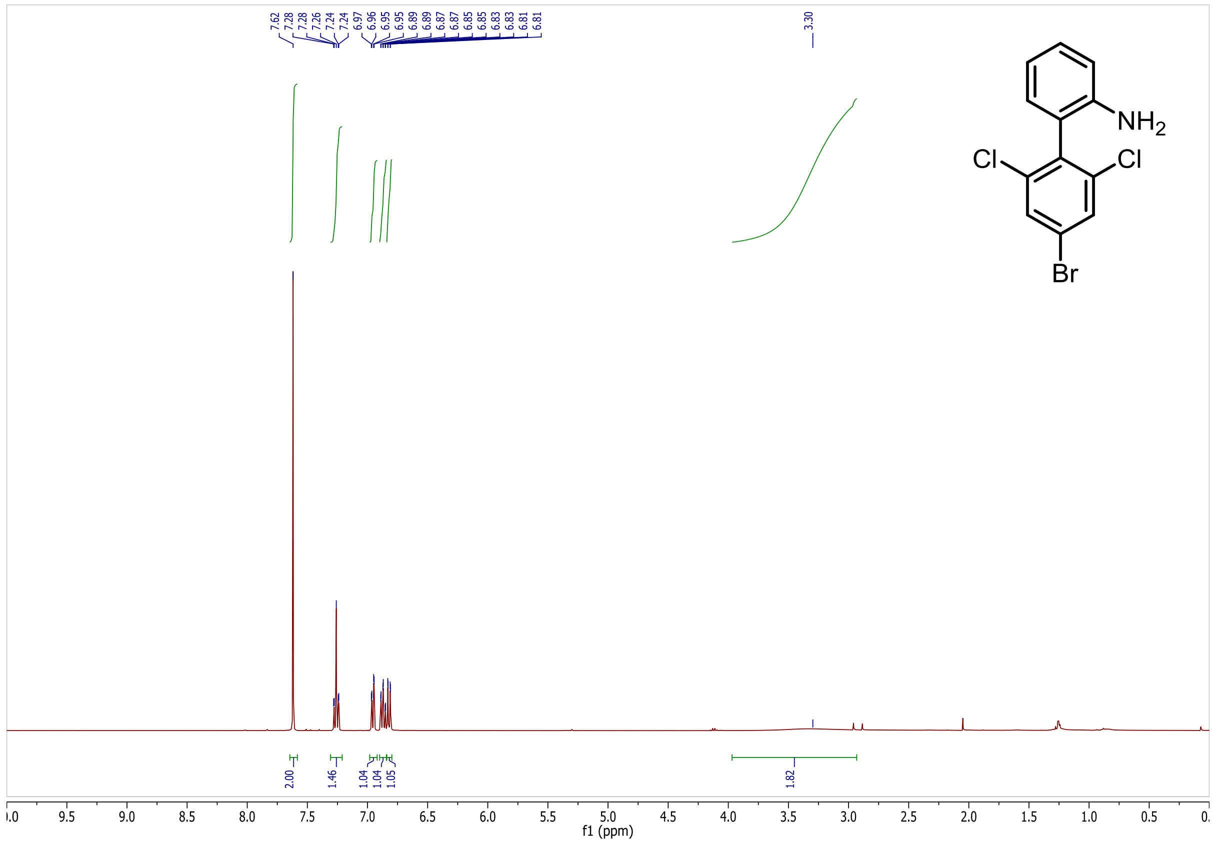

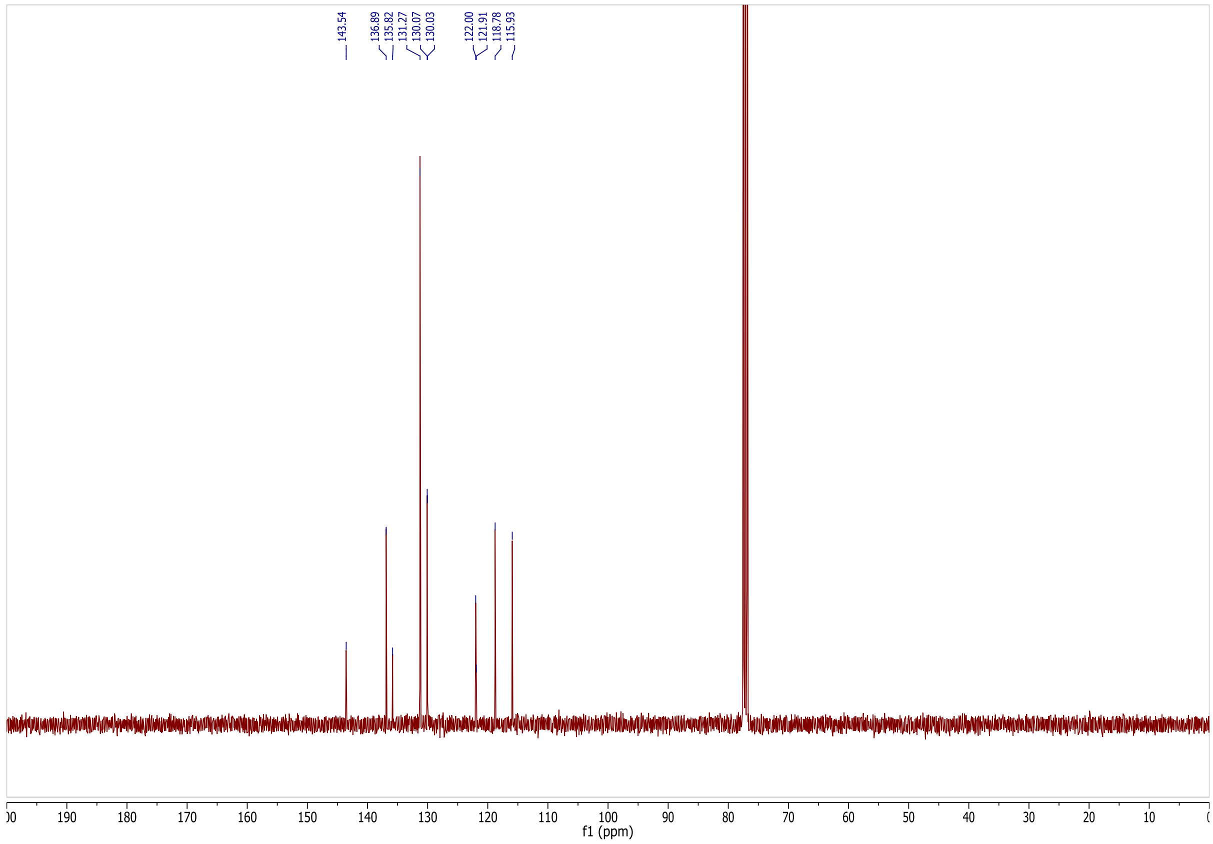

Supplement: Supplementary file 1 — Supplementary [file ANIE-55-2450-s001.pdf]
